# Supplementary figures and images for: Decoding the function of Atg13 phosphorylation reveals a role of Atg11 in bulk autophagy initiation
Source: EMBO Rep. 2024 Jan 17;25(2):813–31. doi: 10.1038/s44319-023-00055-9 (PMC10897315; doi:10.1038/s44319-023-00055-9)

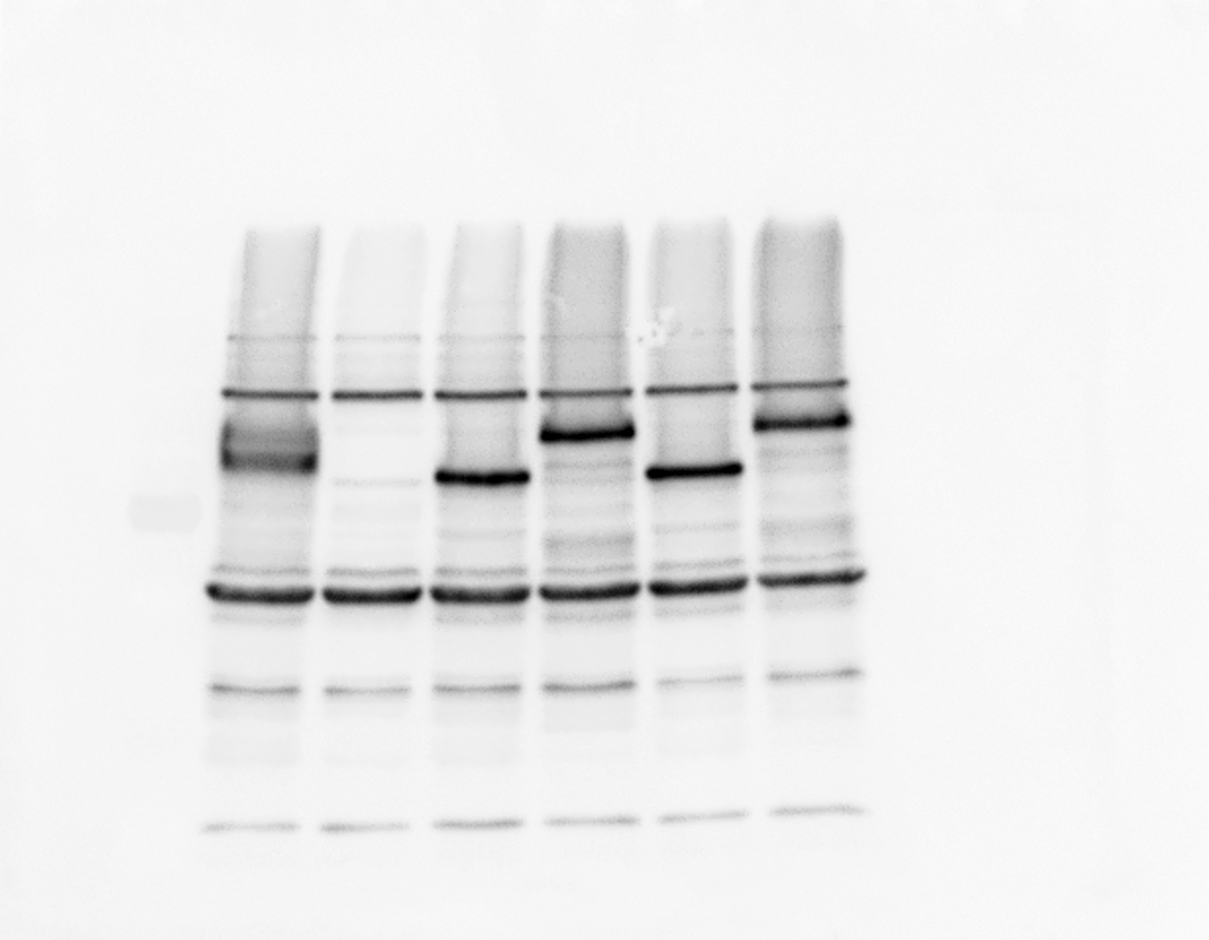

Supplement: Supplementary file 3 — Source Data Fig. 2 [file 44319_2023_55_MOESM3_ESM.zip › Figure 2/2A/western_Atg13.Tif]

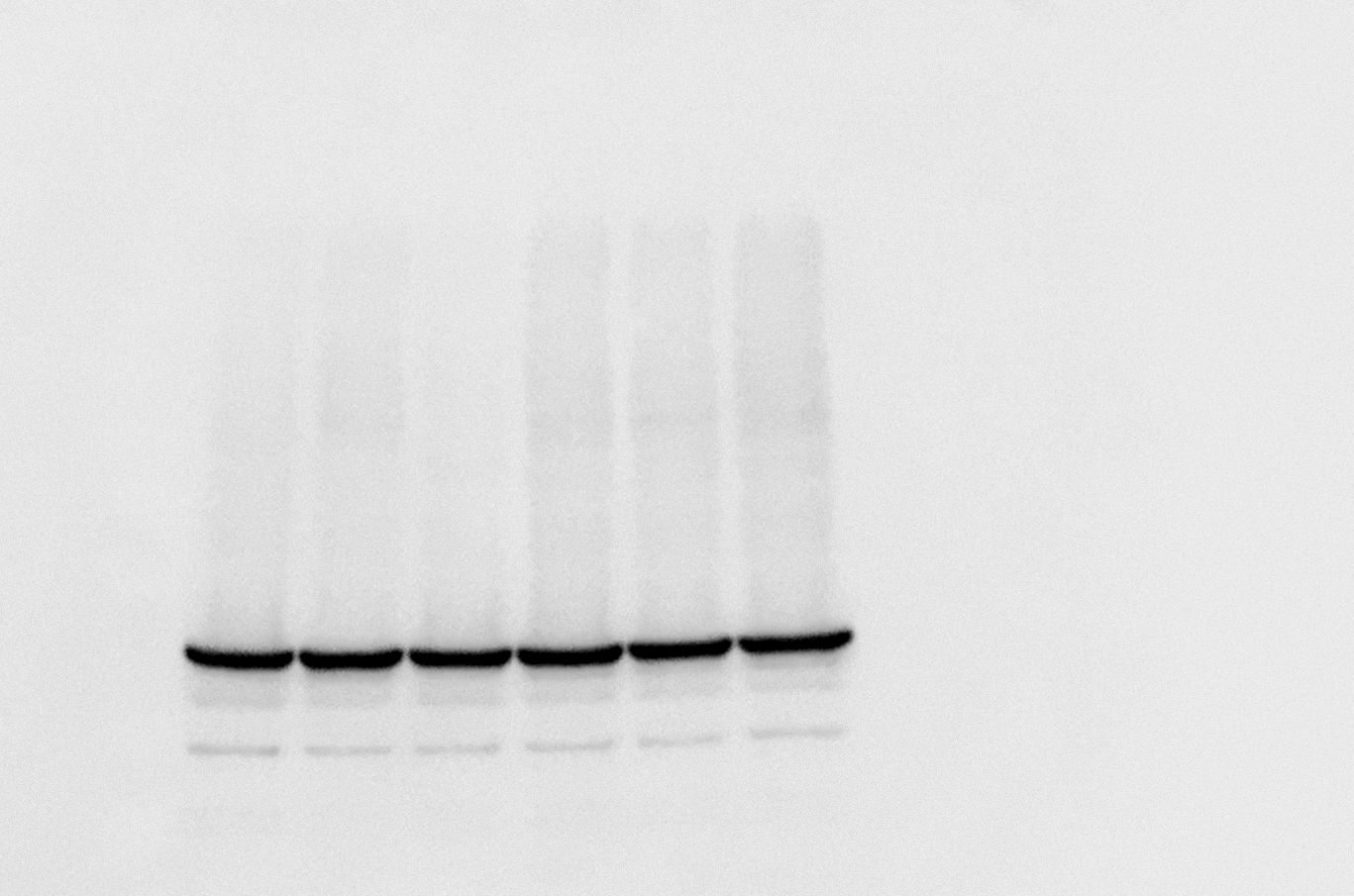

Supplement: Supplementary file 3 — Source Data Fig. 2 [file 44319_2023_55_MOESM3_ESM.zip › Figure 2/2A/western_Pgk1.Tif]

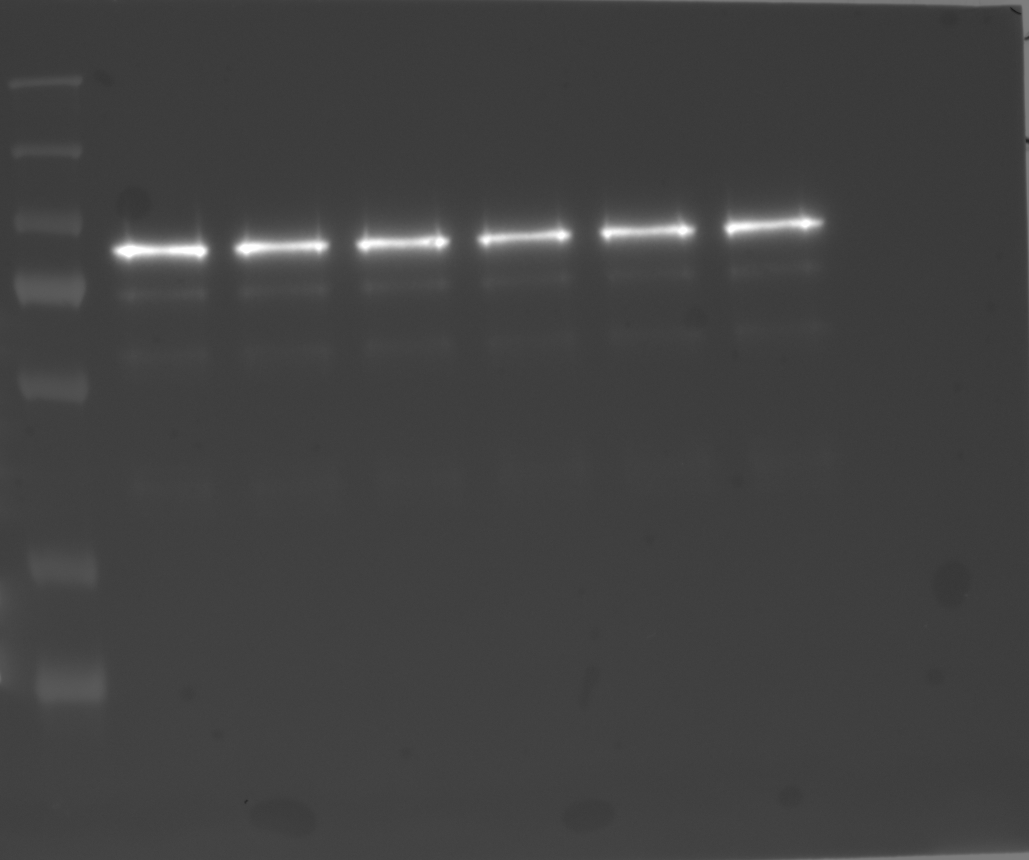

Supplement: Supplementary file 3 — Source Data Fig. 2 [file 44319_2023_55_MOESM3_ESM.zip › Figure 2/2C/Input_Atg17.Tif]

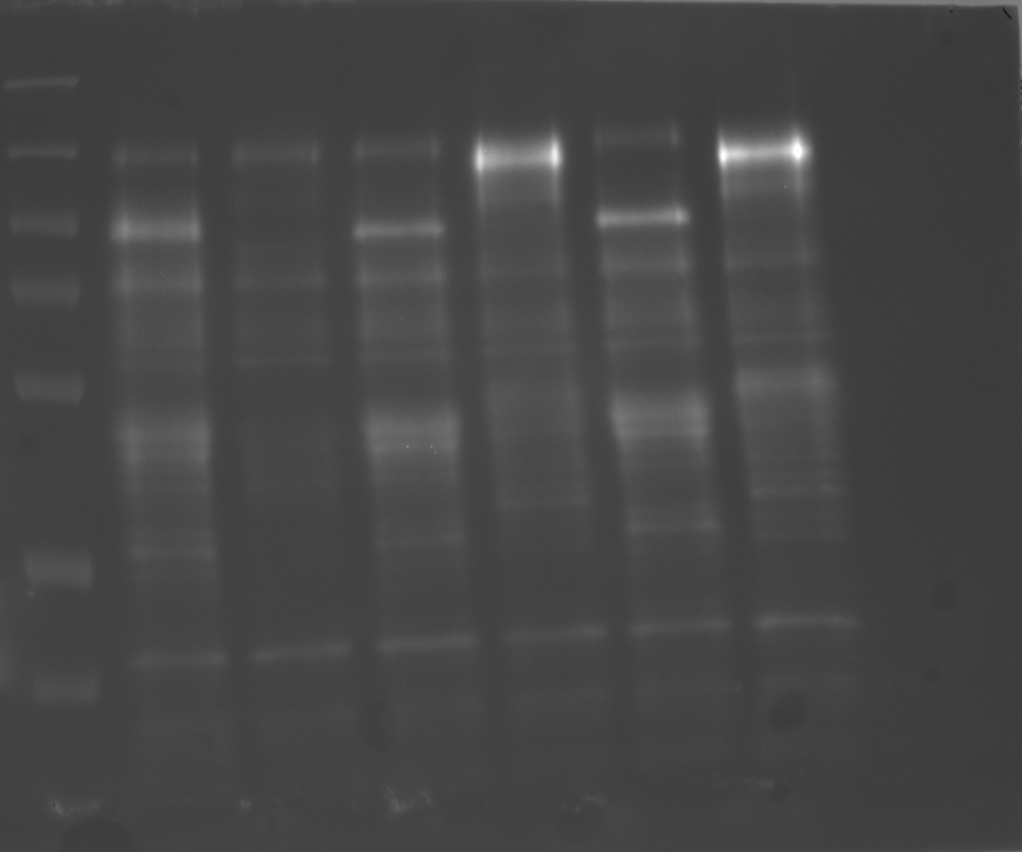

Supplement: Supplementary file 3 — Source Data Fig. 2 [file 44319_2023_55_MOESM3_ESM.zip › Figure 2/2C/Input_Atg13.Tif]

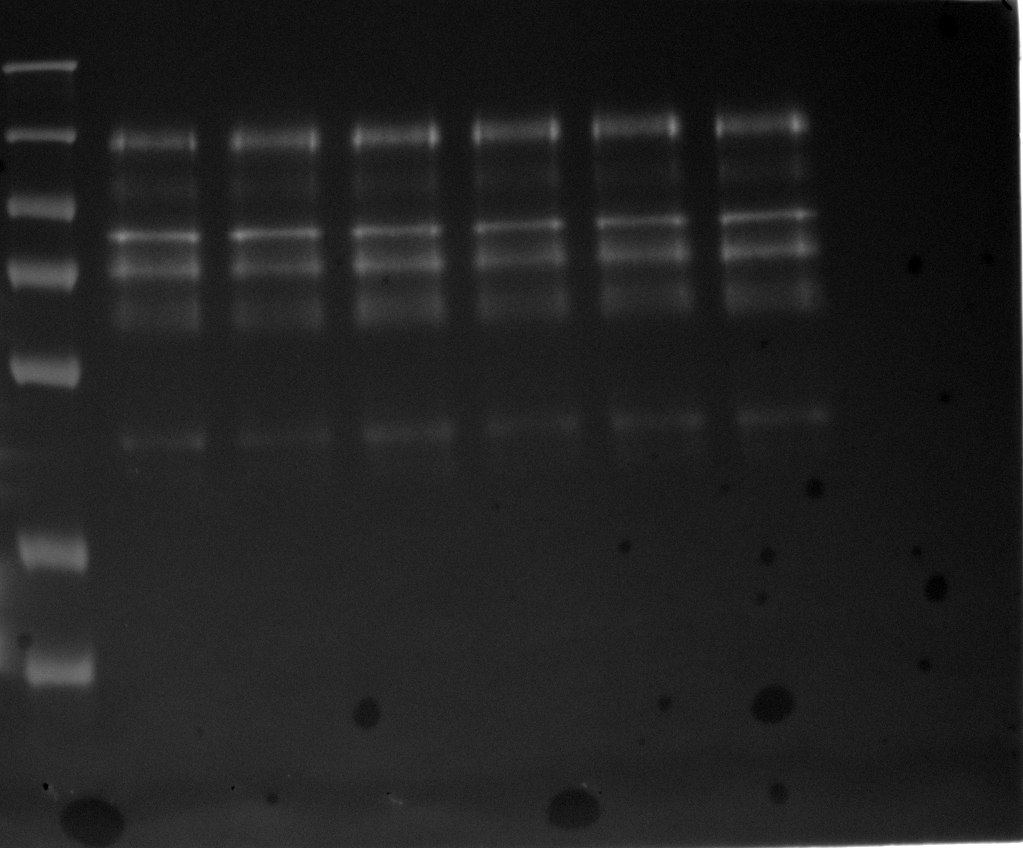

Supplement: Supplementary file 3 — Source Data Fig. 2 [file 44319_2023_55_MOESM3_ESM.zip › Figure 2/2C/Input_Atg1.Tif]

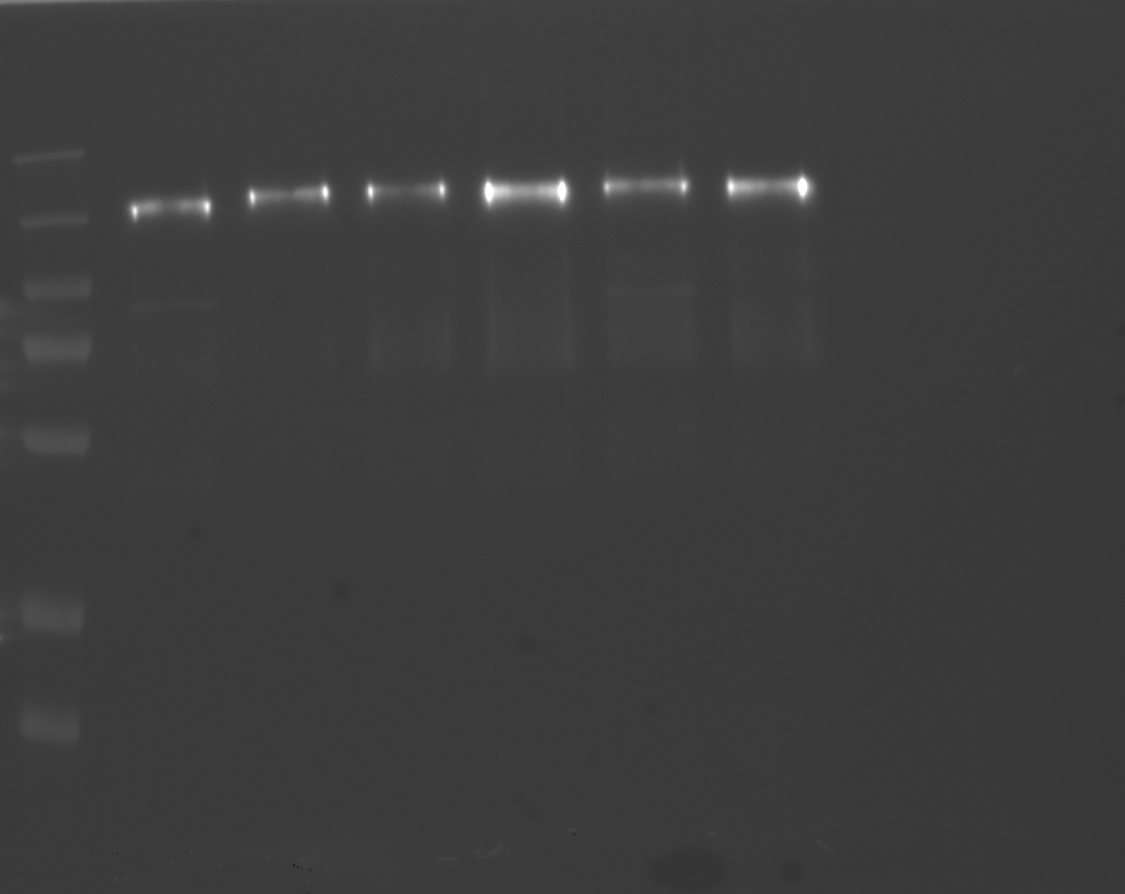

Supplement: Supplementary file 3 — Source Data Fig. 2 [file 44319_2023_55_MOESM3_ESM.zip › Figure 2/2C/IP_Atg1.Tif]

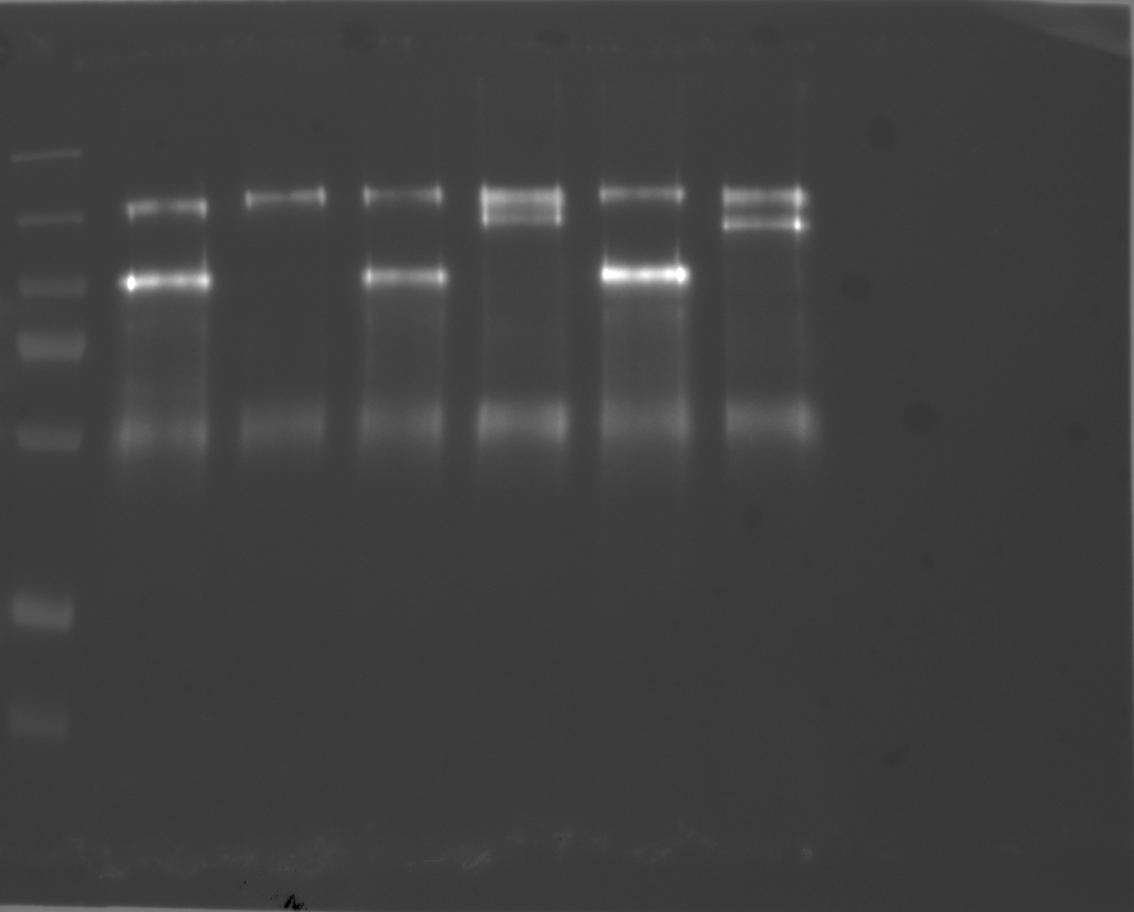

Supplement: Supplementary file 3 — Source Data Fig. 2 [file 44319_2023_55_MOESM3_ESM.zip › Figure 2/2C/IP_Atg13.tif]

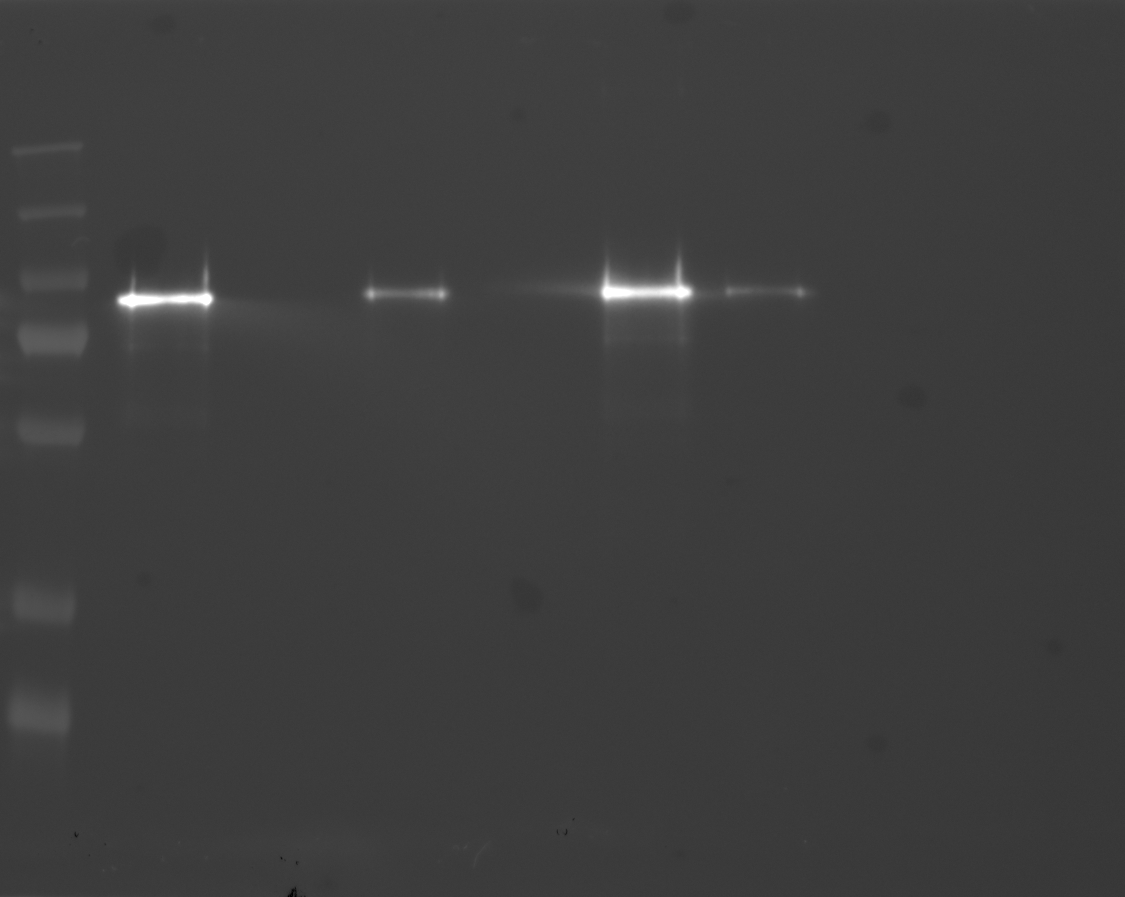

Supplement: Supplementary file 3 — Source Data Fig. 2 [file 44319_2023_55_MOESM3_ESM.zip › Figure 2/2C/IP_Atg17.Tif]

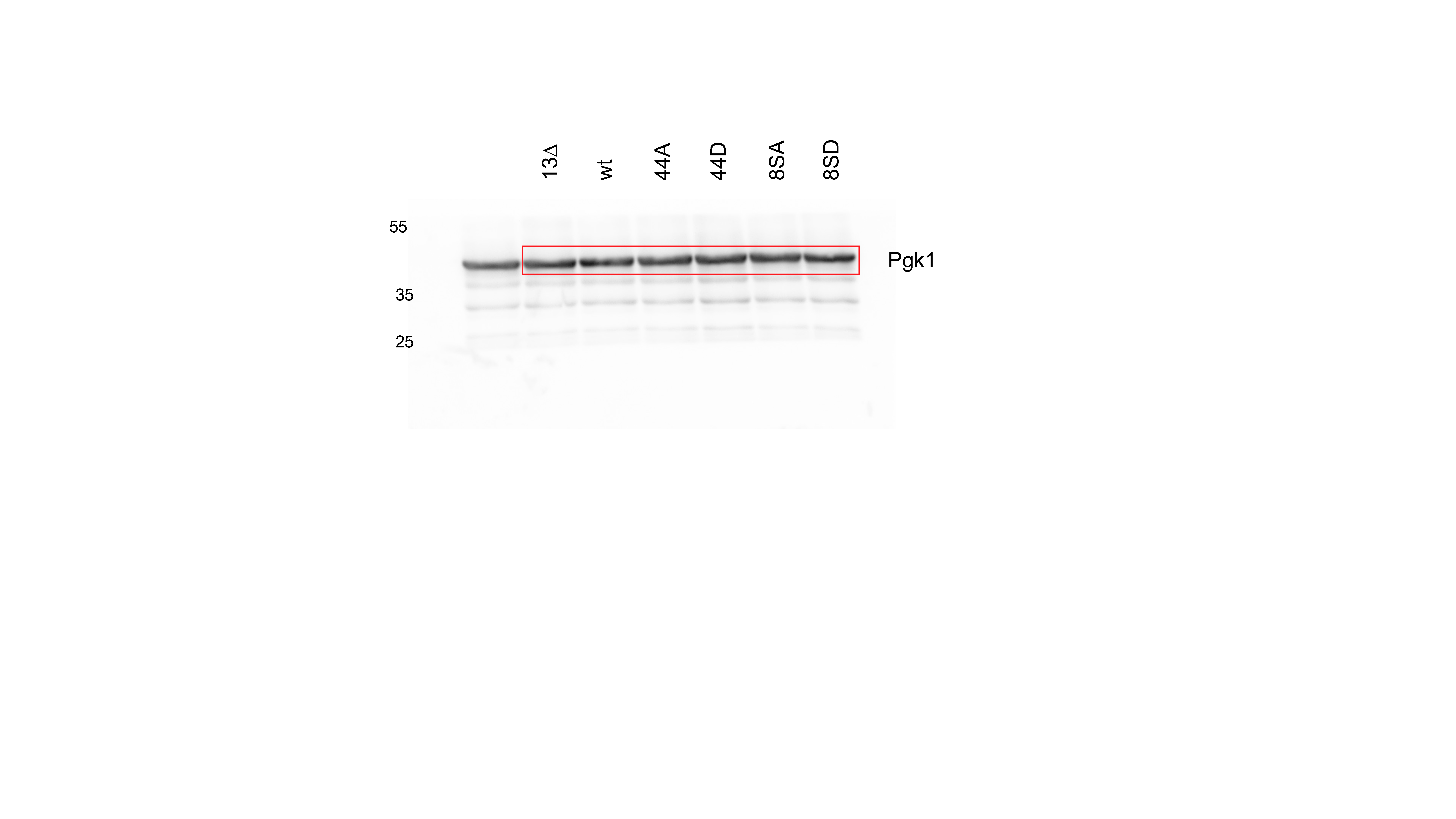

Supplement: Supplementary file 3 — Source Data Fig. 2 [file 44319_2023_55_MOESM3_ESM.zip › Figure 2/2D/western_Pgk1_marked.tiff]

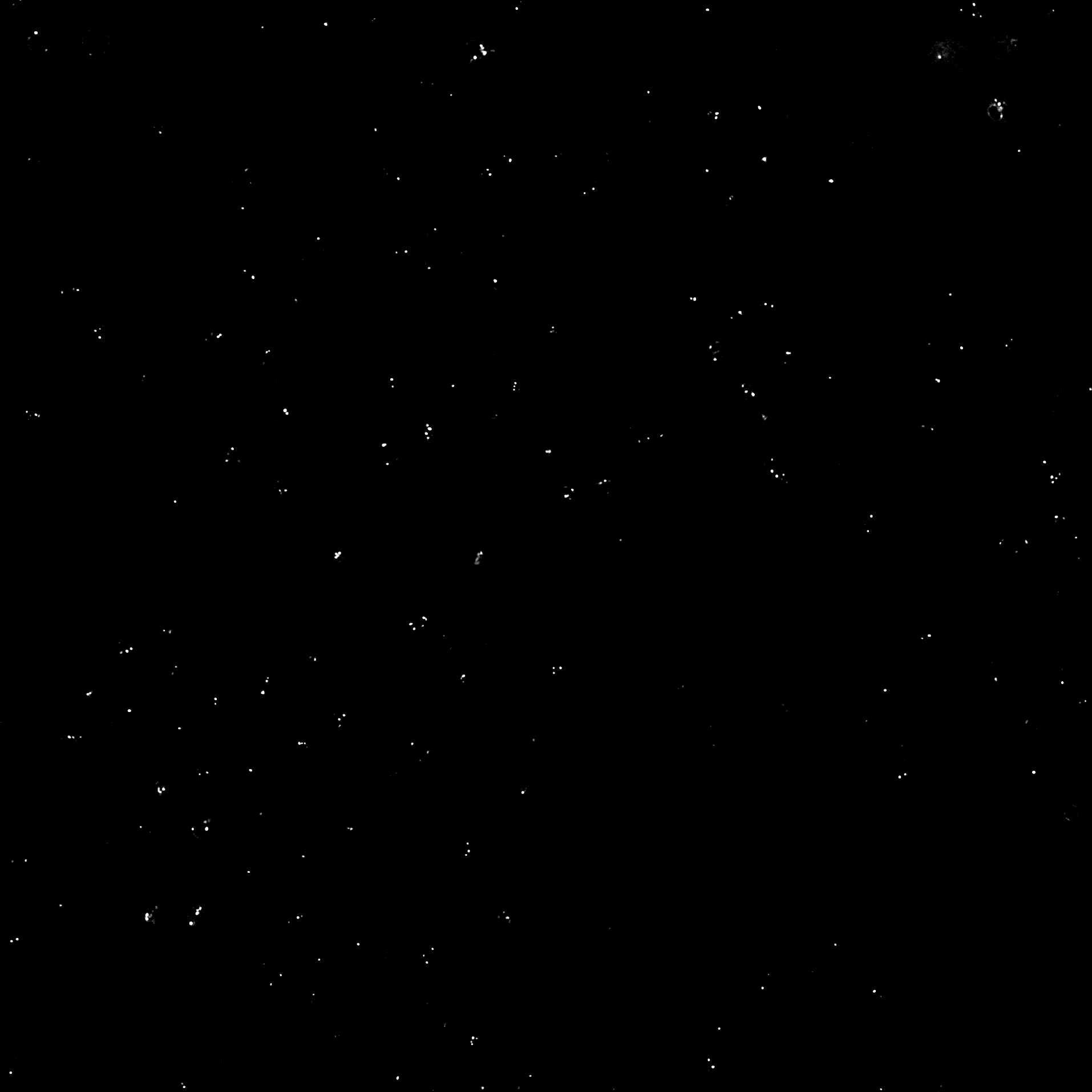

Supplement: Supplementary file 3 — Source Data Fig. 2 [file 44319_2023_55_MOESM3_ESM.zip › Figure 2/2G/44A-GFP_8D13D19D/GFP.tif]

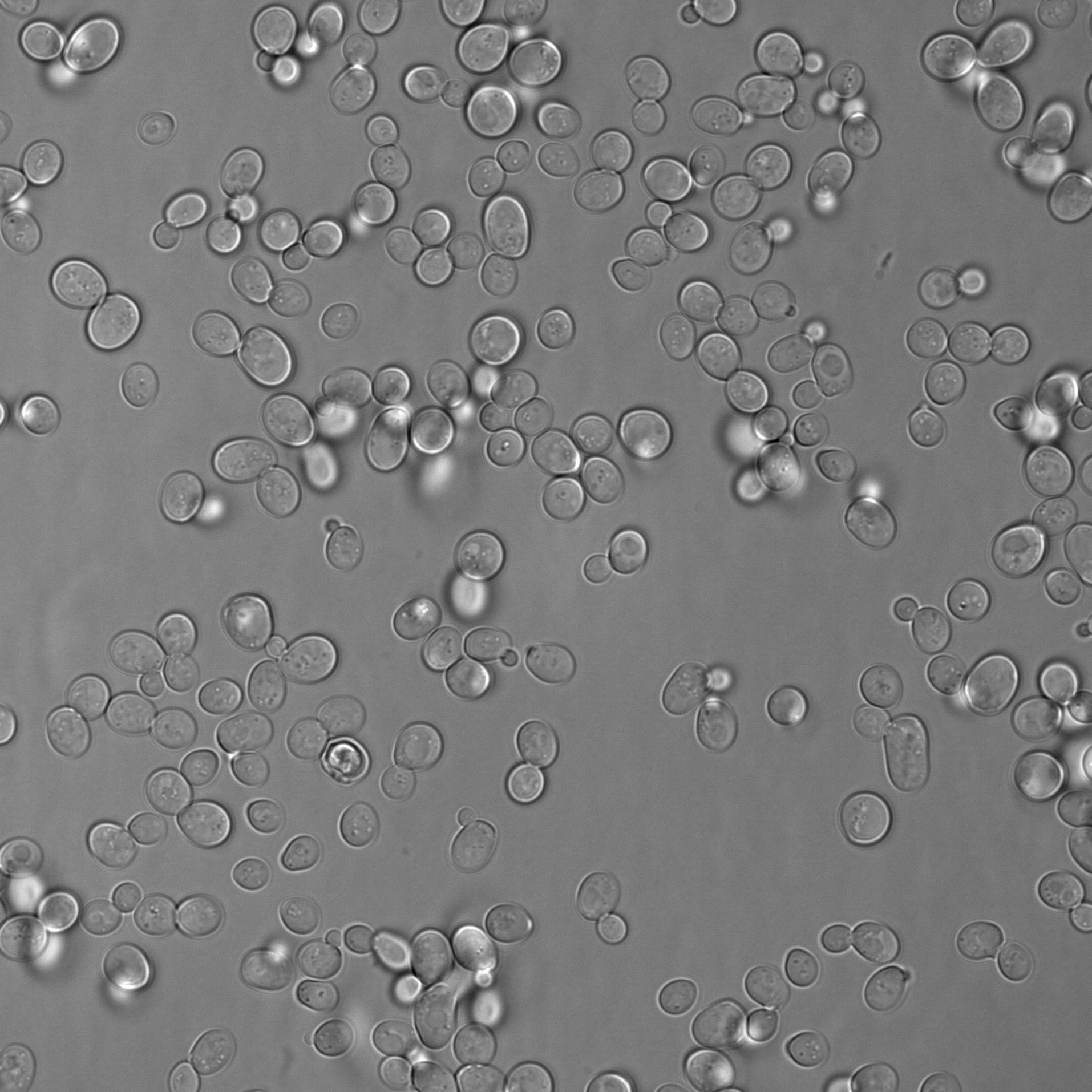

Supplement: Supplementary file 3 — Source Data Fig. 2 [file 44319_2023_55_MOESM3_ESM.zip › Figure 2/2G/44A-GFP_8D13D19D/BF.tif]

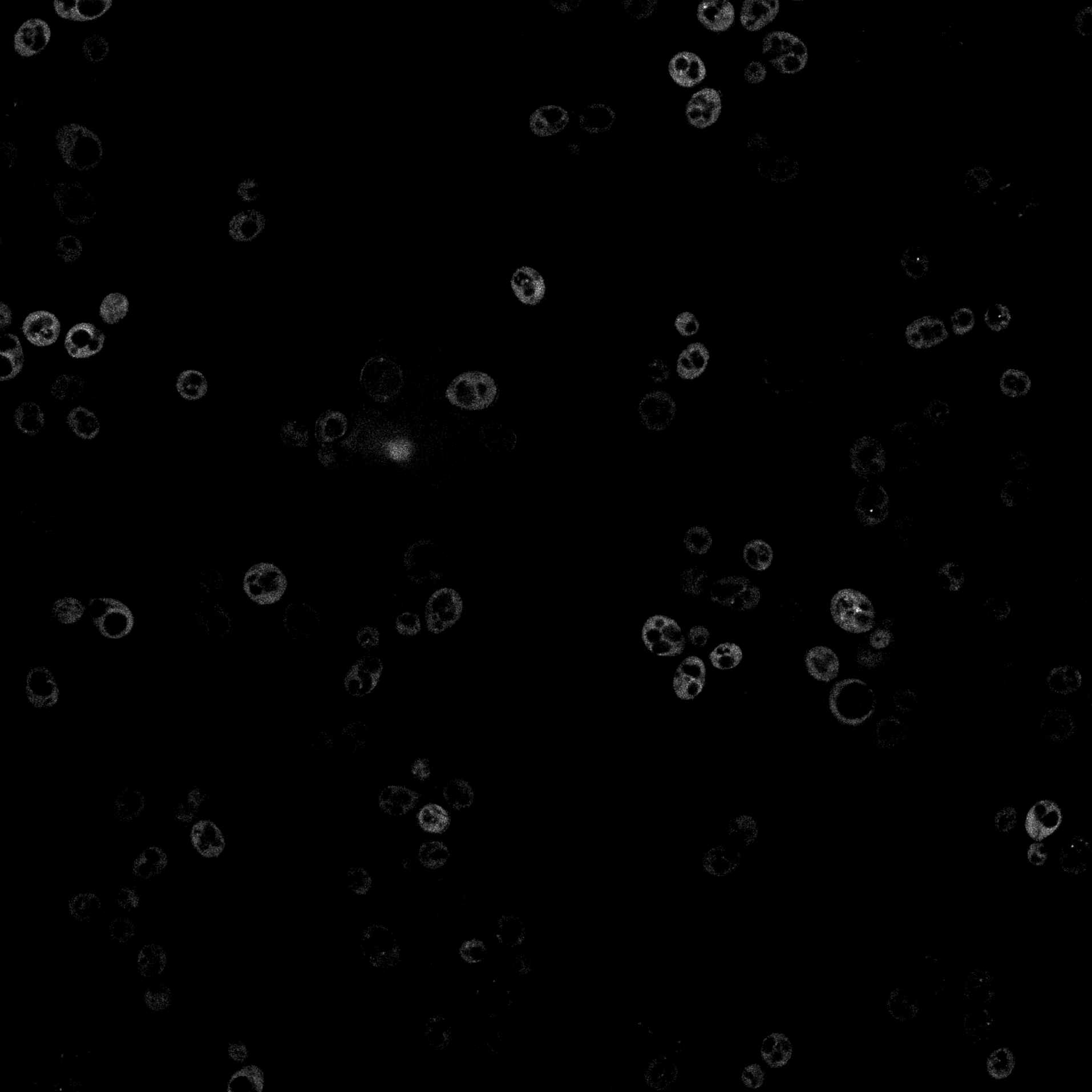

Supplement: Supplementary file 3 — Source Data Fig. 2 [file 44319_2023_55_MOESM3_ESM.zip › Figure 2/2G/44D-GFP_8D13D19D/GFP.tif]

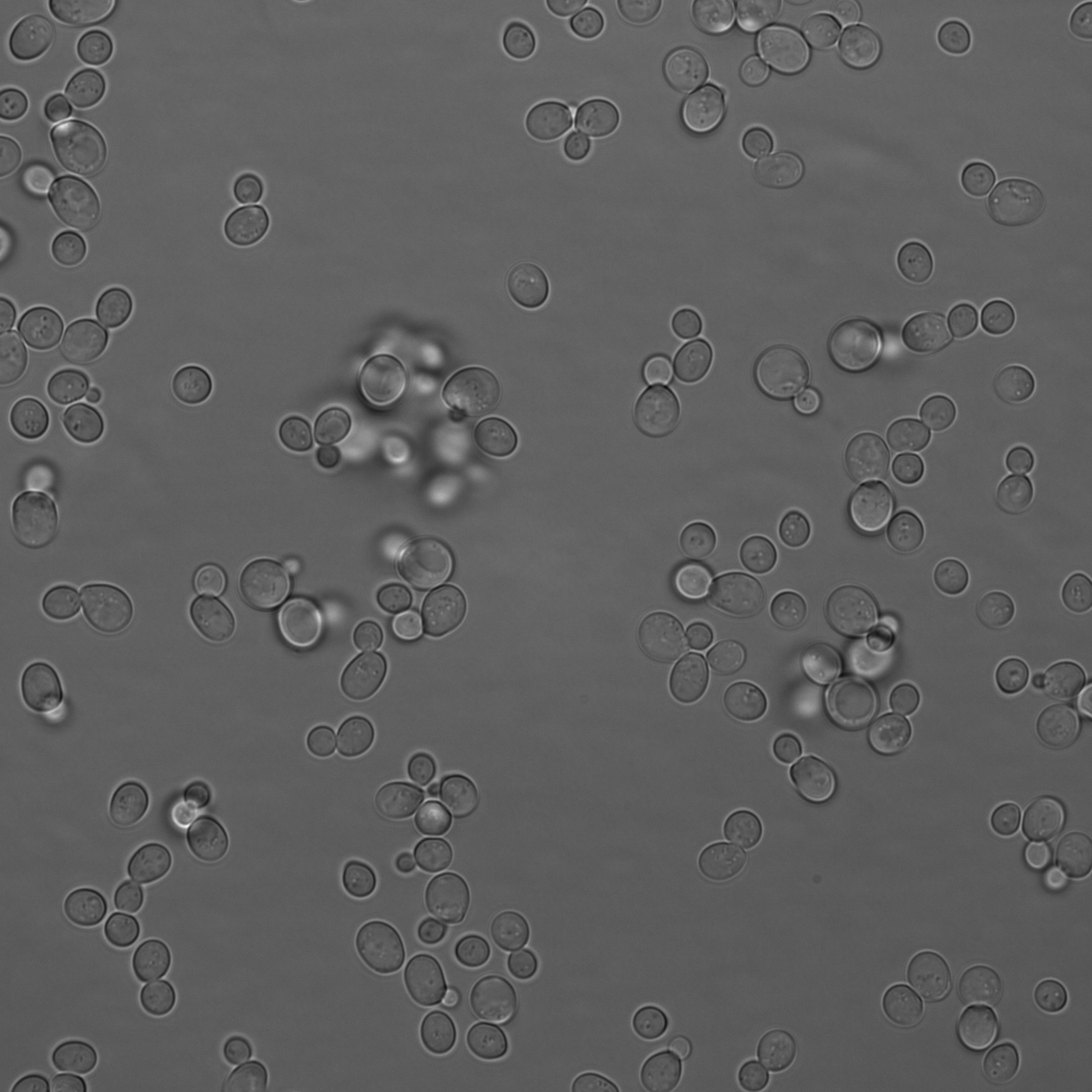

Supplement: Supplementary file 3 — Source Data Fig. 2 [file 44319_2023_55_MOESM3_ESM.zip › Figure 2/2G/44D-GFP_8D13D19D/BF.tif]

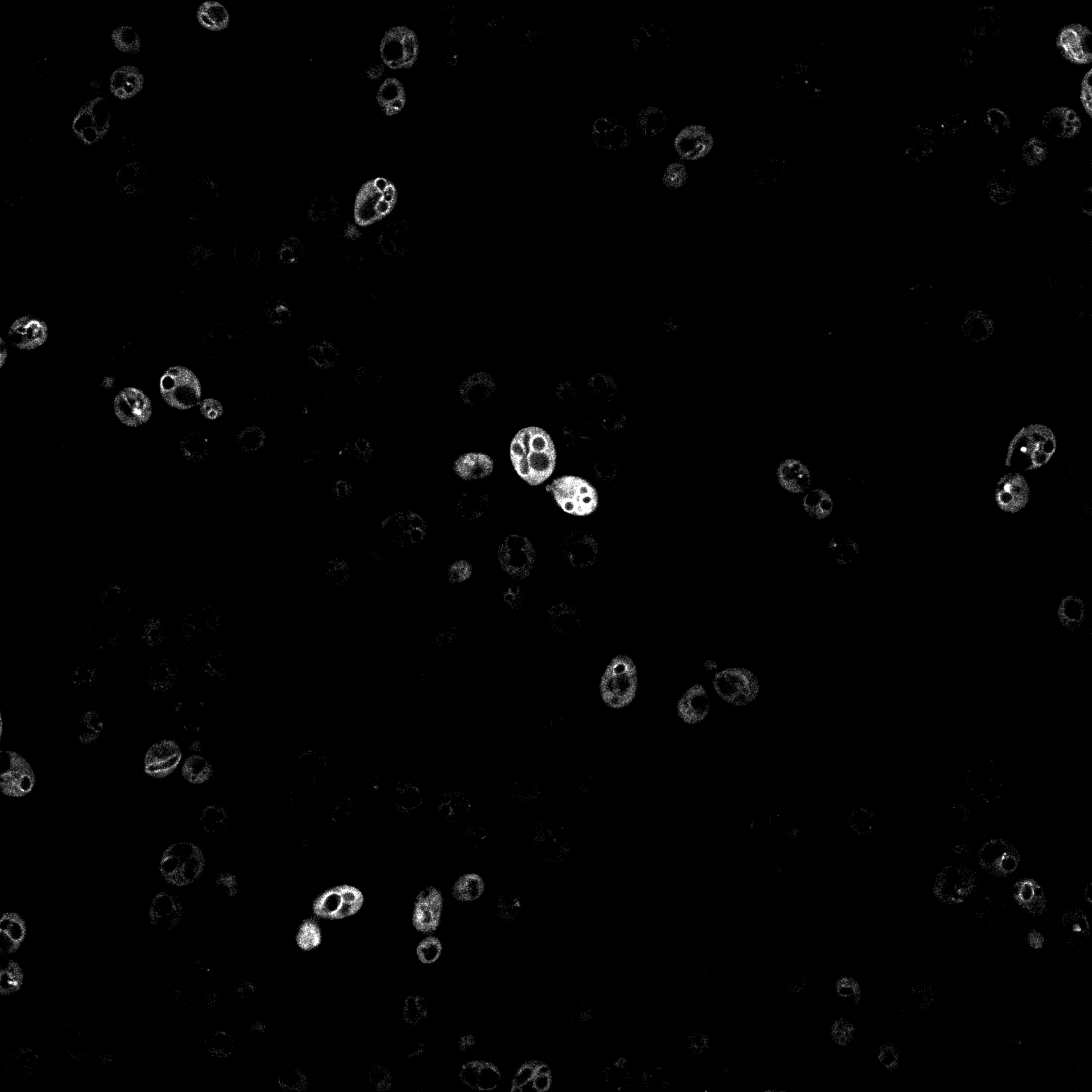

Supplement: Supplementary file 3 — Source Data Fig. 2 [file 44319_2023_55_MOESM3_ESM.zip › Figure 2/2G/13wt-GFP_8D13D17D19D/GFP.tif]

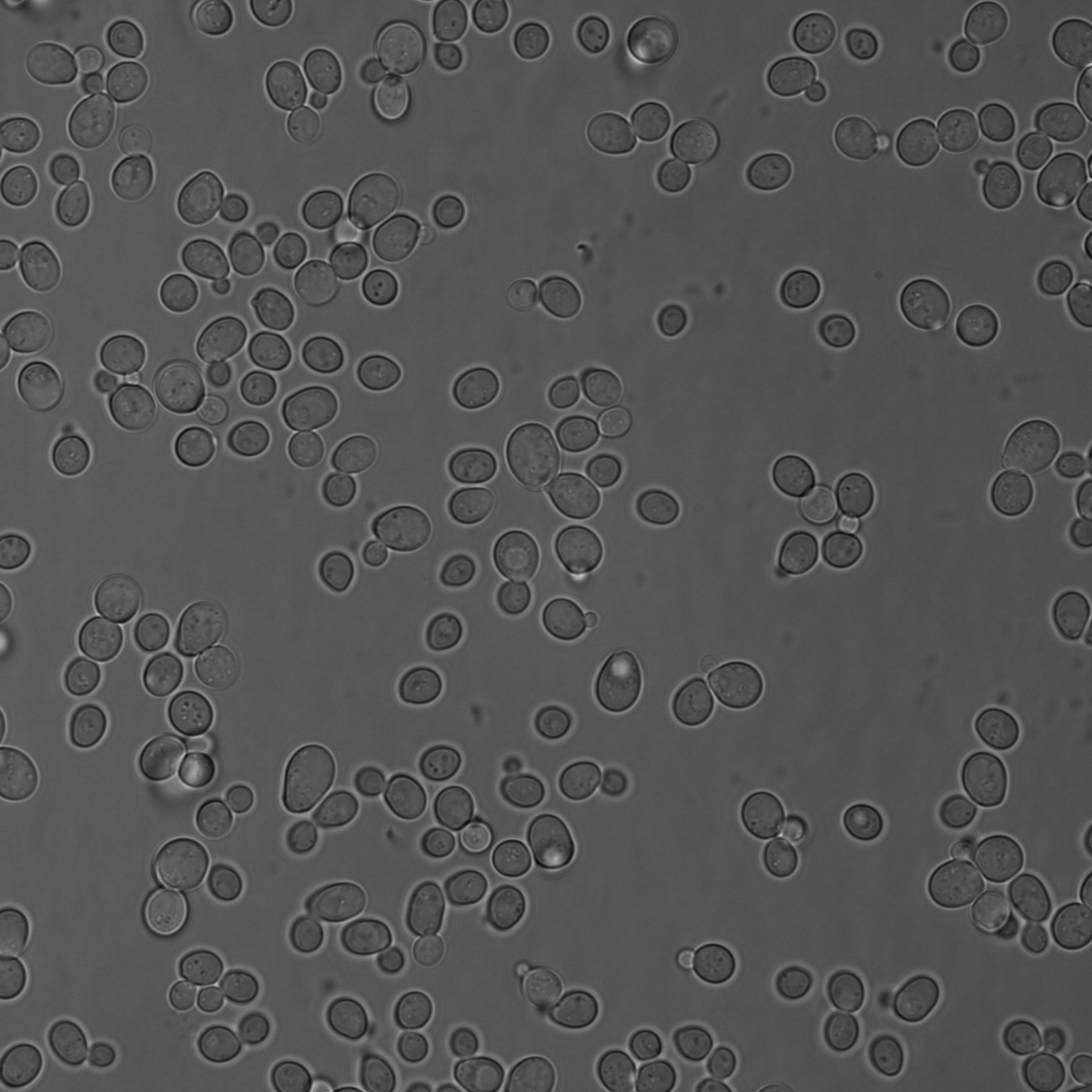

Supplement: Supplementary file 3 — Source Data Fig. 2 [file 44319_2023_55_MOESM3_ESM.zip › Figure 2/2G/13wt-GFP_8D13D17D19D/BF.tif]

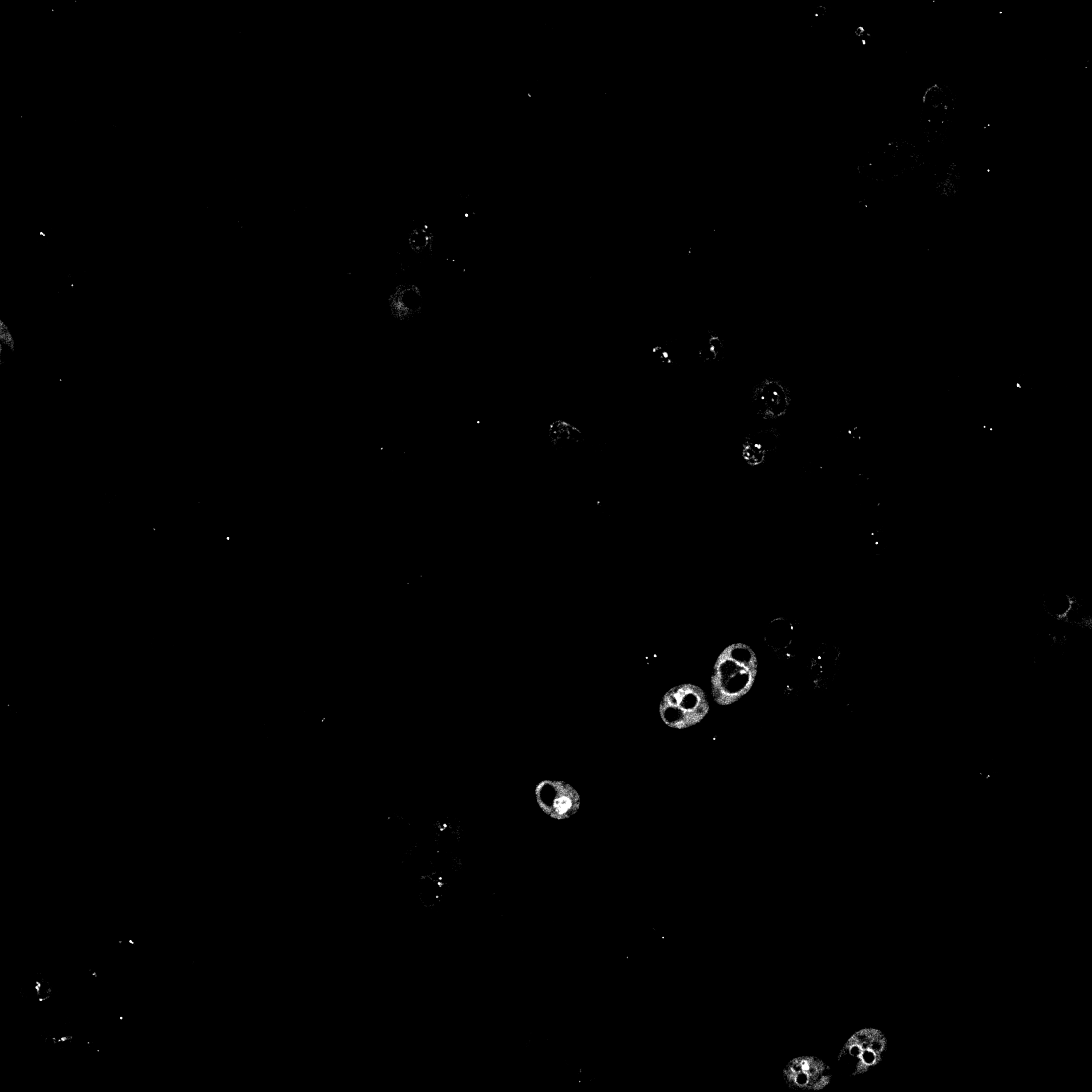

Supplement: Supplementary file 3 — Source Data Fig. 2 [file 44319_2023_55_MOESM3_ESM.zip › Figure 2/2G/13wt-GFP_8D13D19D/GFP.tif]

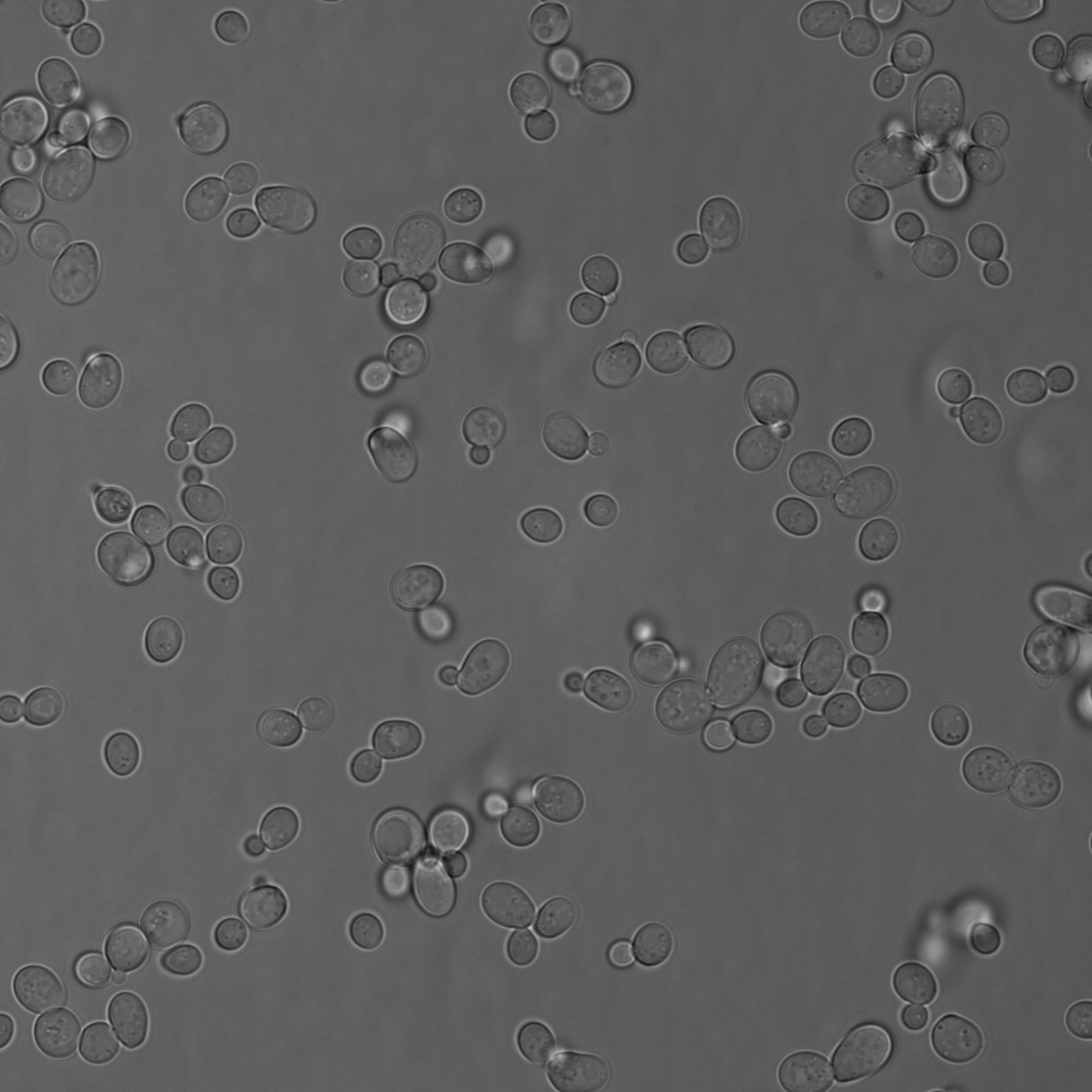

Supplement: Supplementary file 3 — Source Data Fig. 2 [file 44319_2023_55_MOESM3_ESM.zip › Figure 2/2G/13wt-GFP_8D13D19D/BFP.tif]

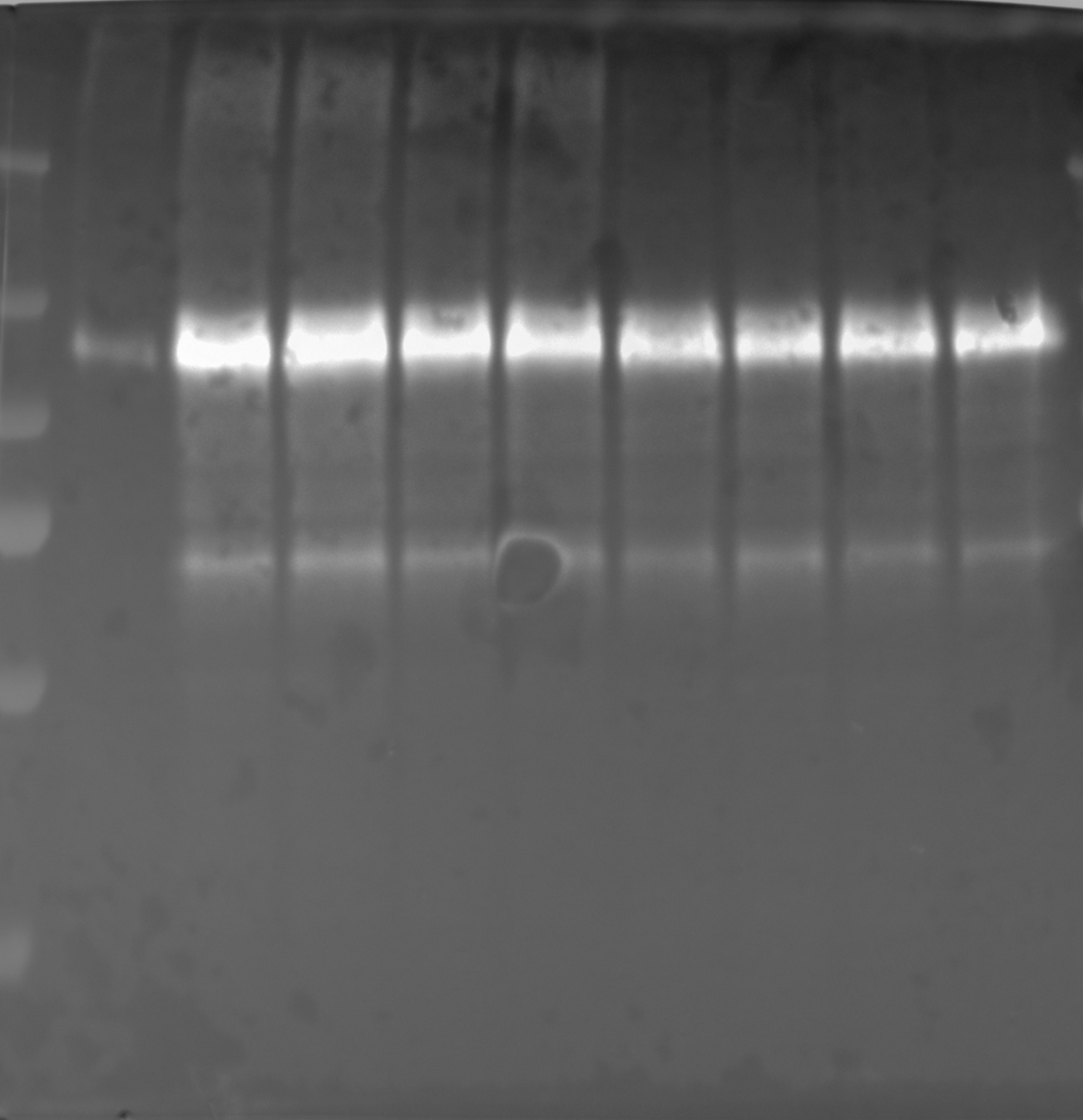

Supplement: Supplementary file 4 — Source Data Fig. 3 [file 44319_2023_55_MOESM4_ESM.zip › Figure 3/3B/Western_Sch9p.Tif]

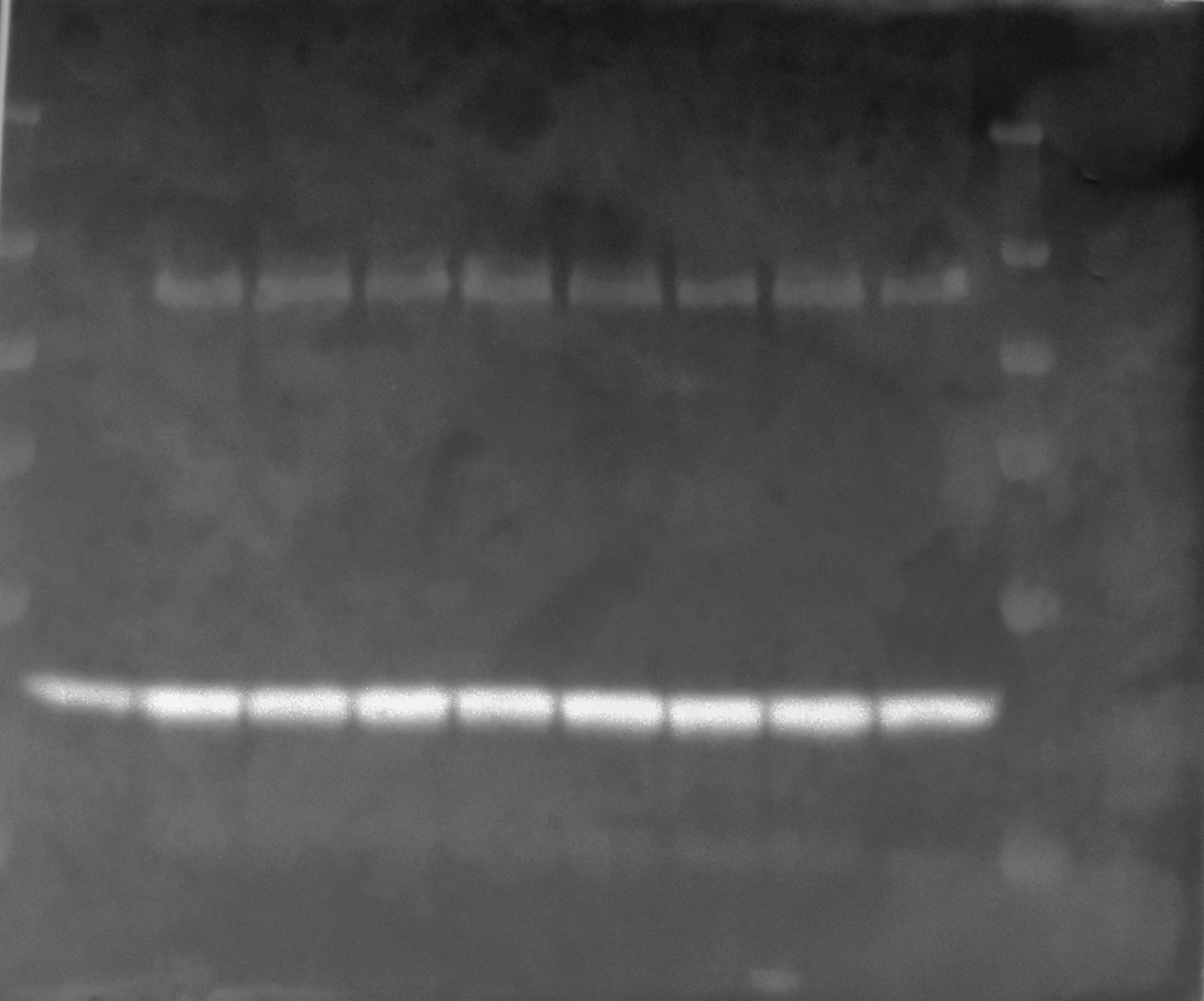

Supplement: Supplementary file 4 — Source Data Fig. 3 [file 44319_2023_55_MOESM4_ESM.zip › Figure 3/3B/Western_Pgk1.Tif]

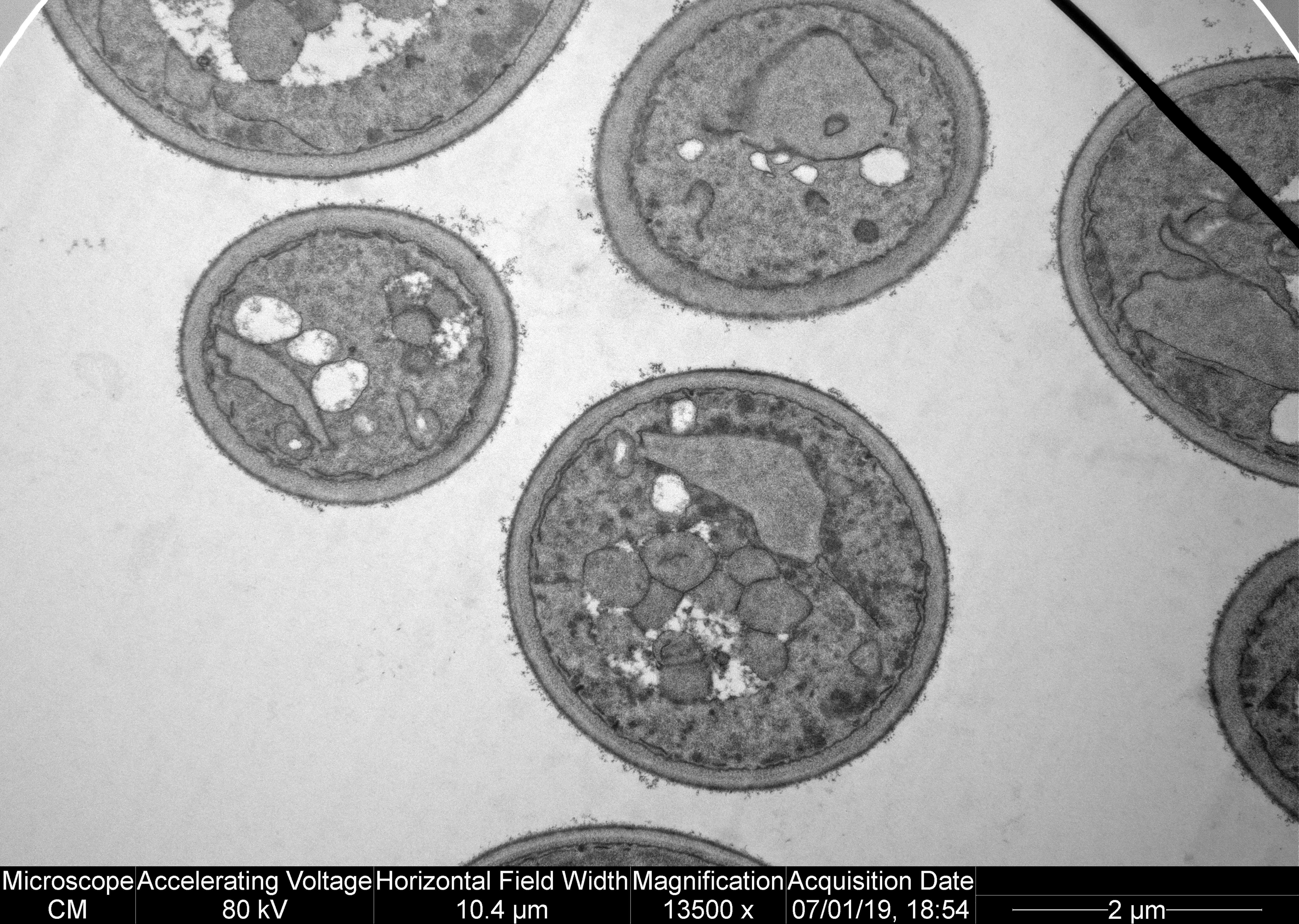

Supplement: Supplementary file 4 — Source Data Fig. 3 [file 44319_2023_55_MOESM4_ESM.zip › Figure 3/3D/EM_WT_Starvation.tif]

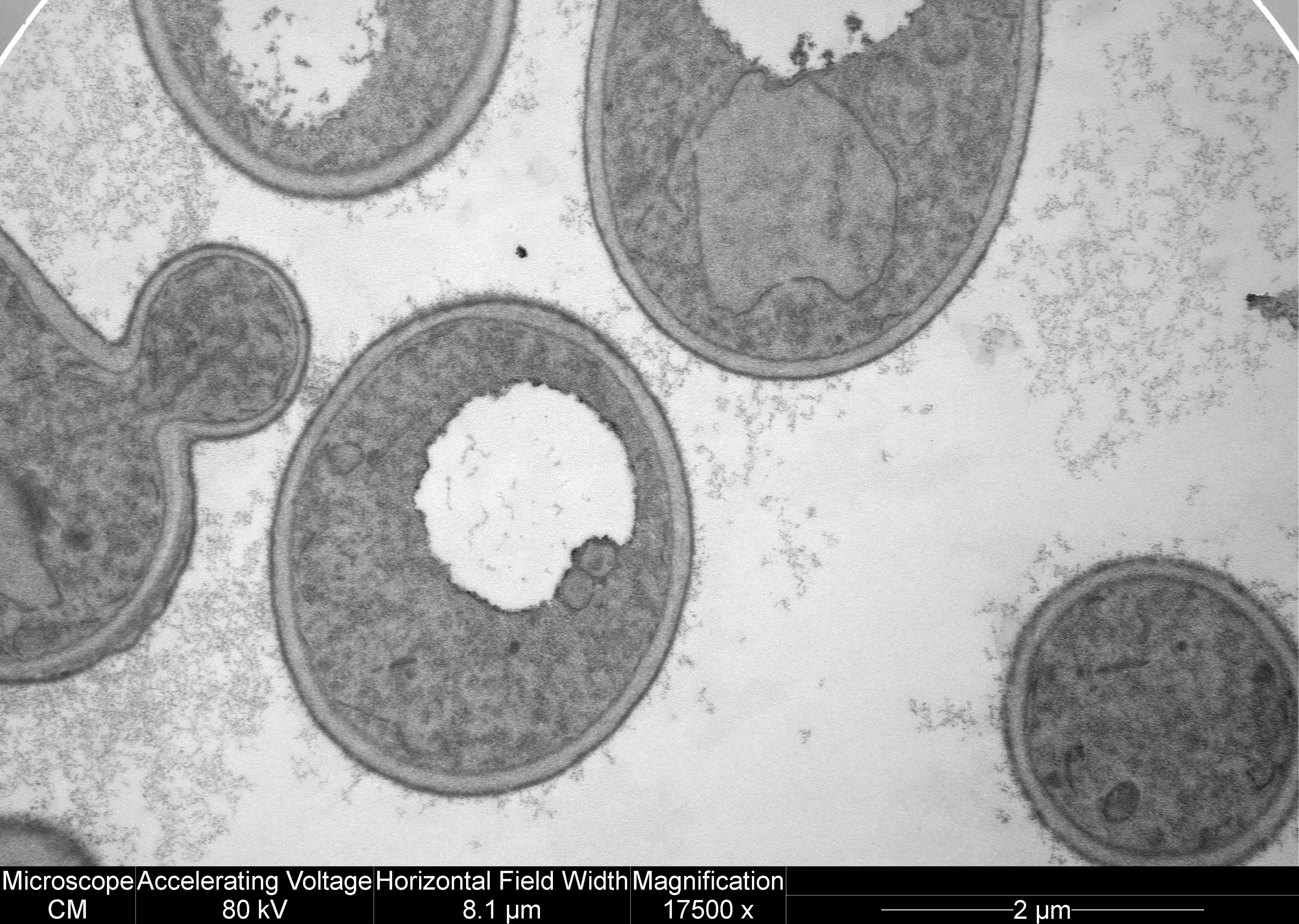

Supplement: Supplementary file 4 — Source Data Fig. 3 [file 44319_2023_55_MOESM4_ESM.zip › Figure 3/3D/EM_44A_Rich.tif]

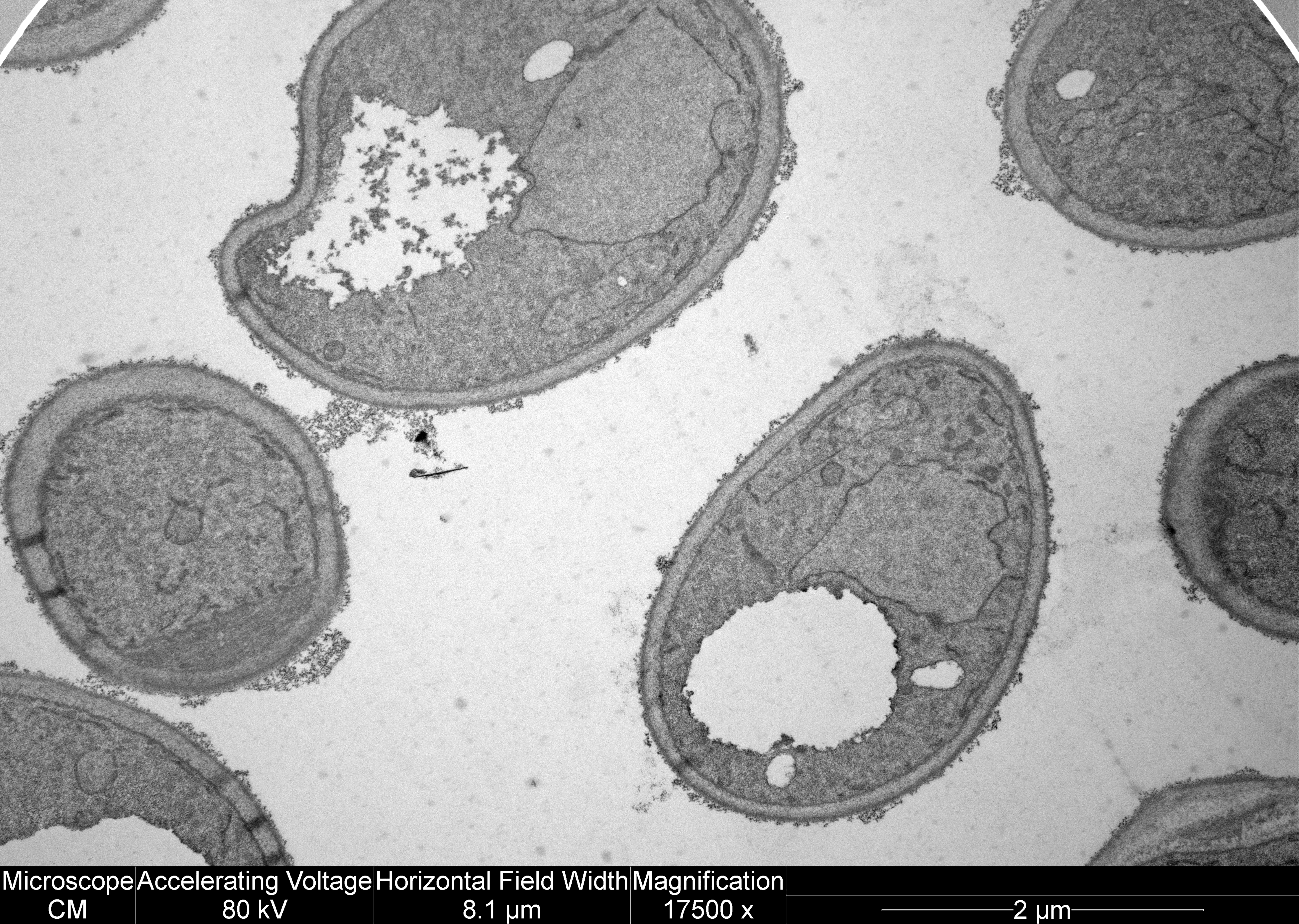

Supplement: Supplementary file 4 — Source Data Fig. 3 [file 44319_2023_55_MOESM4_ESM.zip › Figure 3/3D/EM_WT_rich.tif]

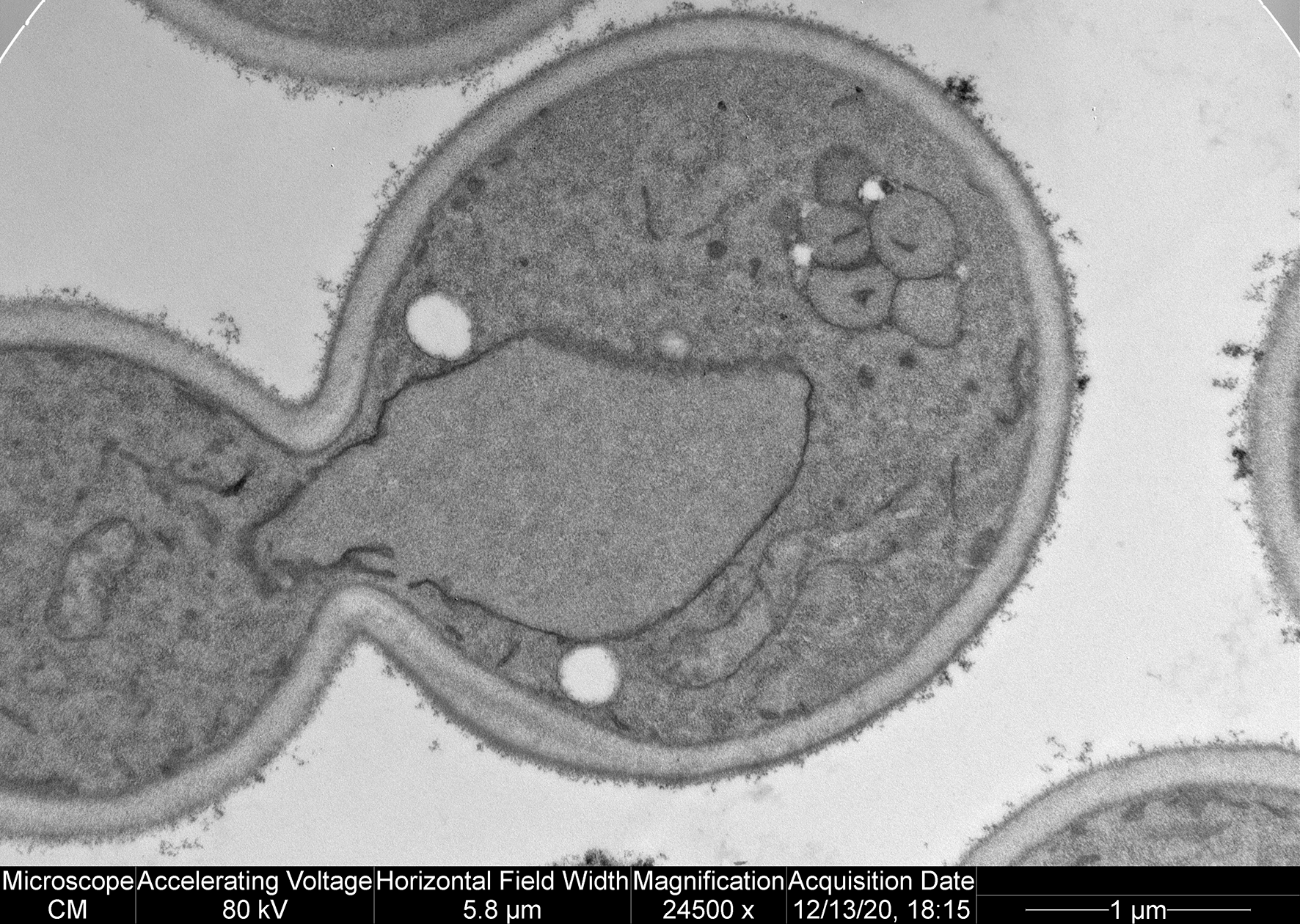

Supplement: Supplementary file 4 — Source Data Fig. 3 [file 44319_2023_55_MOESM4_ESM.zip › Figure 3/3D/EM_44A_Rich_HU.tif]

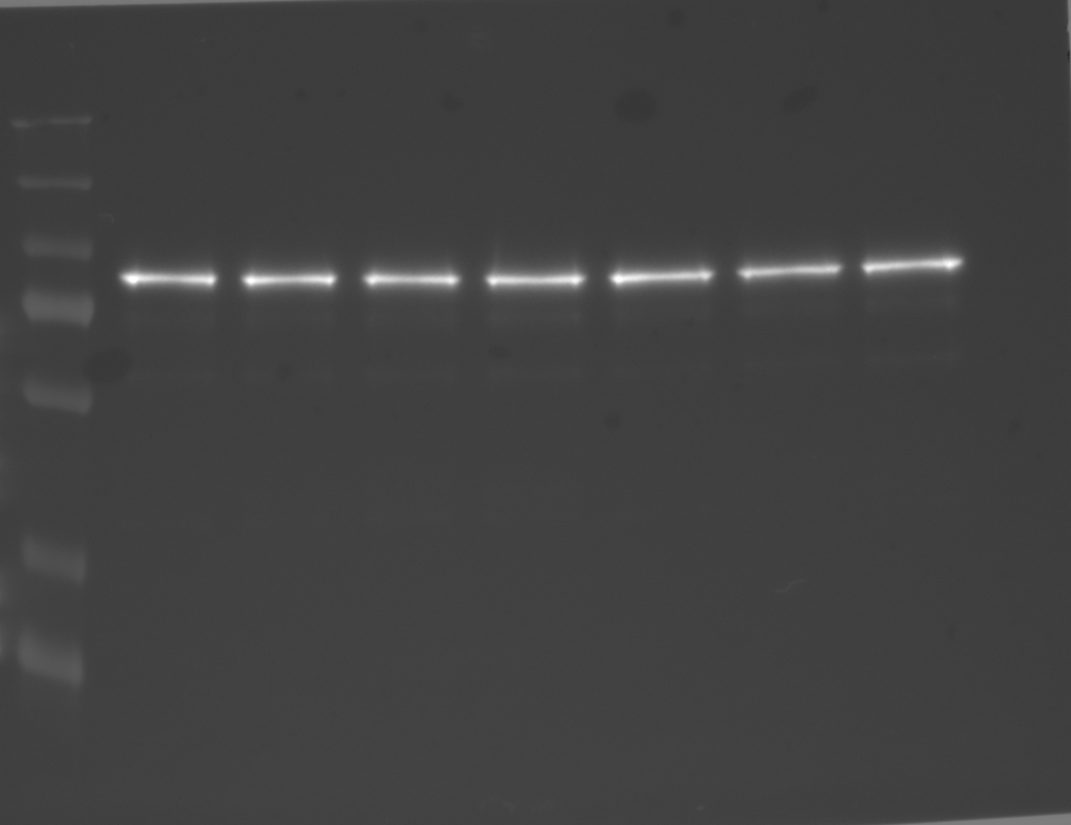

Supplement: Supplementary file 5 — Source Data Fig. 4 [file 44319_2023_55_MOESM5_ESM.zip › Figure 4/4C/Input_Atg17.Tif]

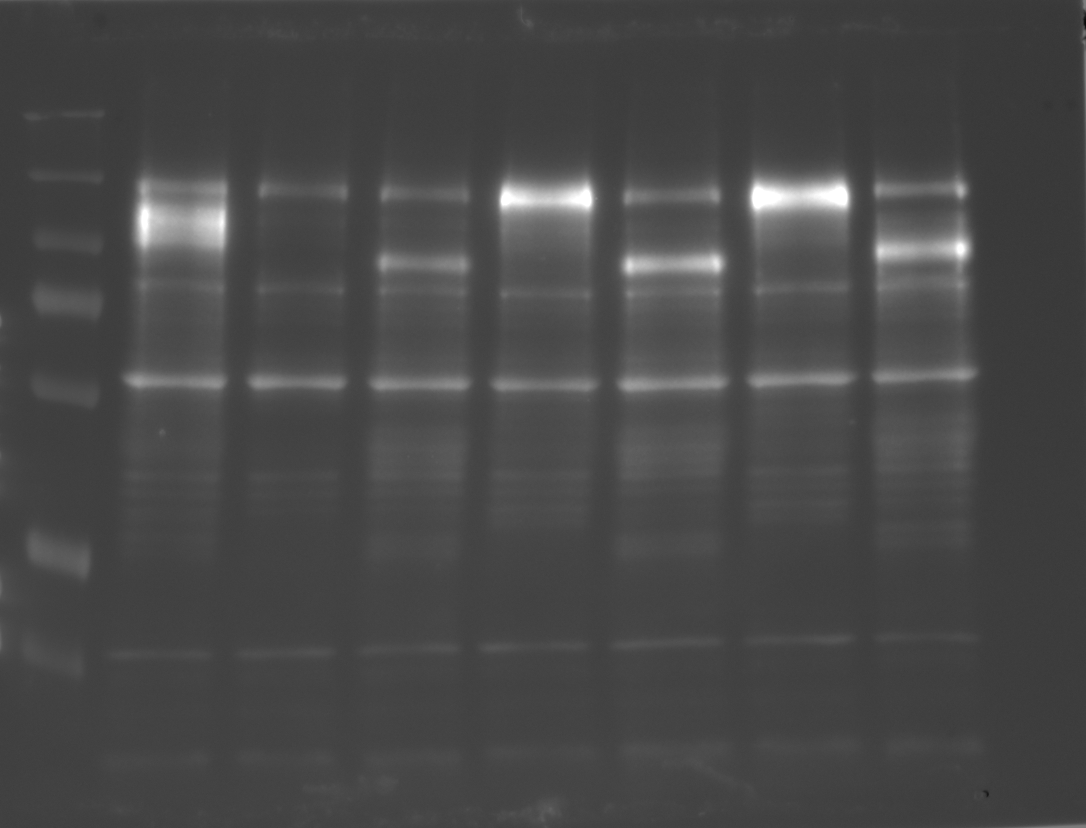

Supplement: Supplementary file 5 — Source Data Fig. 4 [file 44319_2023_55_MOESM5_ESM.zip › Figure 4/4C/Input_Atg13.Tif]

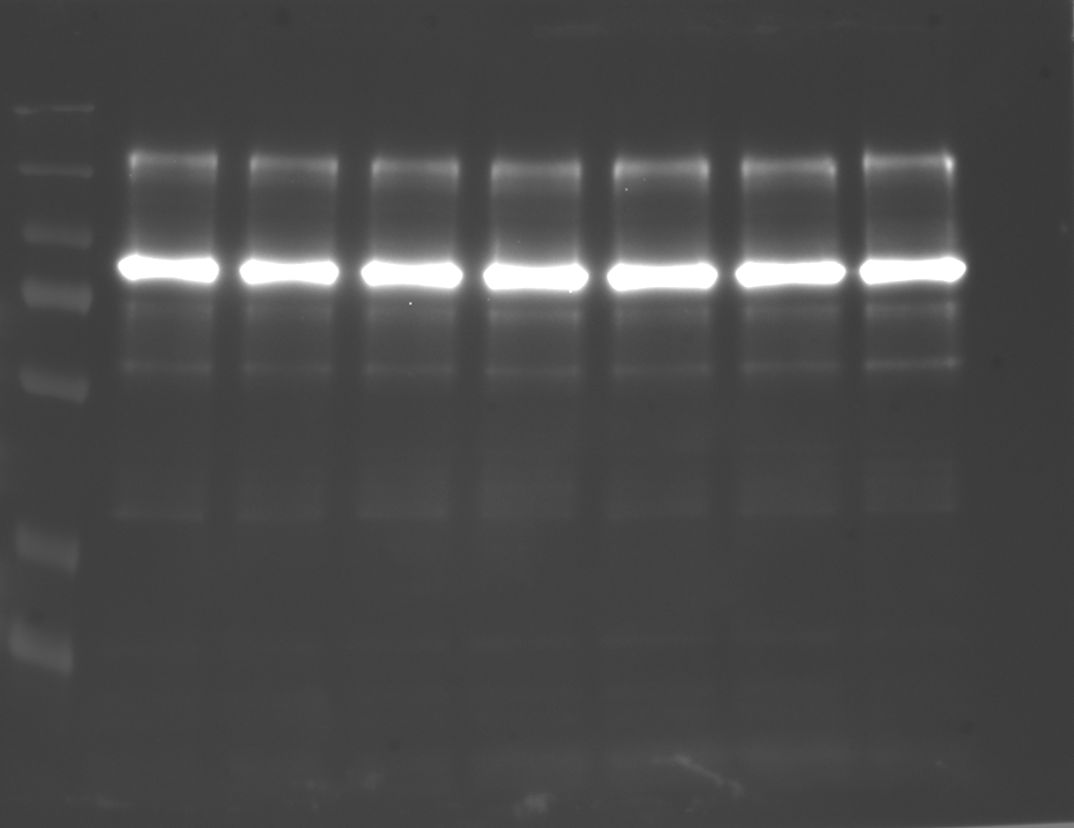

Supplement: Supplementary file 5 — Source Data Fig. 4 [file 44319_2023_55_MOESM5_ESM.zip › Figure 4/4C/Input_Atg11.Tif]

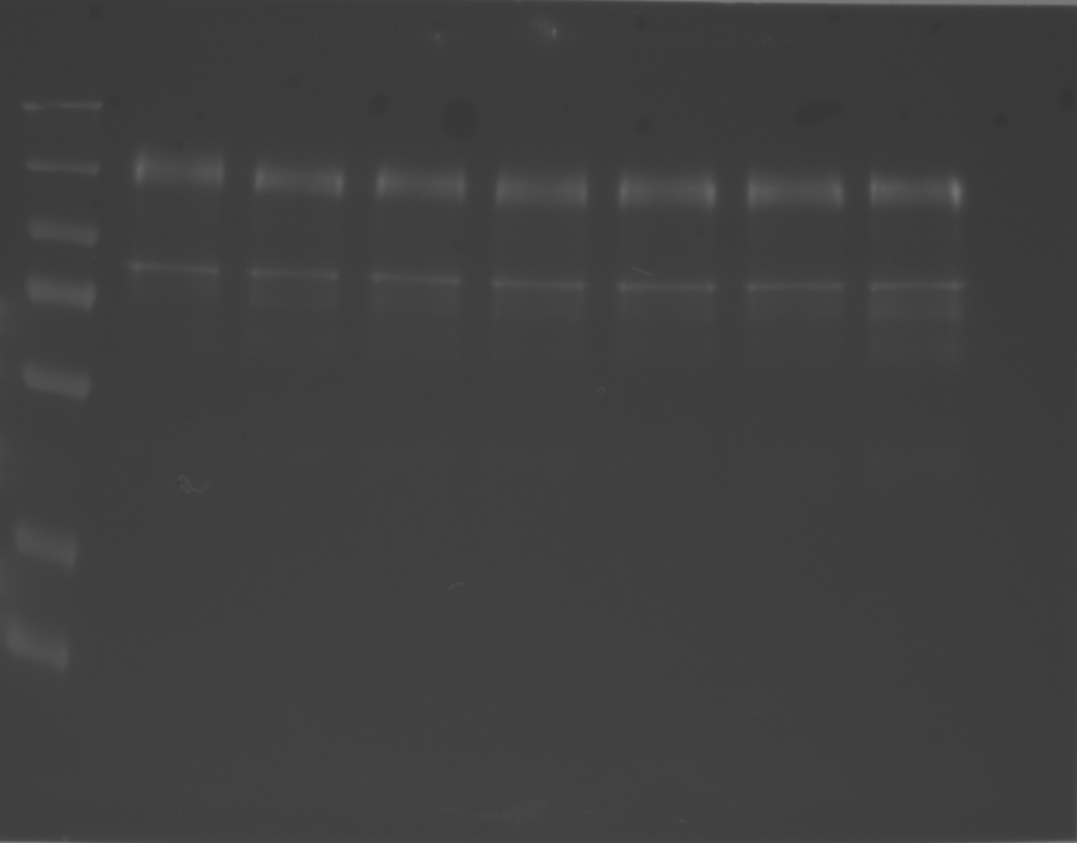

Supplement: Supplementary file 5 — Source Data Fig. 4 [file 44319_2023_55_MOESM5_ESM.zip › Figure 4/4C/Input_Atg1.Tif]

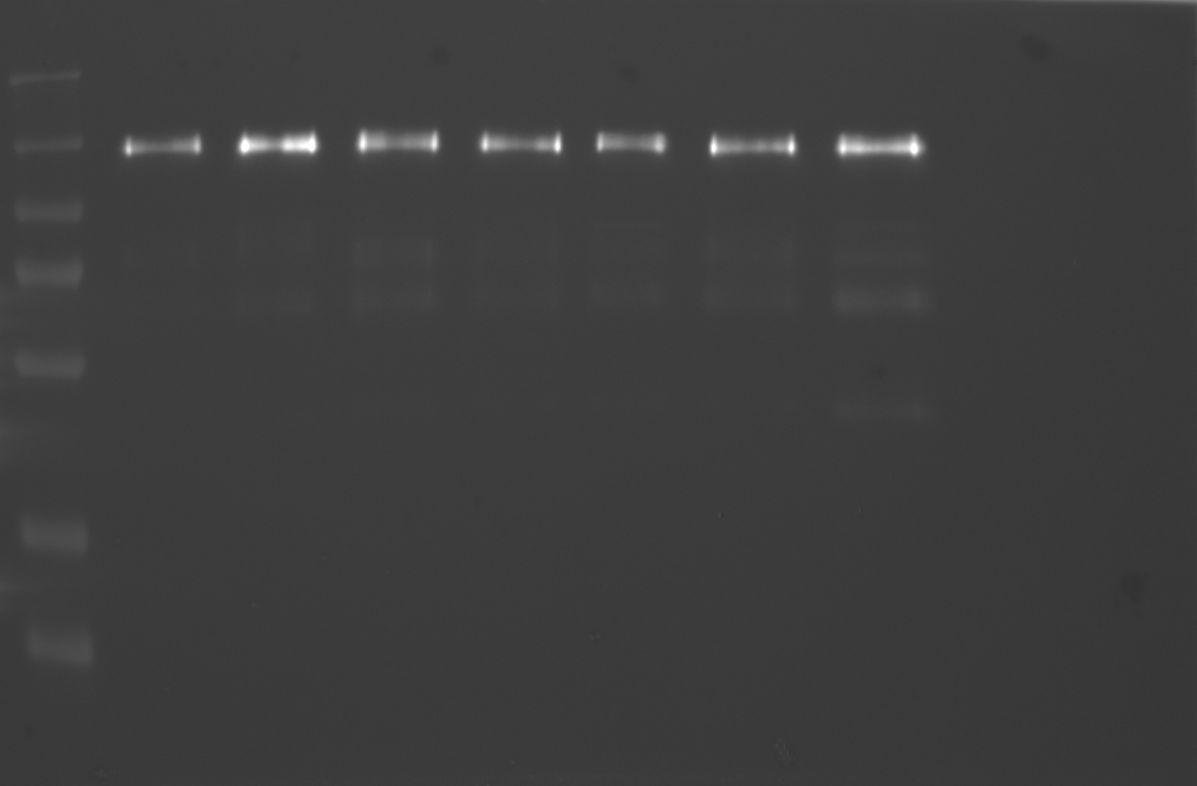

Supplement: Supplementary file 5 — Source Data Fig. 4 [file 44319_2023_55_MOESM5_ESM.zip › Figure 4/4C/IP_Atg1.Tif]

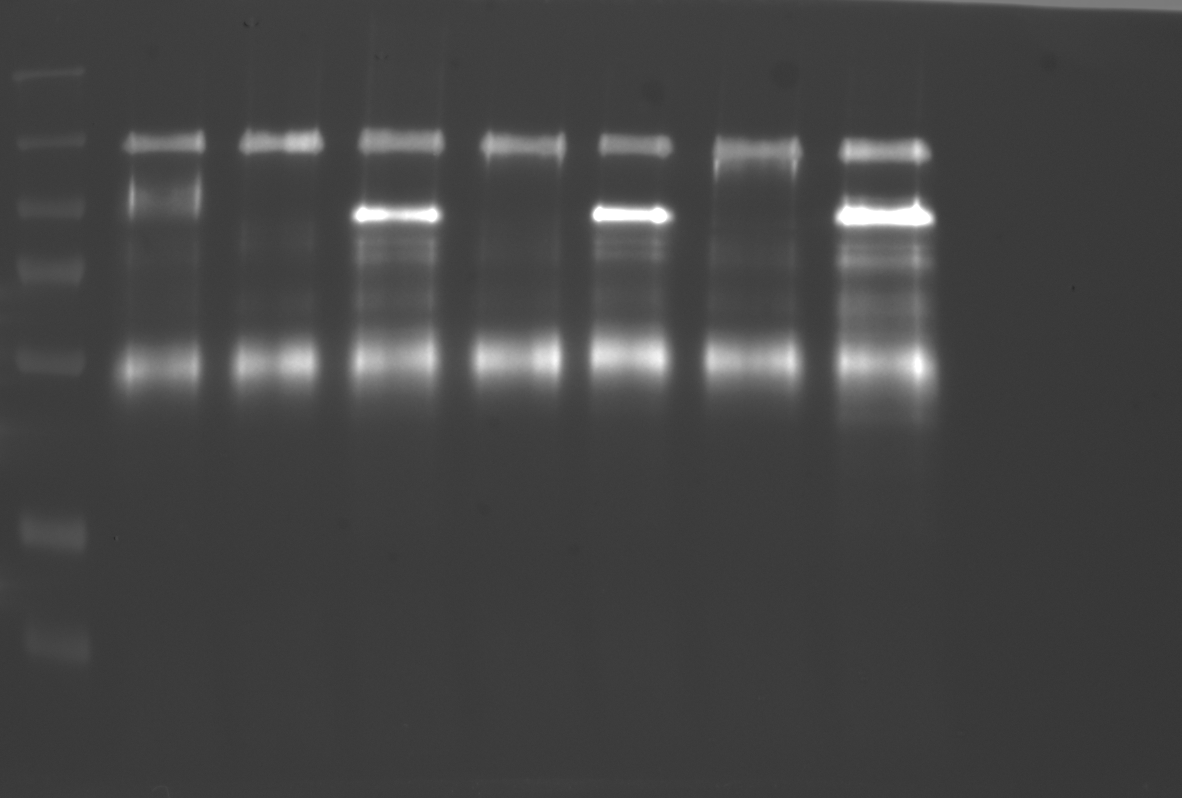

Supplement: Supplementary file 5 — Source Data Fig. 4 [file 44319_2023_55_MOESM5_ESM.zip › Figure 4/4C/IP_Atg13.Tif]

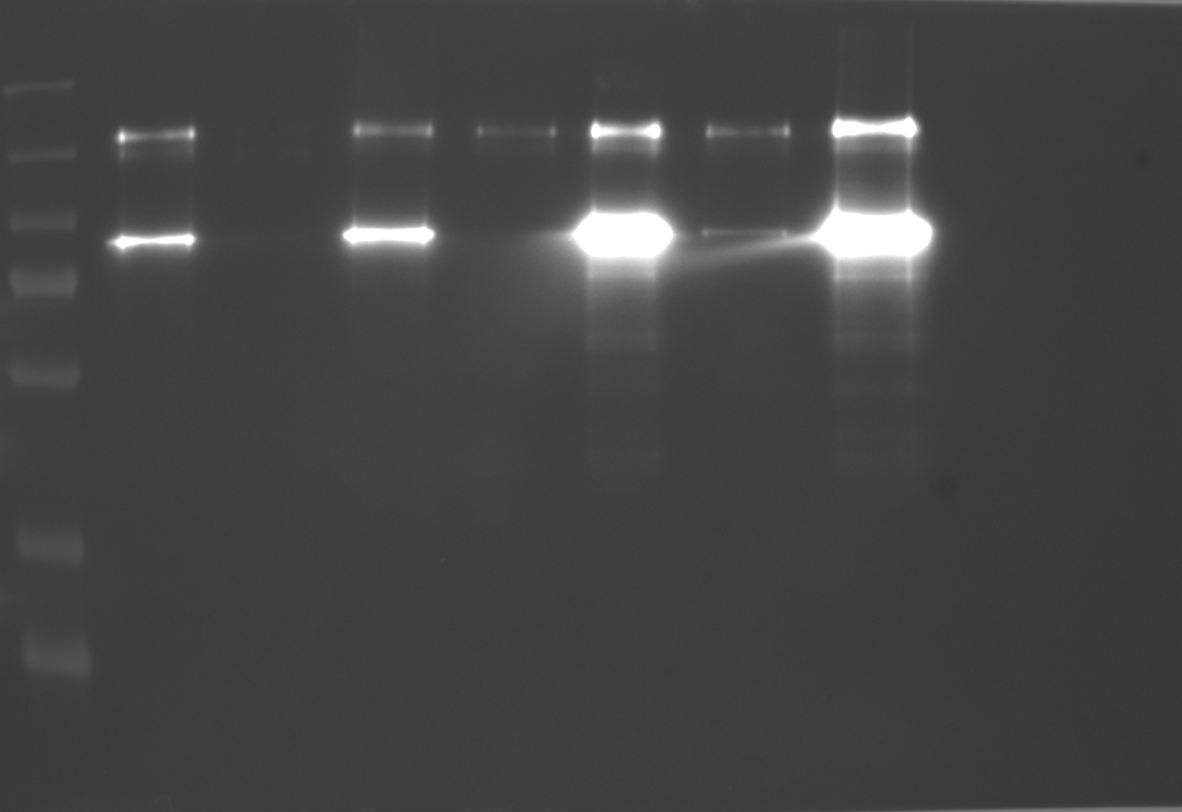

Supplement: Supplementary file 5 — Source Data Fig. 4 [file 44319_2023_55_MOESM5_ESM.zip › Figure 4/4C/IP_Atg11.Tif]

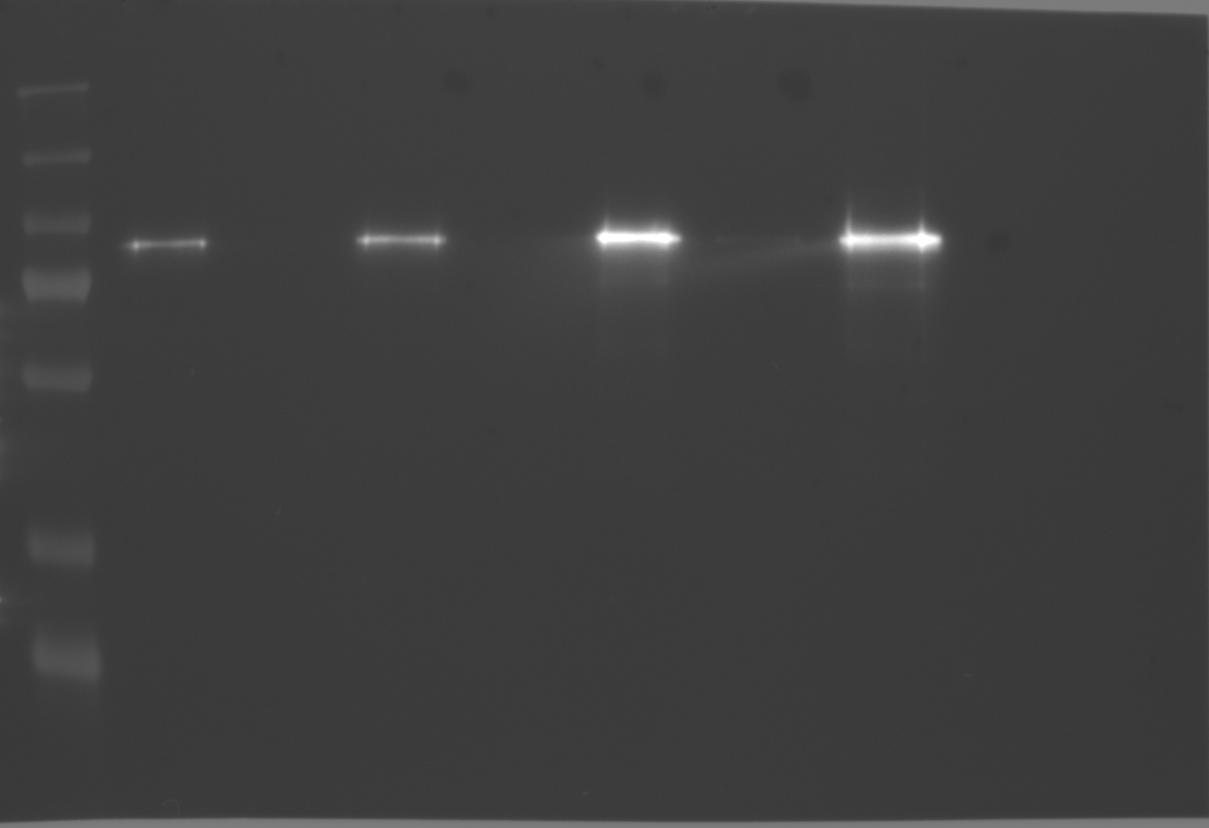

Supplement: Supplementary file 5 — Source Data Fig. 4 [file 44319_2023_55_MOESM5_ESM.zip › Figure 4/4C/IP_Atg17.Tif]

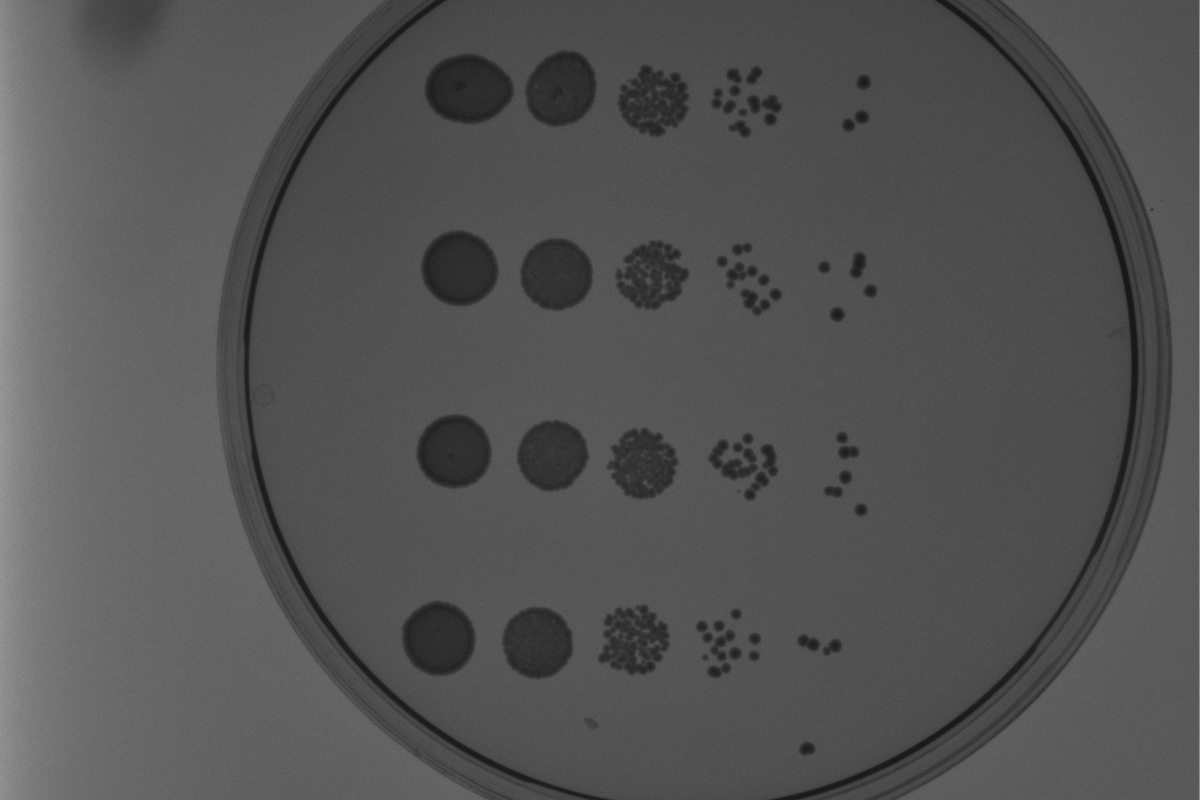

Supplement: Supplementary file 5 — Source Data Fig. 4 [file 44319_2023_55_MOESM5_ESM.zip › Figure 4/4D/Spotting_assay_Day0_starvation.jpeg]

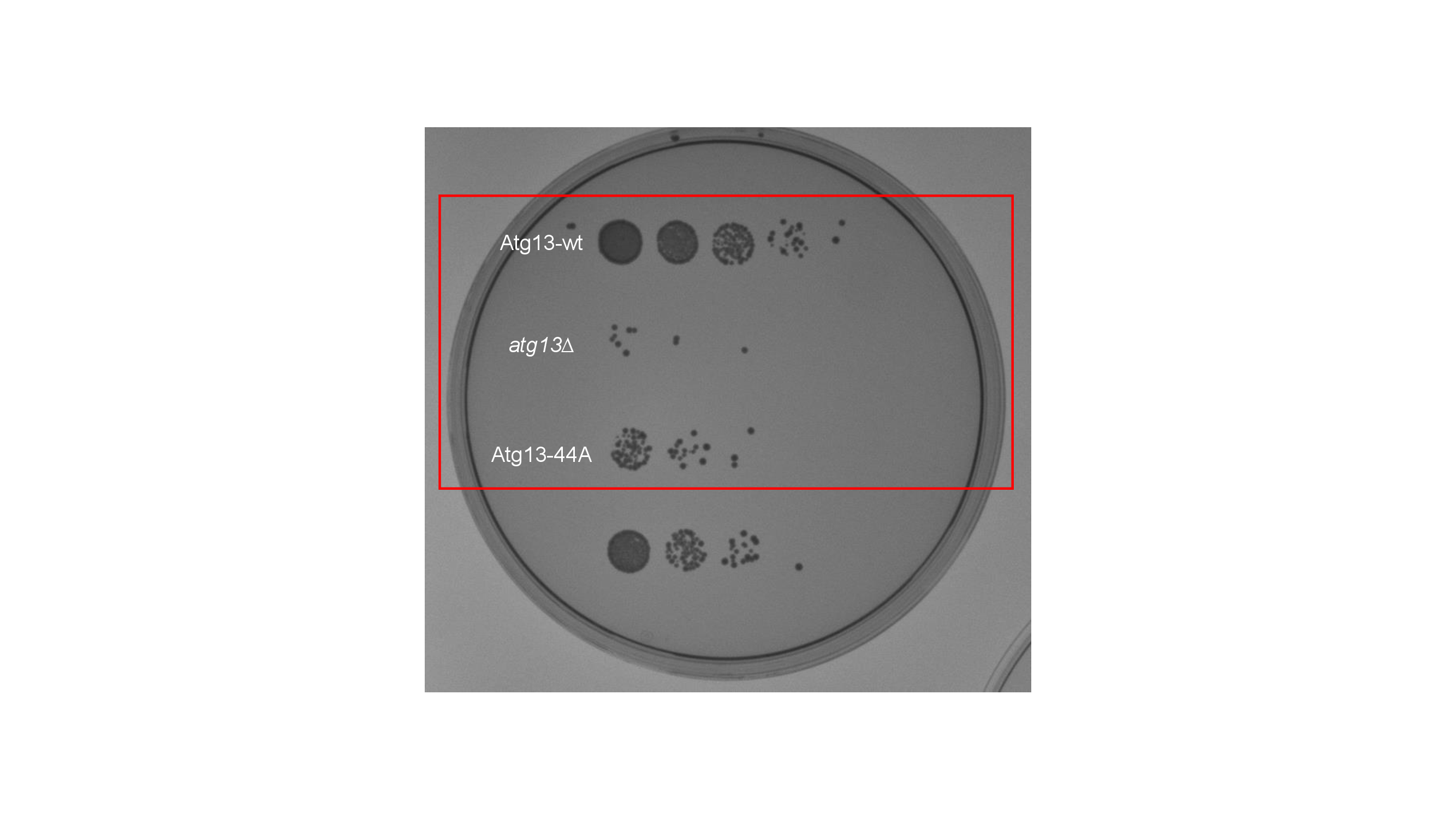

Supplement: Supplementary file 5 — Source Data Fig. 4 [file 44319_2023_55_MOESM5_ESM.zip › Figure 4/4D/Spotting_assay_Day14_starvation_marked.tiff]

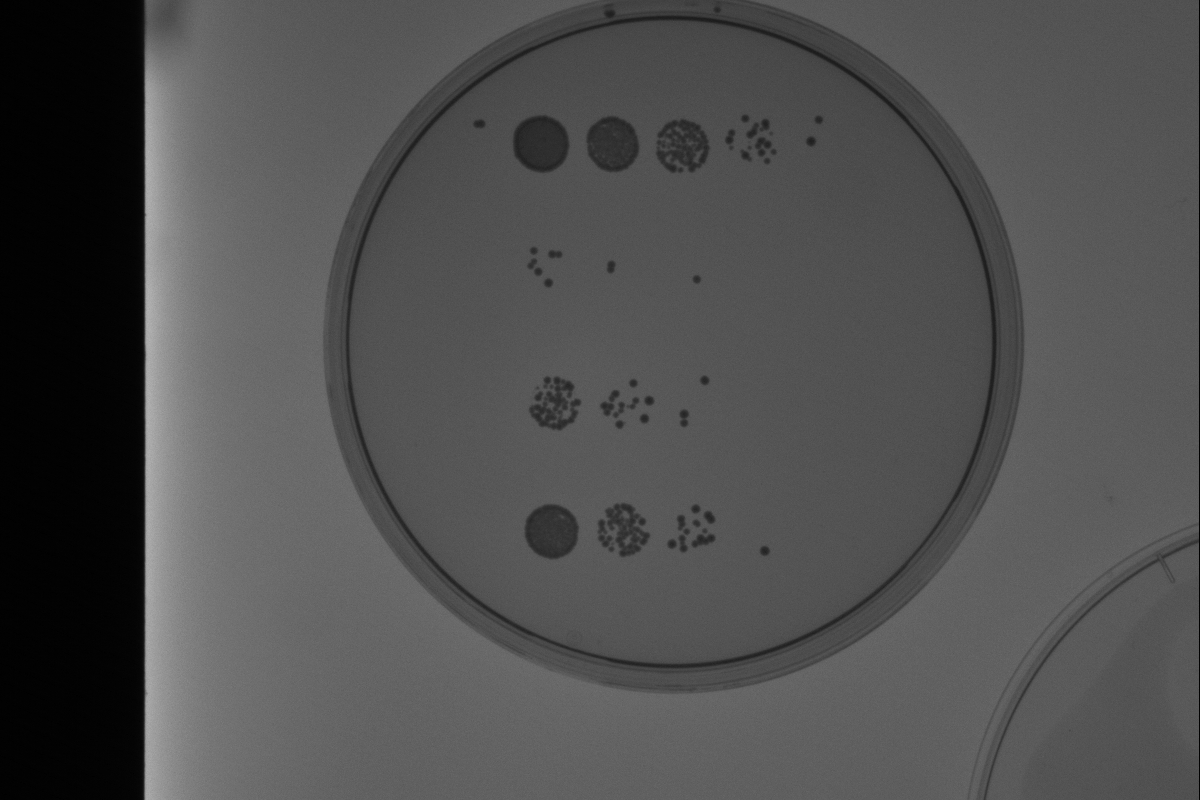

Supplement: Supplementary file 5 — Source Data Fig. 4 [file 44319_2023_55_MOESM5_ESM.zip › Figure 4/4D/Spotting_assay_Day14_starvation.tiff]

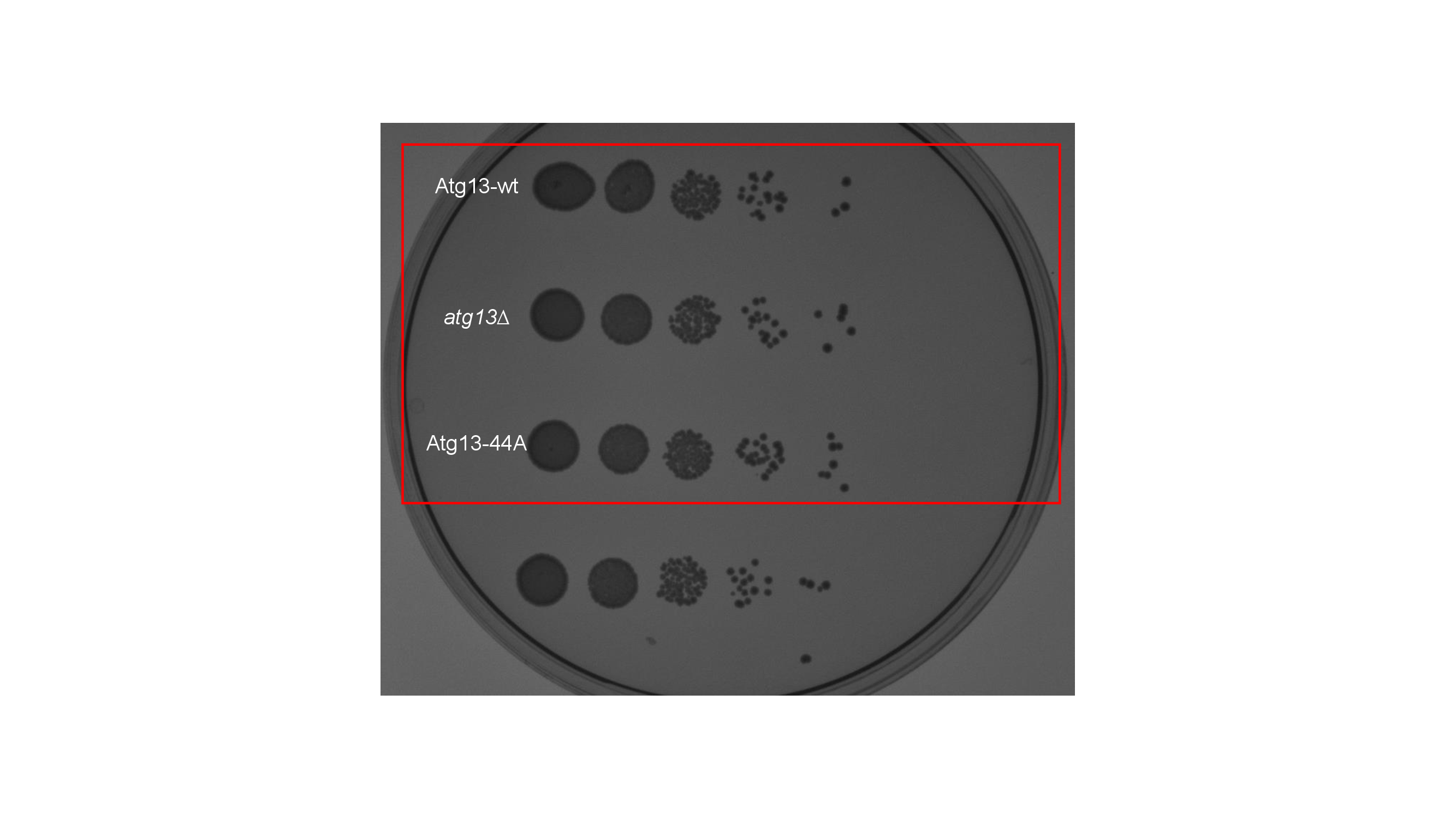

Supplement: Supplementary file 5 — Source Data Fig. 4 [file 44319_2023_55_MOESM5_ESM.zip › Figure 4/4D/Spotting_assay_Day0_starvation_marked.tiff]

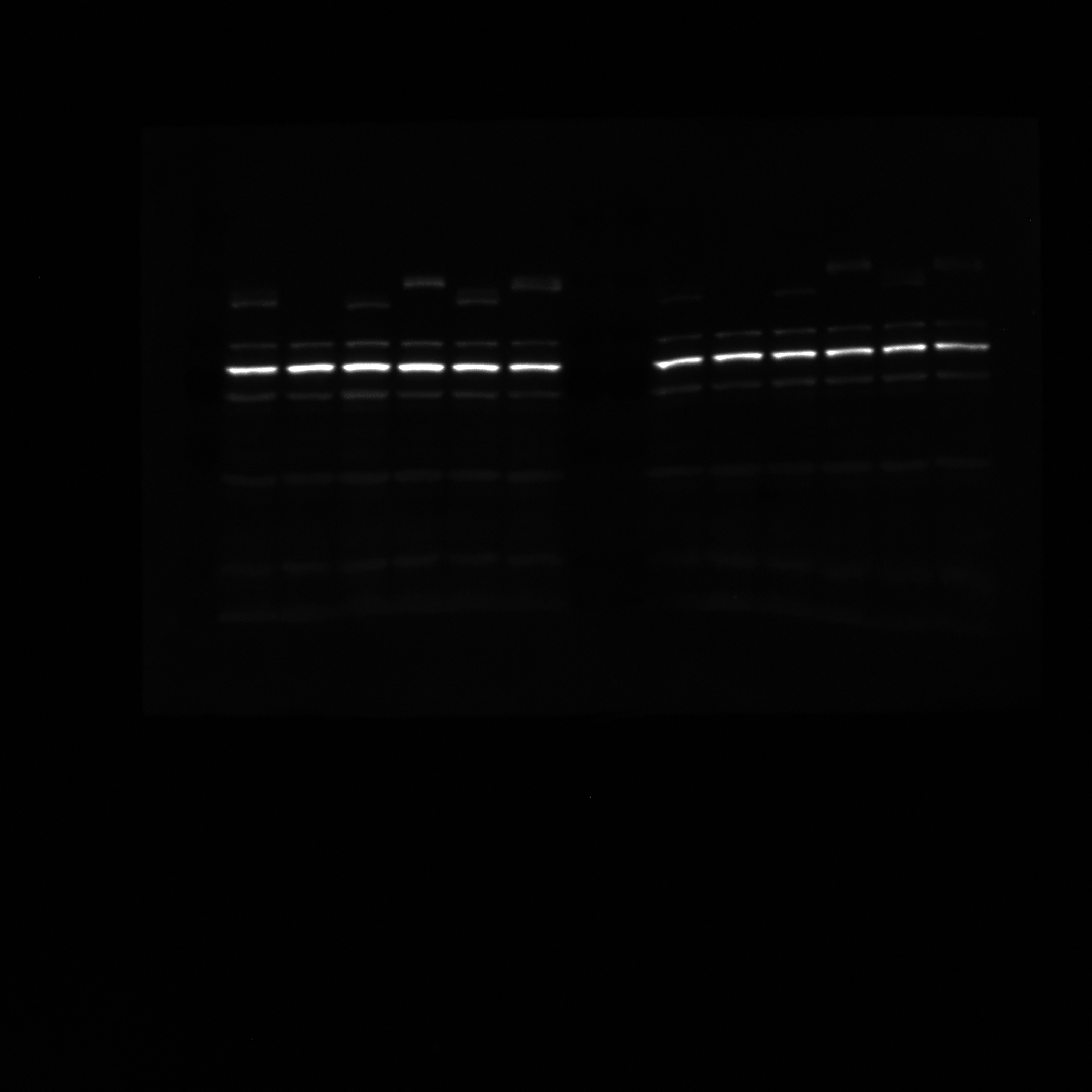

Supplement: Supplementary file 6 — Source Data Fig. 5 [file 44319_2023_55_MOESM6_ESM.zip › Figure 5/5C/Input_Atg17.Tif]

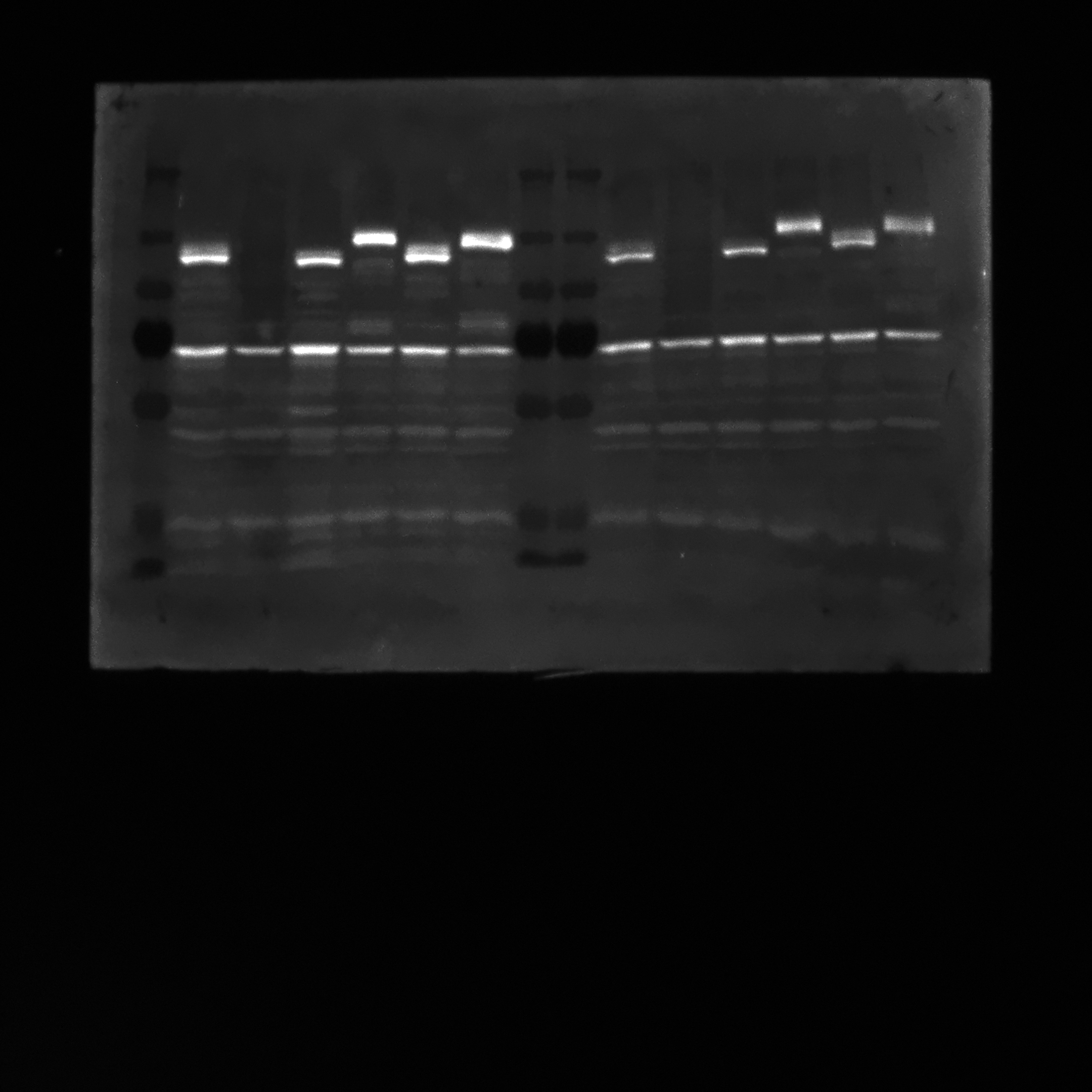

Supplement: Supplementary file 6 — Source Data Fig. 5 [file 44319_2023_55_MOESM6_ESM.zip › Figure 5/5C/Input_Atg13.Tif]

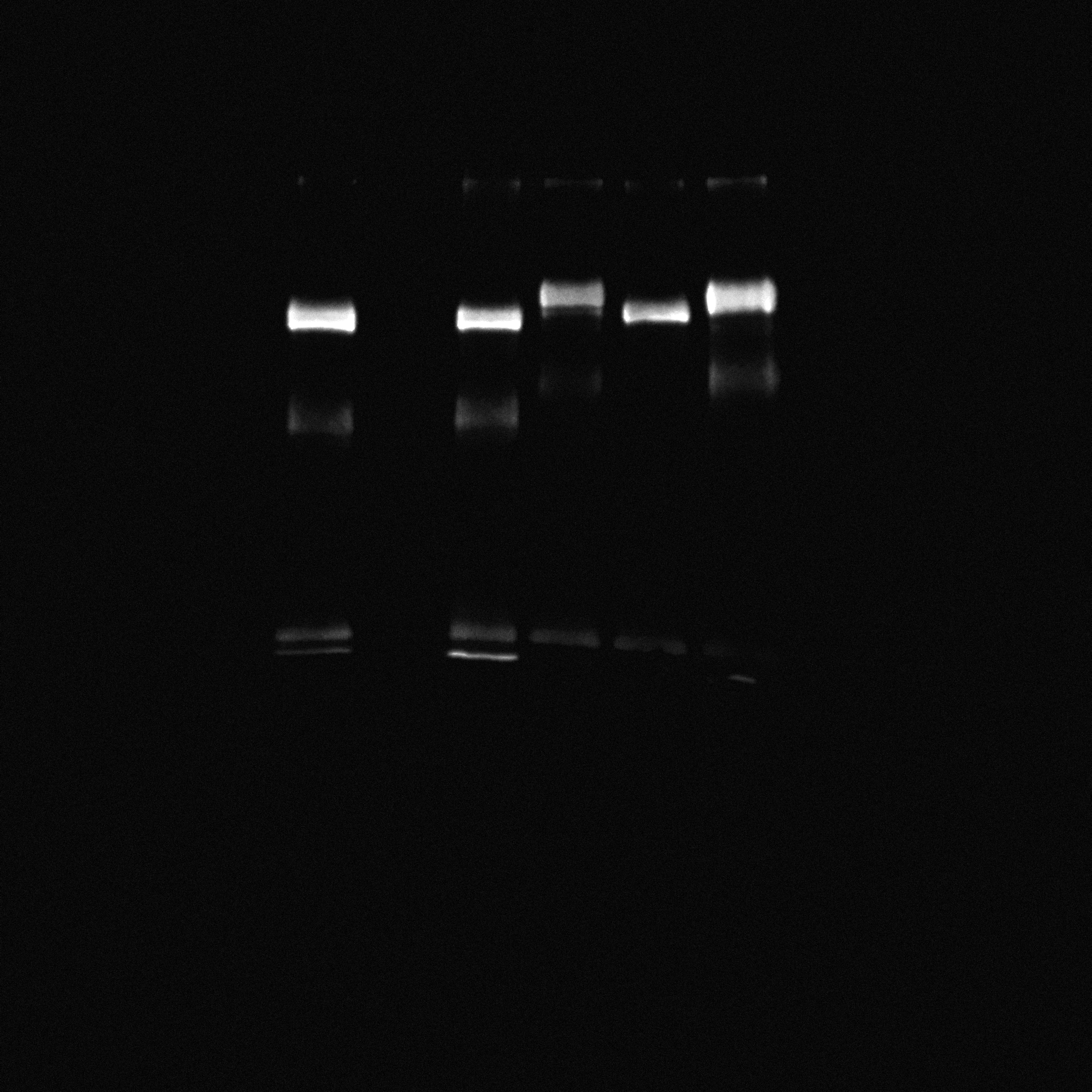

Supplement: Supplementary file 6 — Source Data Fig. 5 [file 44319_2023_55_MOESM6_ESM.zip › Figure 5/5C/IP_Atg13.Tif]

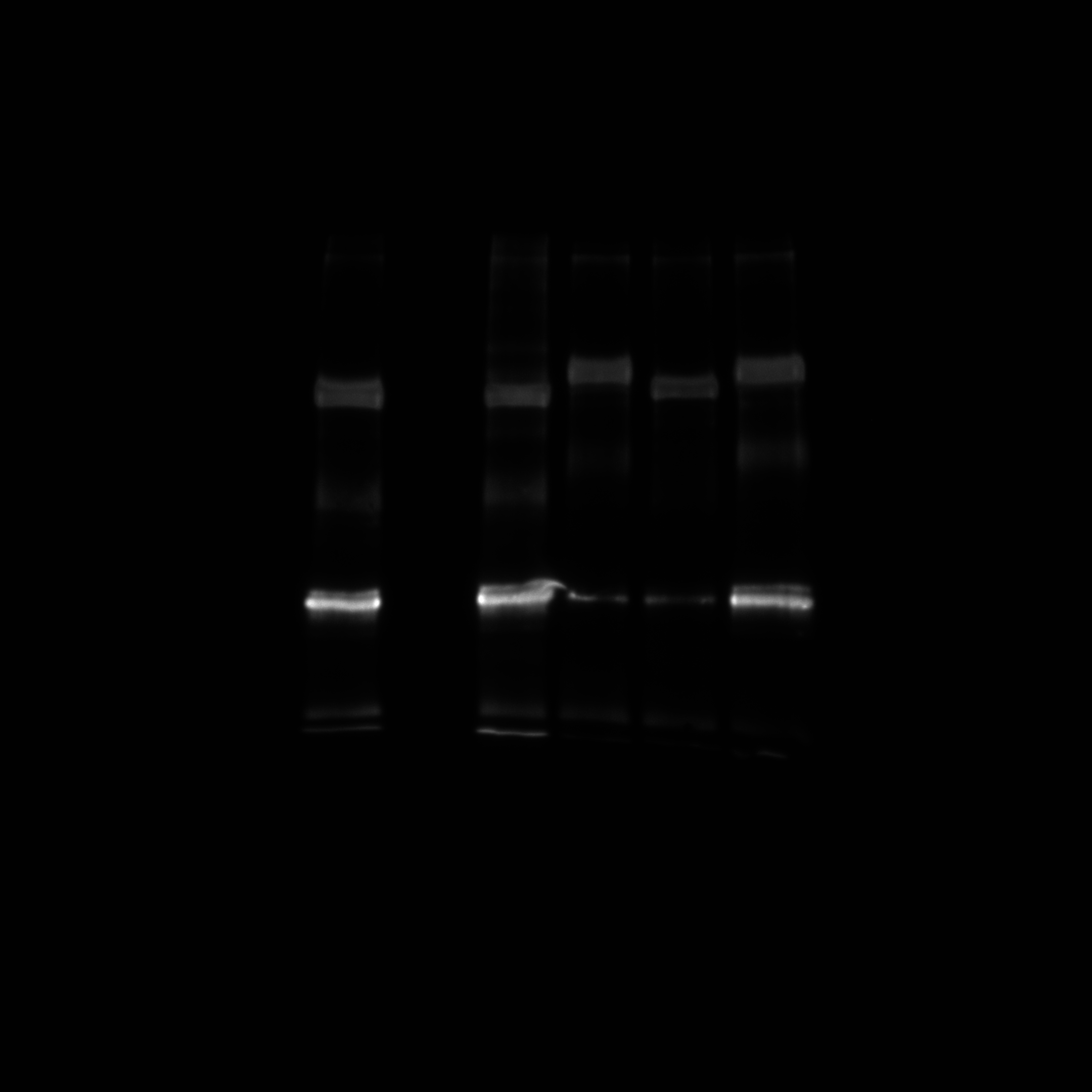

Supplement: Supplementary file 6 — Source Data Fig. 5 [file 44319_2023_55_MOESM6_ESM.zip › Figure 5/5C/IP_Atg17.Tif]

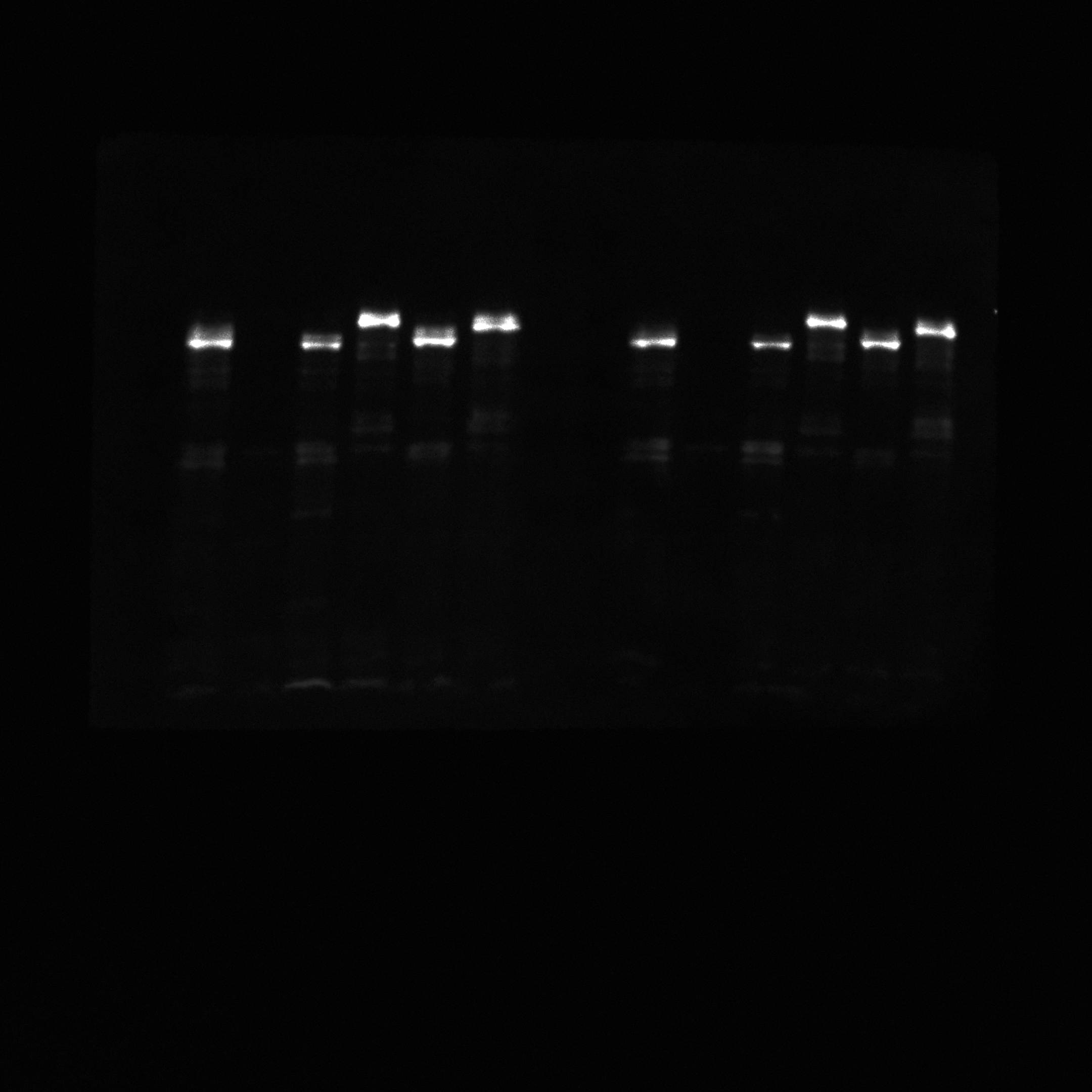

Supplement: Supplementary file 6 — Source Data Fig. 5 [file 44319_2023_55_MOESM6_ESM.zip › Figure 5/5D/Input_Atg13.Tif]

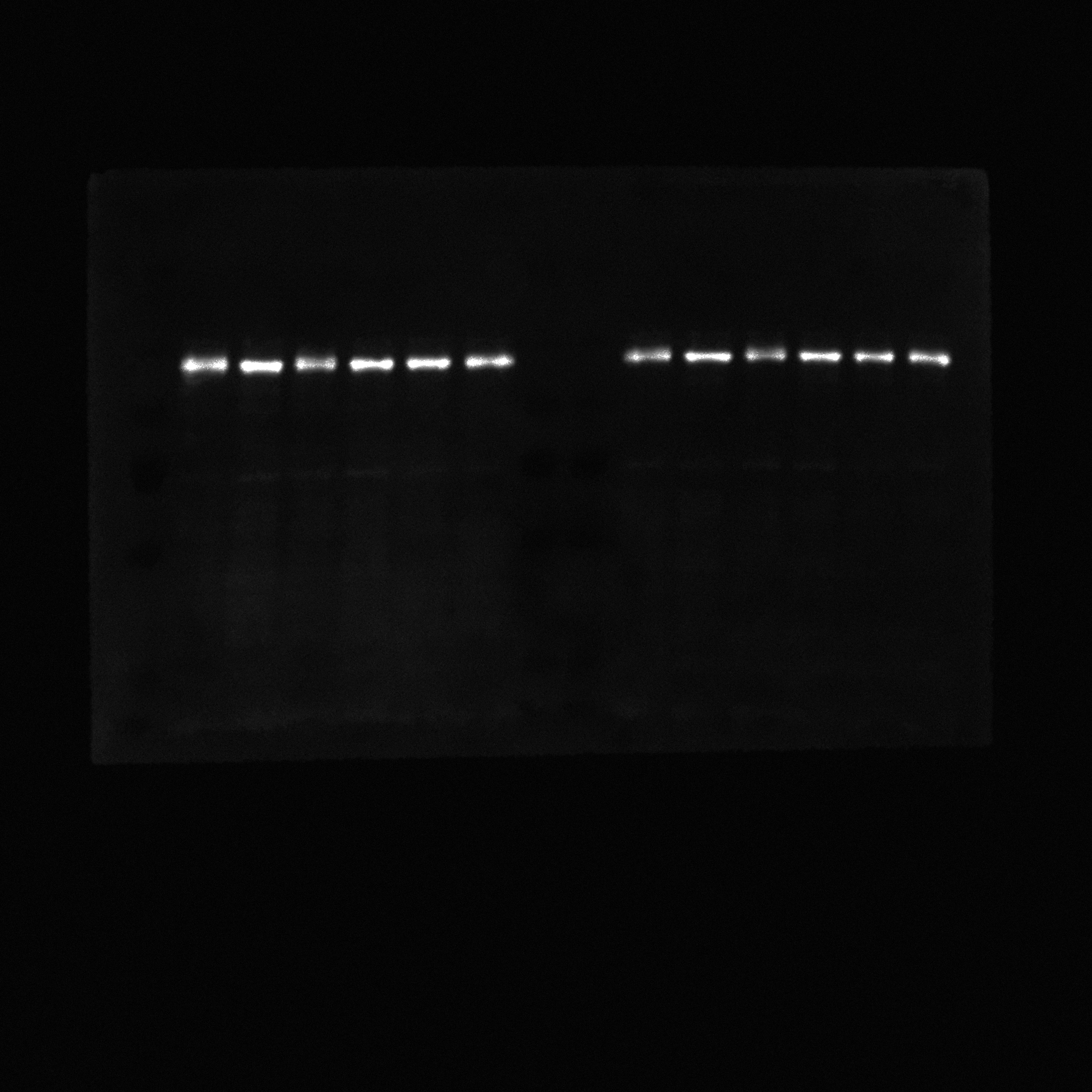

Supplement: Supplementary file 6 — Source Data Fig. 5 [file 44319_2023_55_MOESM6_ESM.zip › Figure 5/5D/Input_Atg1.Tif]

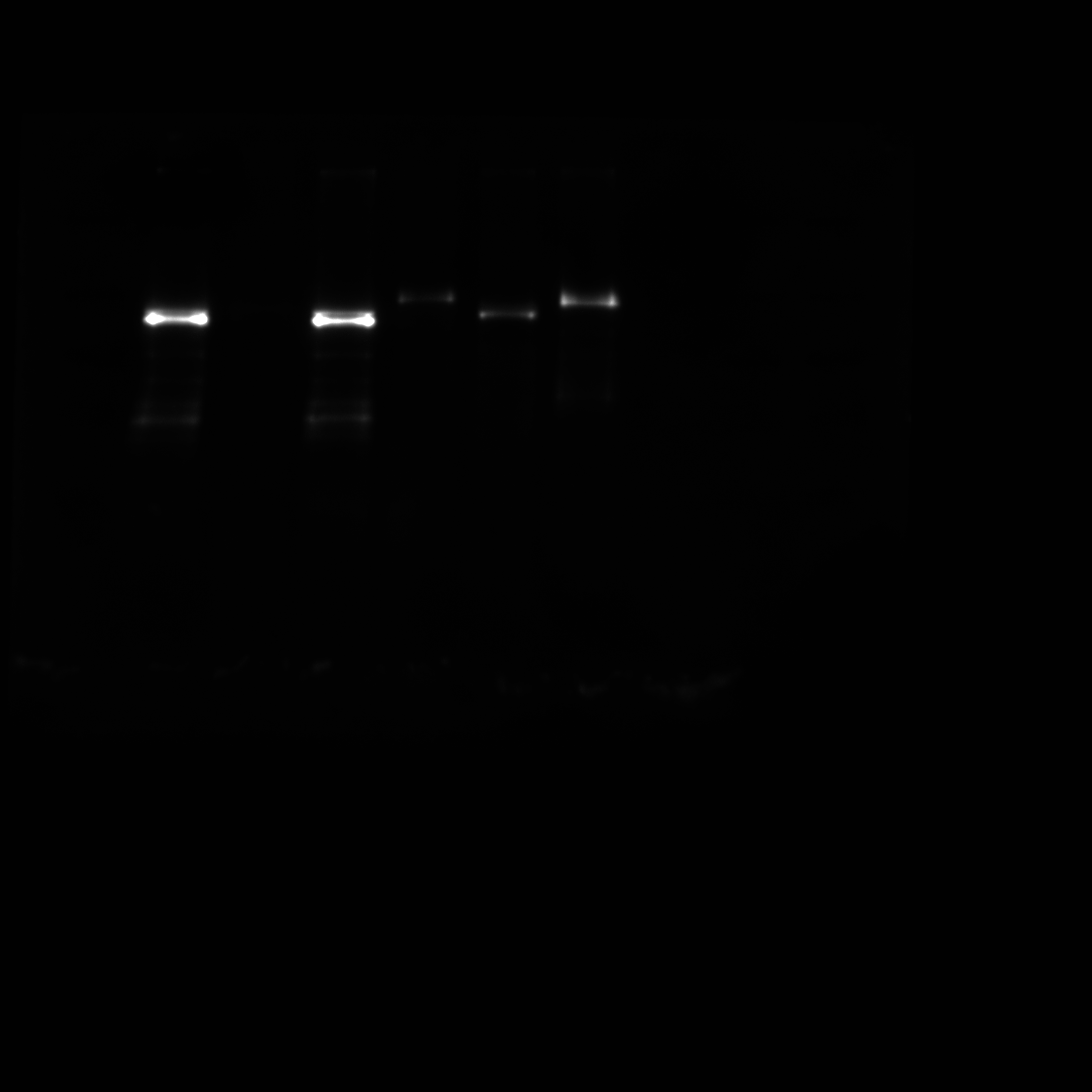

Supplement: Supplementary file 6 — Source Data Fig. 5 [file 44319_2023_55_MOESM6_ESM.zip › Figure 5/5D/IP_Atg13.Tif]

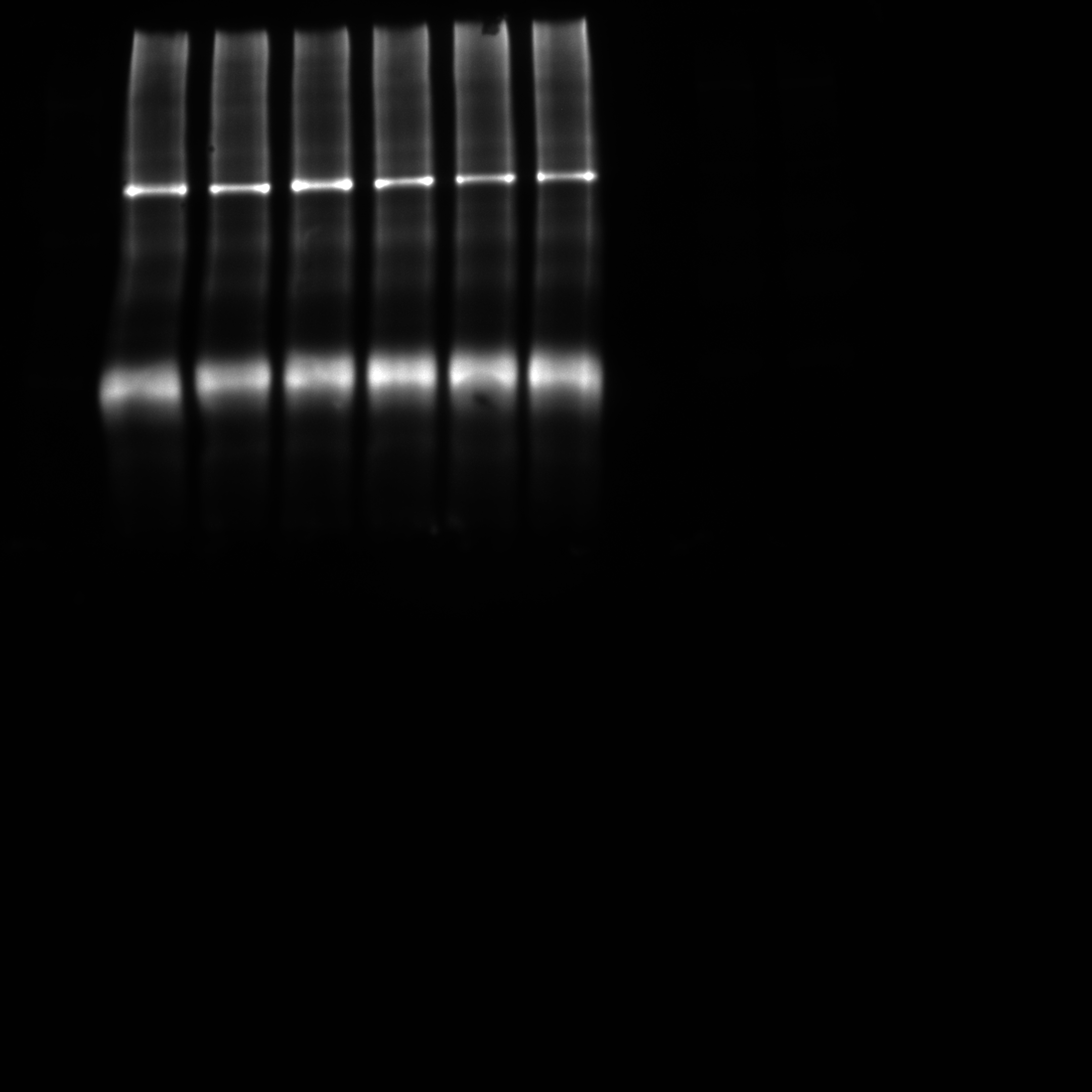

Supplement: Supplementary file 6 — Source Data Fig. 5 [file 44319_2023_55_MOESM6_ESM.zip › Figure 5/5D/IP_Atg1-protA.Tif]

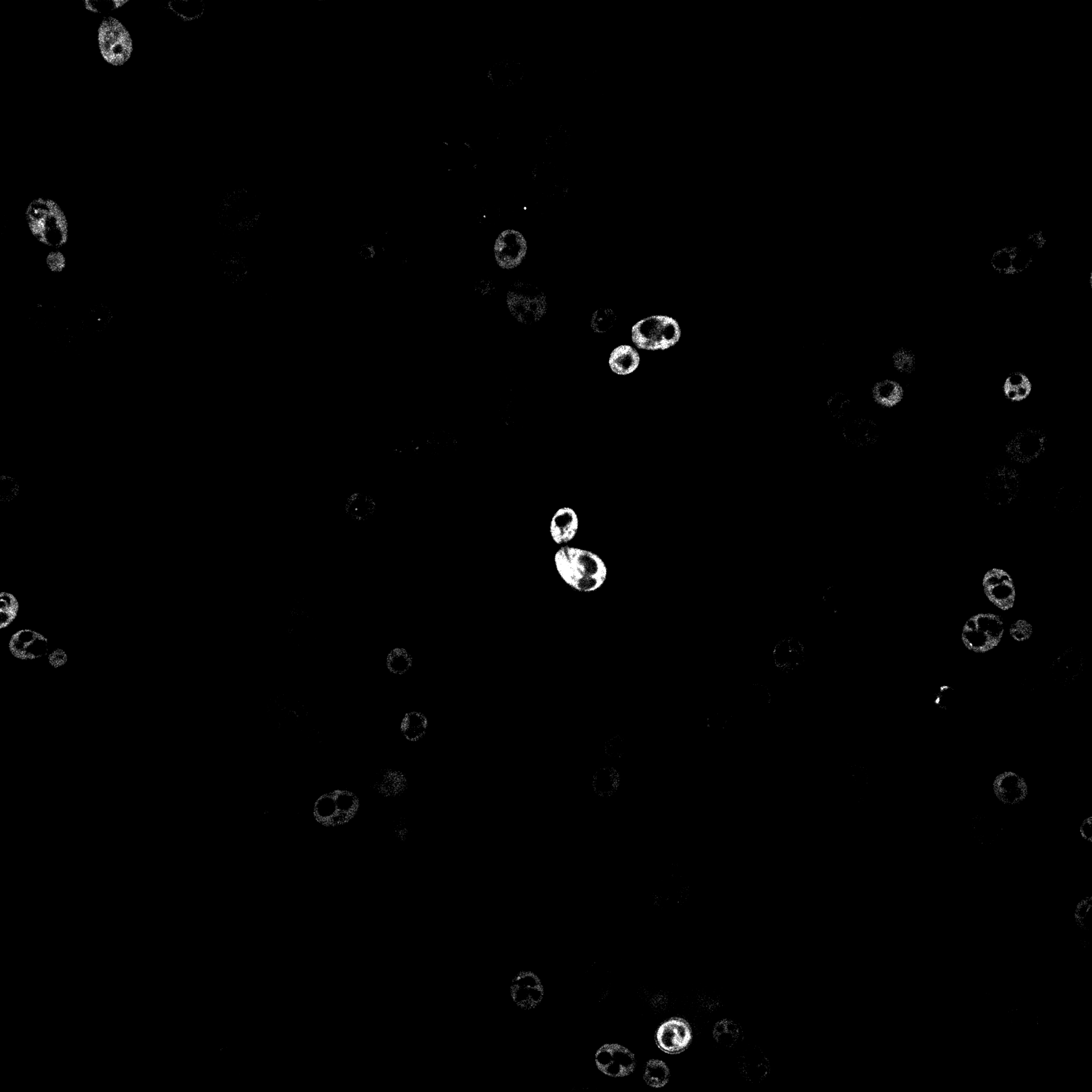

Supplement: Supplementary file 6 — Source Data Fig. 5 [file 44319_2023_55_MOESM6_ESM.zip › Figure 5/5G/Microsocpy_1-13CD-GFP_8D13D19D/GFP.tif]

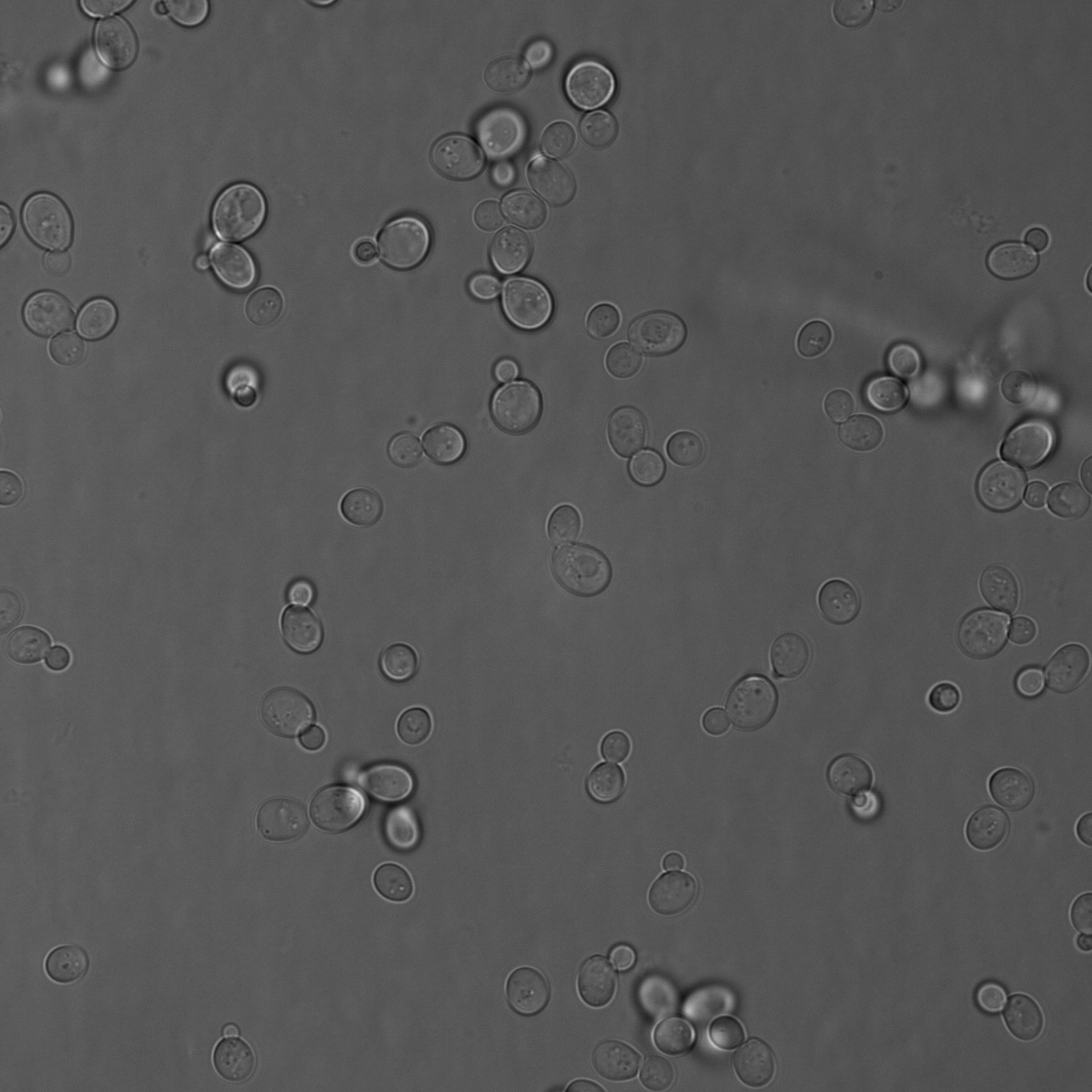

Supplement: Supplementary file 6 — Source Data Fig. 5 [file 44319_2023_55_MOESM6_ESM.zip › Figure 5/5G/Microsocpy_1-13CD-GFP_8D13D19D/BF.tif]

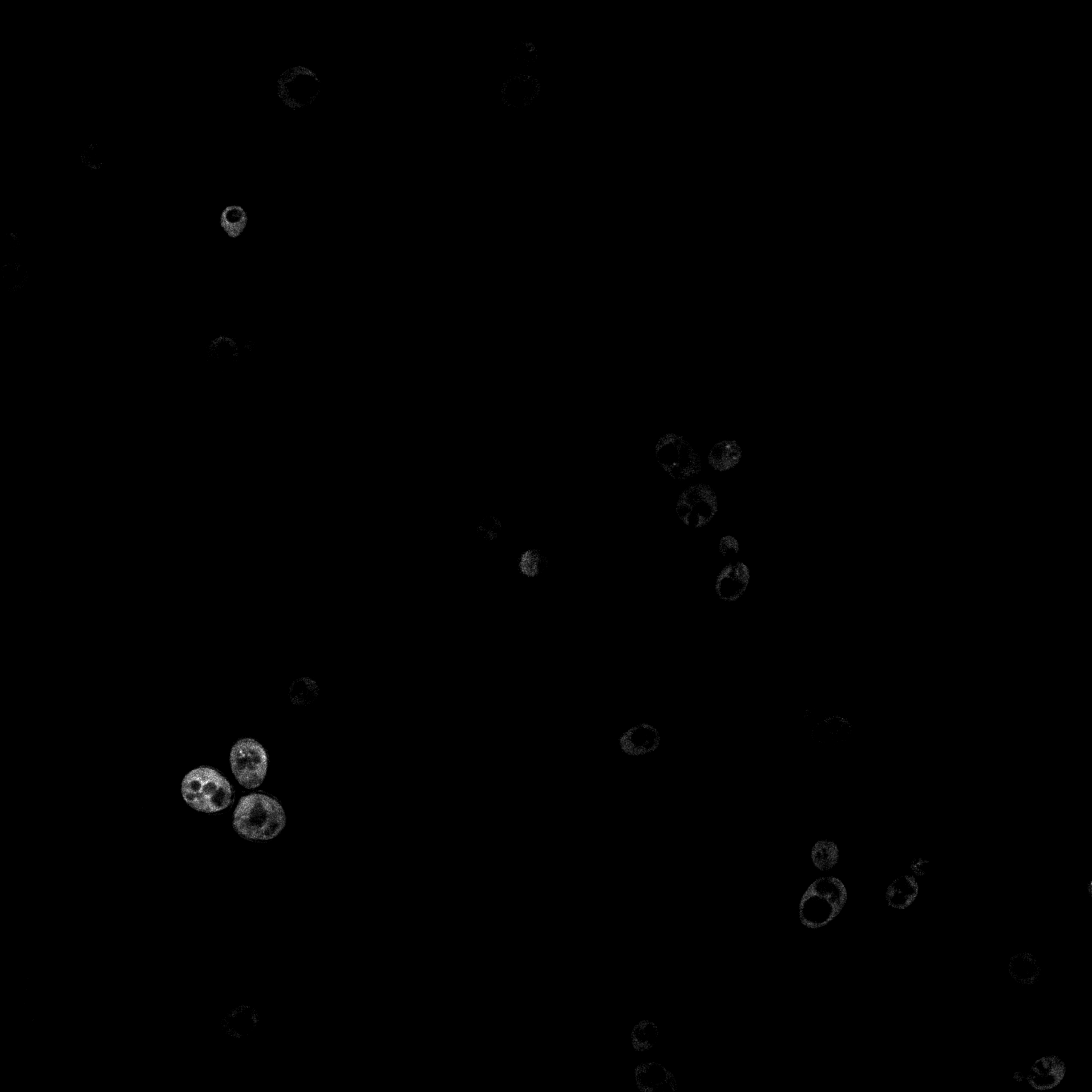

Supplement: Supplementary file 6 — Source Data Fig. 5 [file 44319_2023_55_MOESM6_ESM.zip › Figure 5/5G/Microsocpy_13CD-GFP_8D13D19D/GFP.tif]

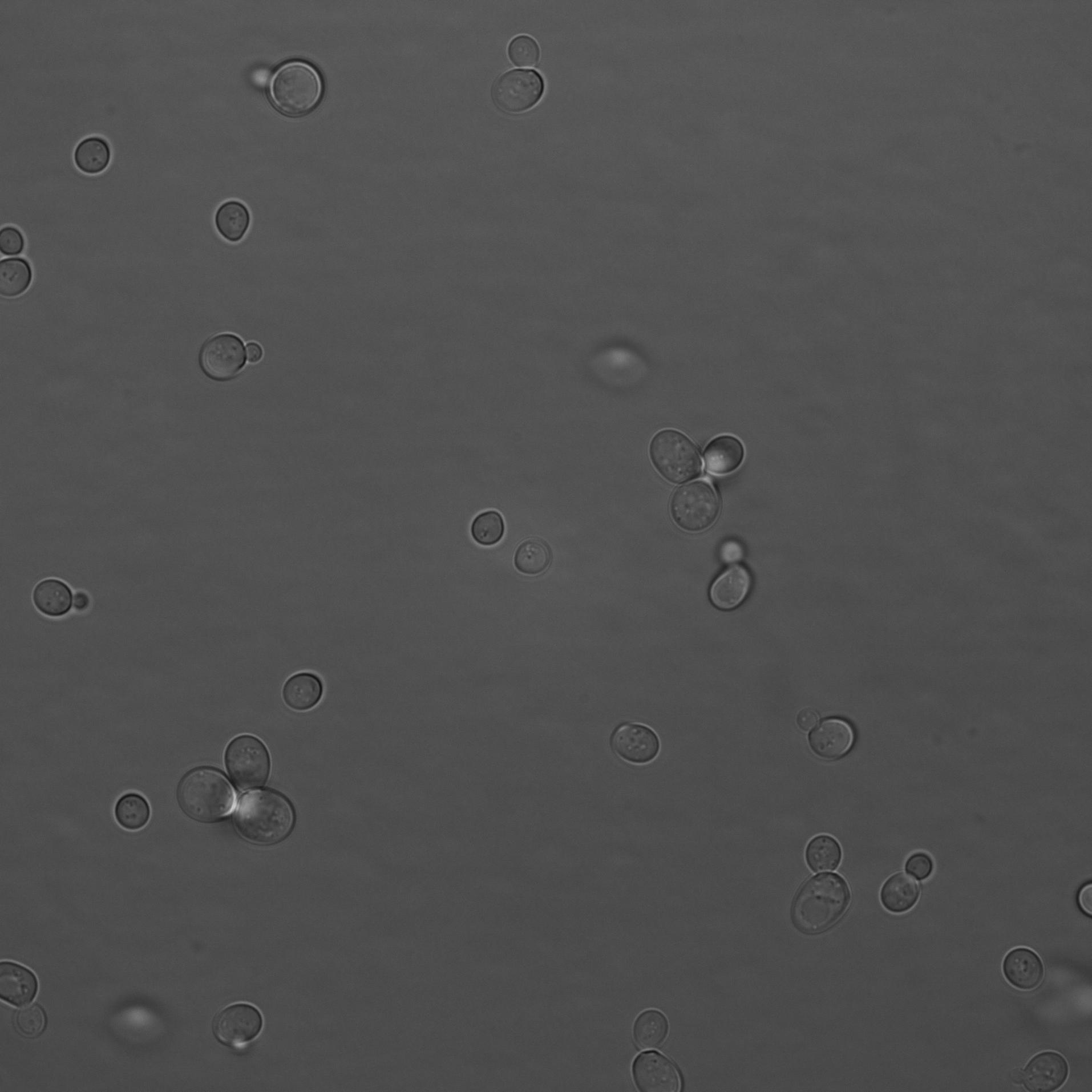

Supplement: Supplementary file 6 — Source Data Fig. 5 [file 44319_2023_55_MOESM6_ESM.zip › Figure 5/5G/Microsocpy_13CD-GFP_8D13D19D/BF.tif]

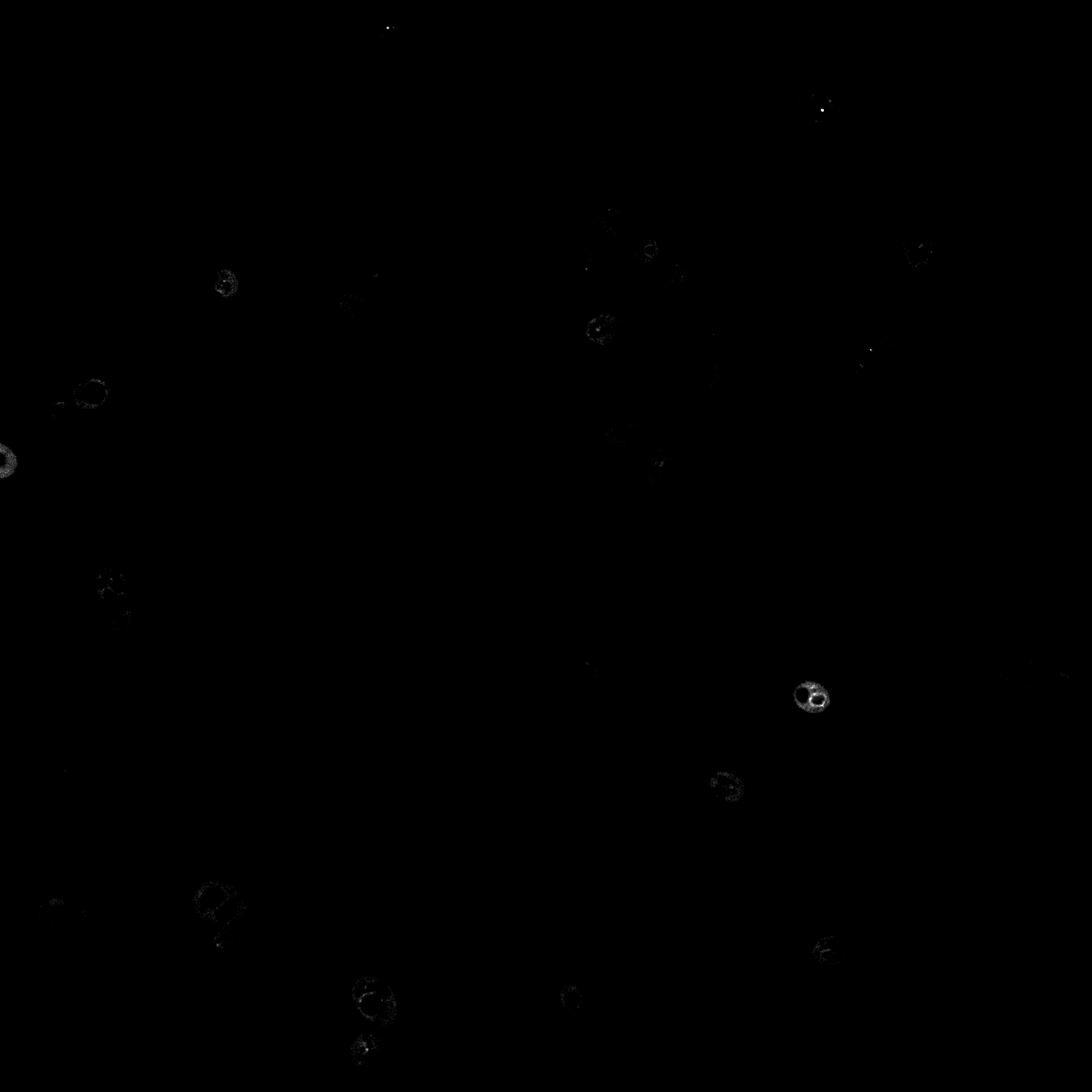

Supplement: Supplementary file 6 — Source Data Fig. 5 [file 44319_2023_55_MOESM6_ESM.zip › Figure 5/5G/Microsocpy_1-13wt-GFP_8D13D19D/GFP.tif]

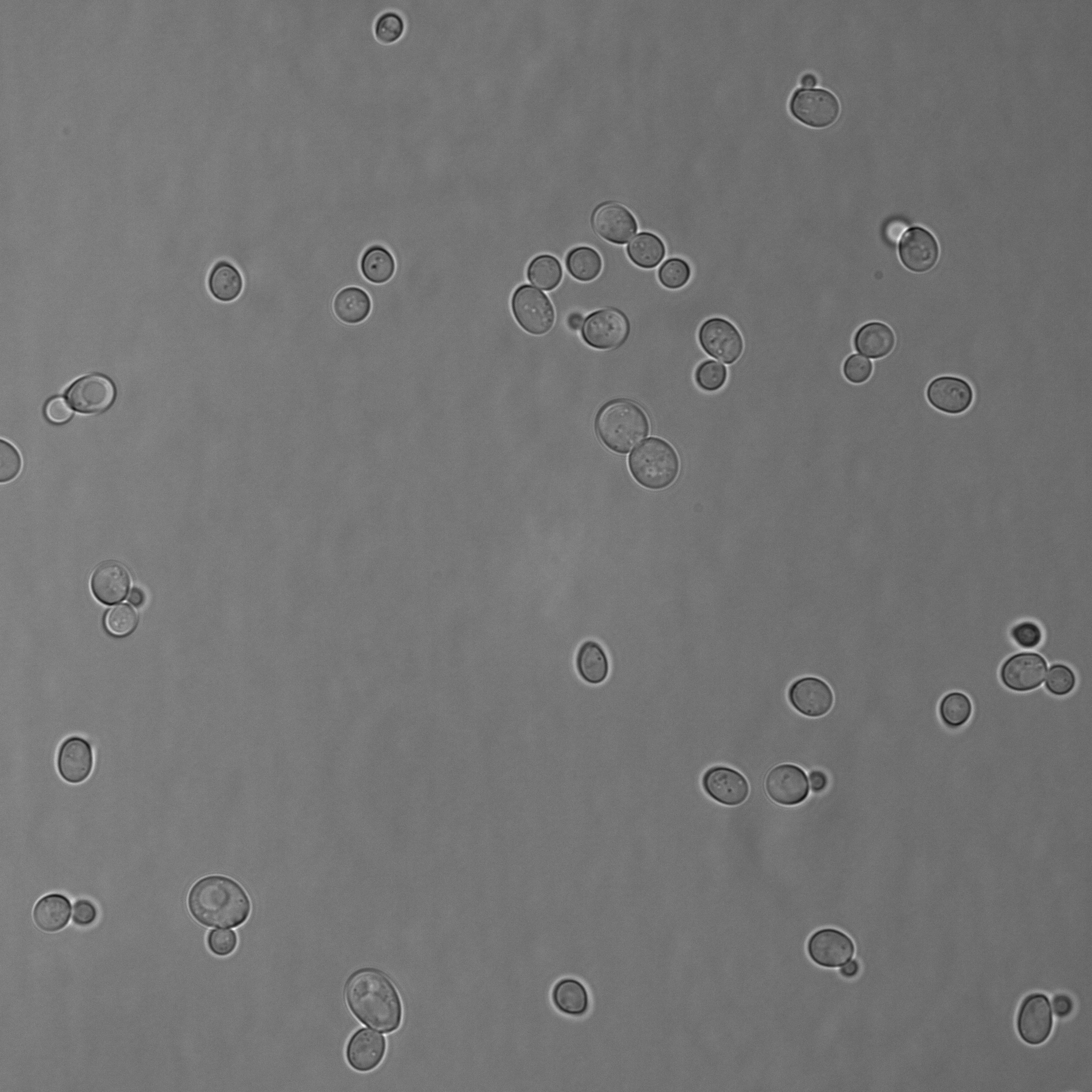

Supplement: Supplementary file 6 — Source Data Fig. 5 [file 44319_2023_55_MOESM6_ESM.zip › Figure 5/5G/Microsocpy_1-13wt-GFP_8D13D19D/BF.tif]

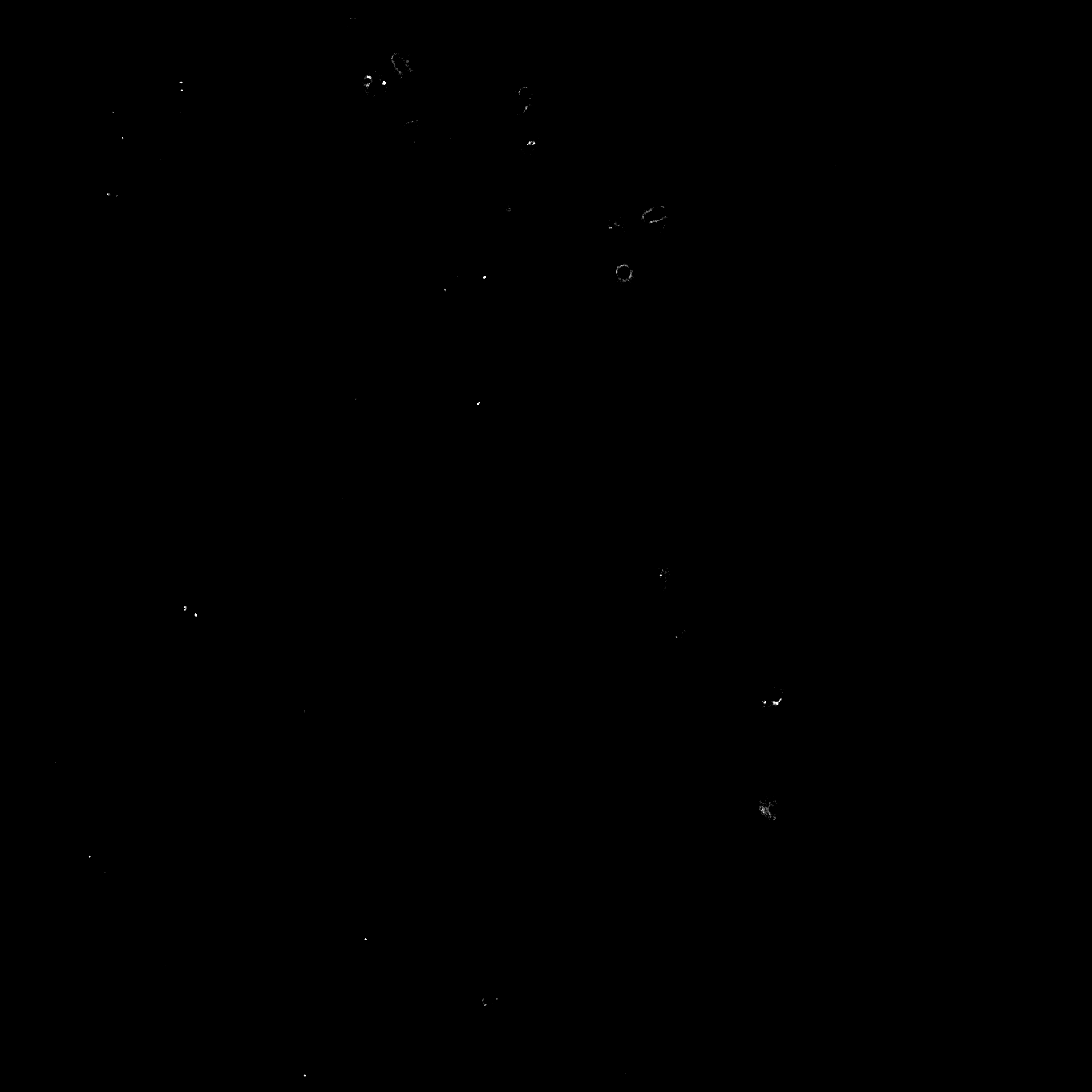

Supplement: Supplementary file 6 — Source Data Fig. 5 [file 44319_2023_55_MOESM6_ESM.zip › Figure 5/5G/Microscopy_13wt-GFP_8D13D19D/GFP.tif]

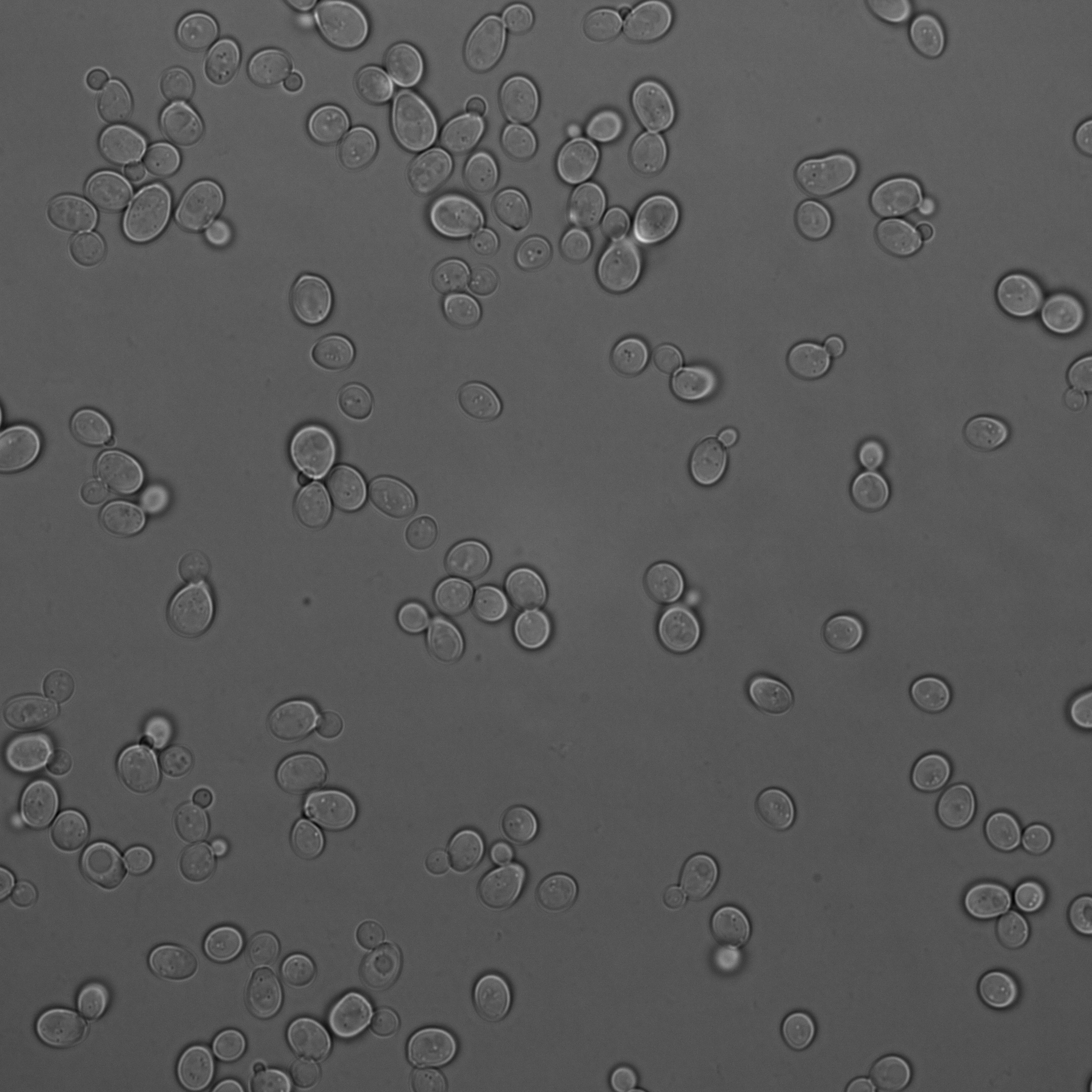

Supplement: Supplementary file 6 — Source Data Fig. 5 [file 44319_2023_55_MOESM6_ESM.zip › Figure 5/5G/Microscopy_13wt-GFP_8D13D19D/BF.tif]

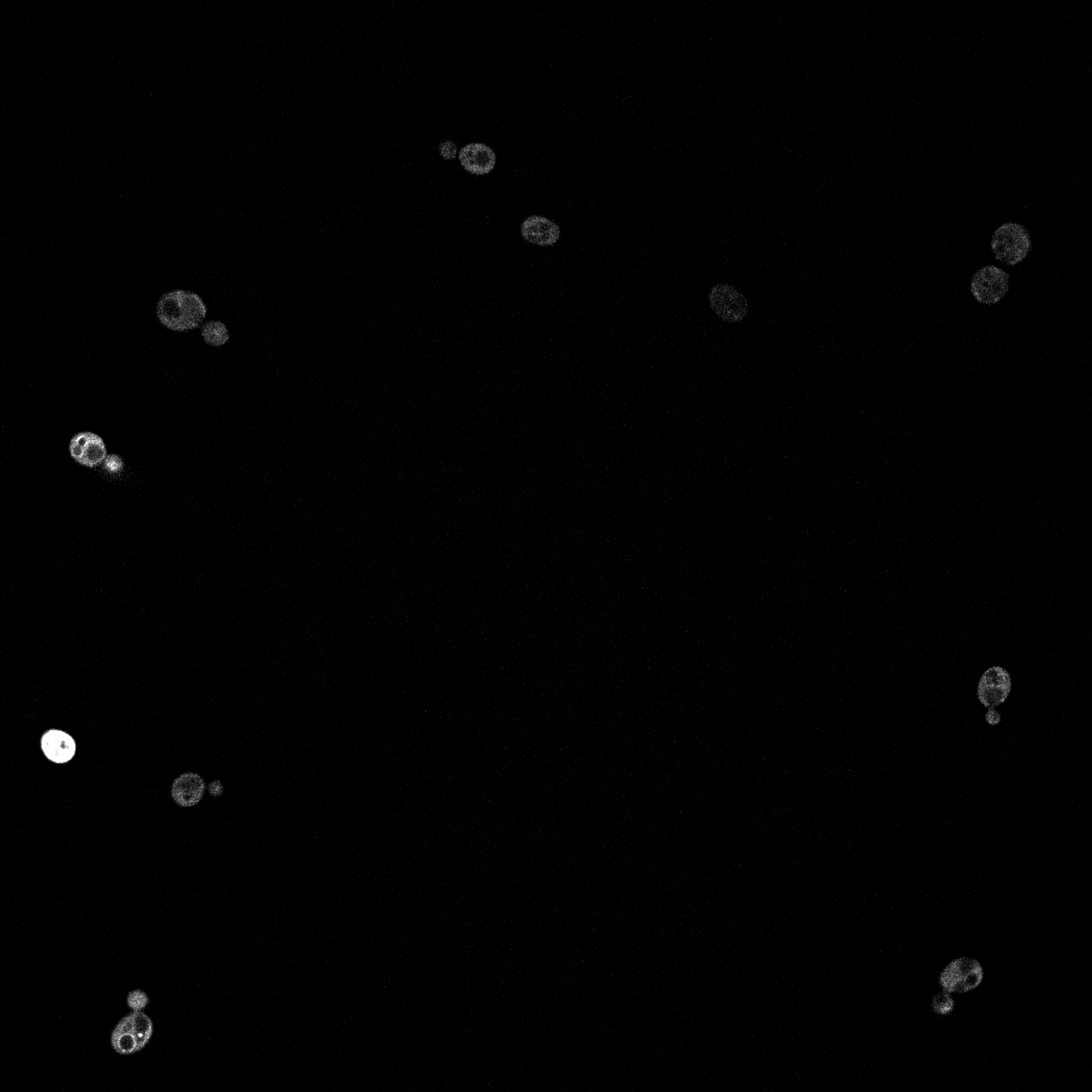

Supplement: Supplementary file 6 — Source Data Fig. 5 [file 44319_2023_55_MOESM6_ESM.zip › Figure 5/5G/Microsocpy_13wt-GFP_8D13D17D19D/GFP.tif]

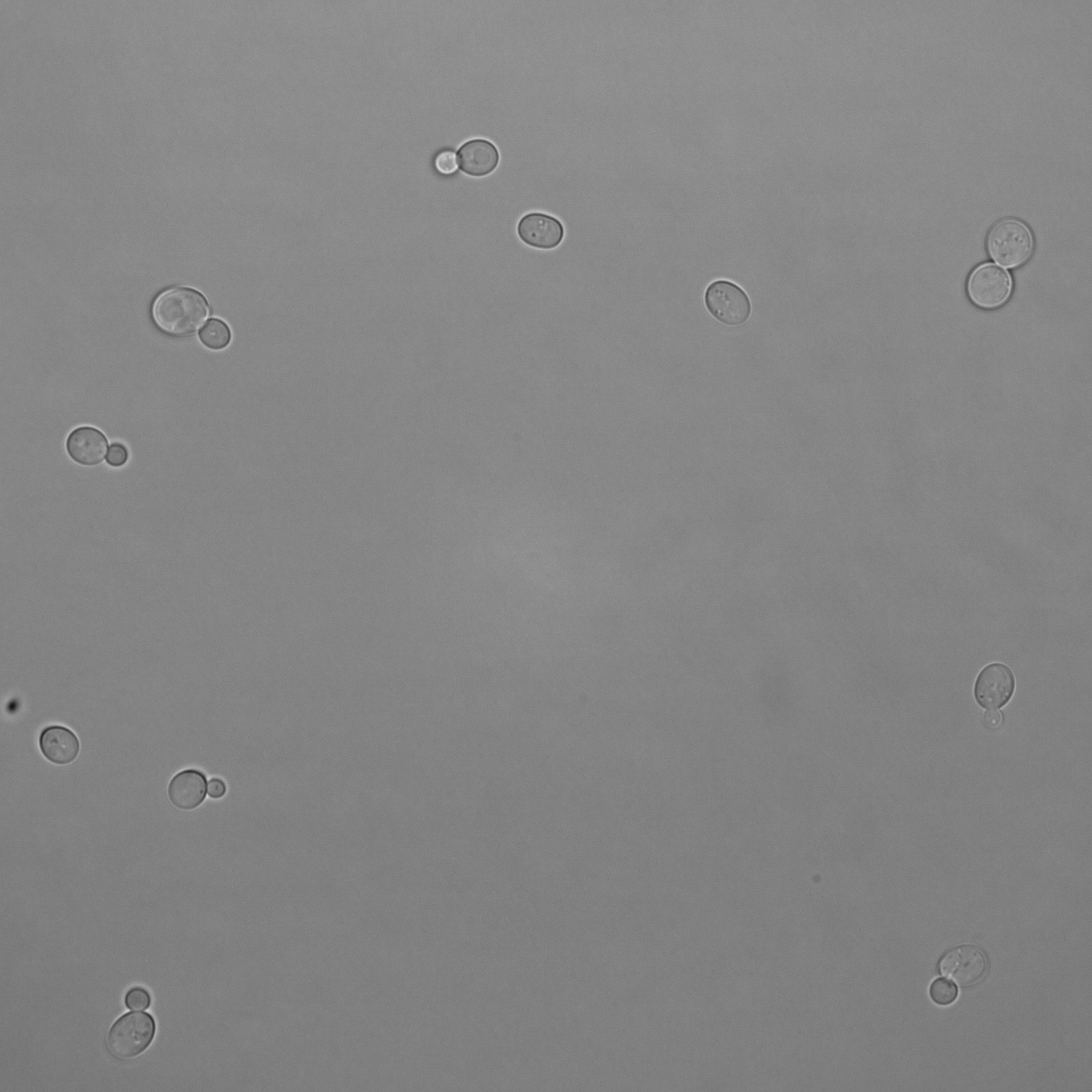

Supplement: Supplementary file 6 — Source Data Fig. 5 [file 44319_2023_55_MOESM6_ESM.zip › Figure 5/5G/Microsocpy_13wt-GFP_8D13D17D19D/BF.tif]

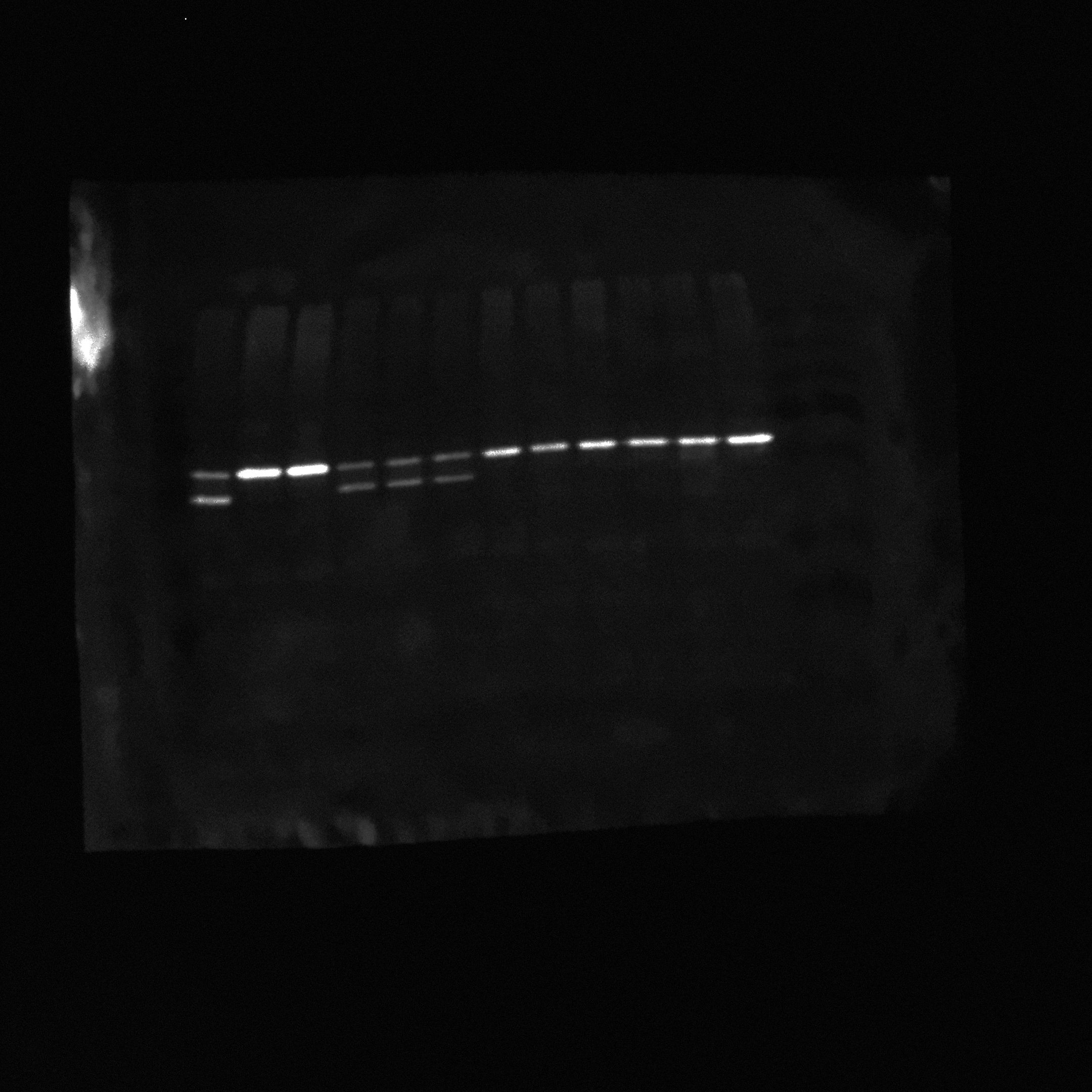

Supplement: Supplementary file 7 — Source Data Fig. 6 [file 44319_2023_55_MOESM7_ESM.zip › Figure 6/6C/Western_Ape1_rich.Tif]

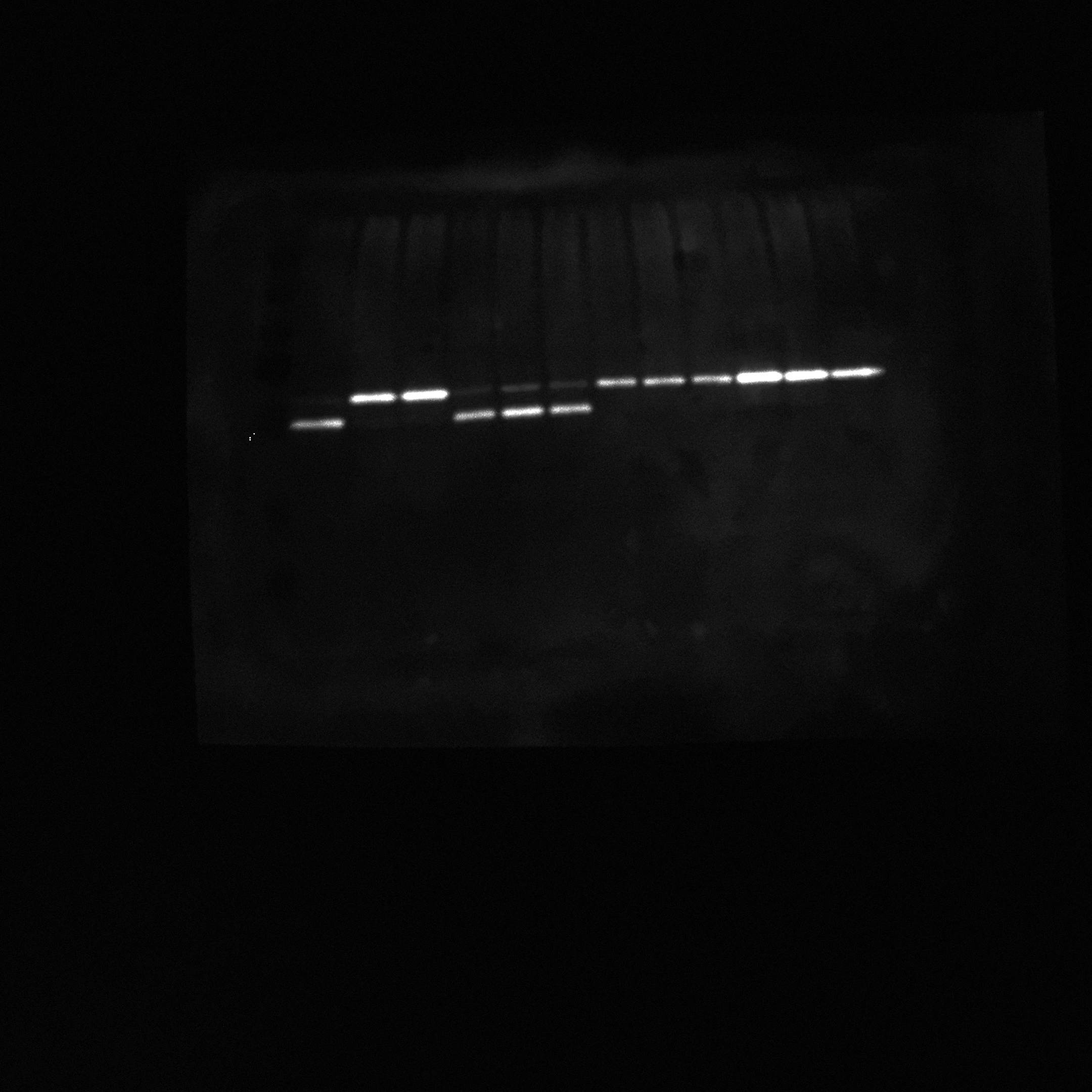

Supplement: Supplementary file 7 — Source Data Fig. 6 [file 44319_2023_55_MOESM7_ESM.zip › Figure 6/6C/Western_Ape1_starvation.Tif]

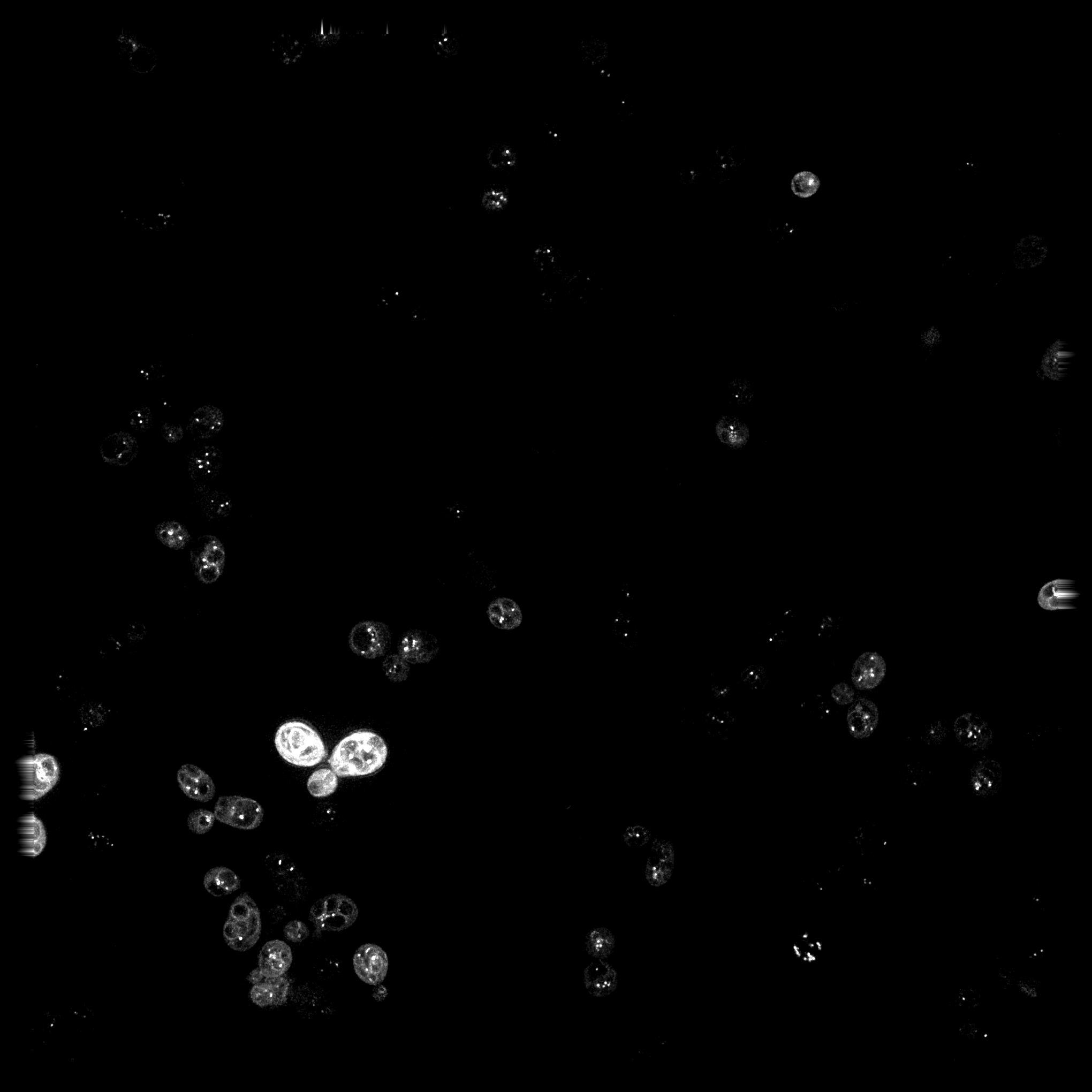

Supplement: Supplementary file 7 — Source Data Fig. 6 [file 44319_2023_55_MOESM7_ESM.zip › Figure 6/6E/Microscopy_Mock/13-GFP_13D19D/GFP.tif]

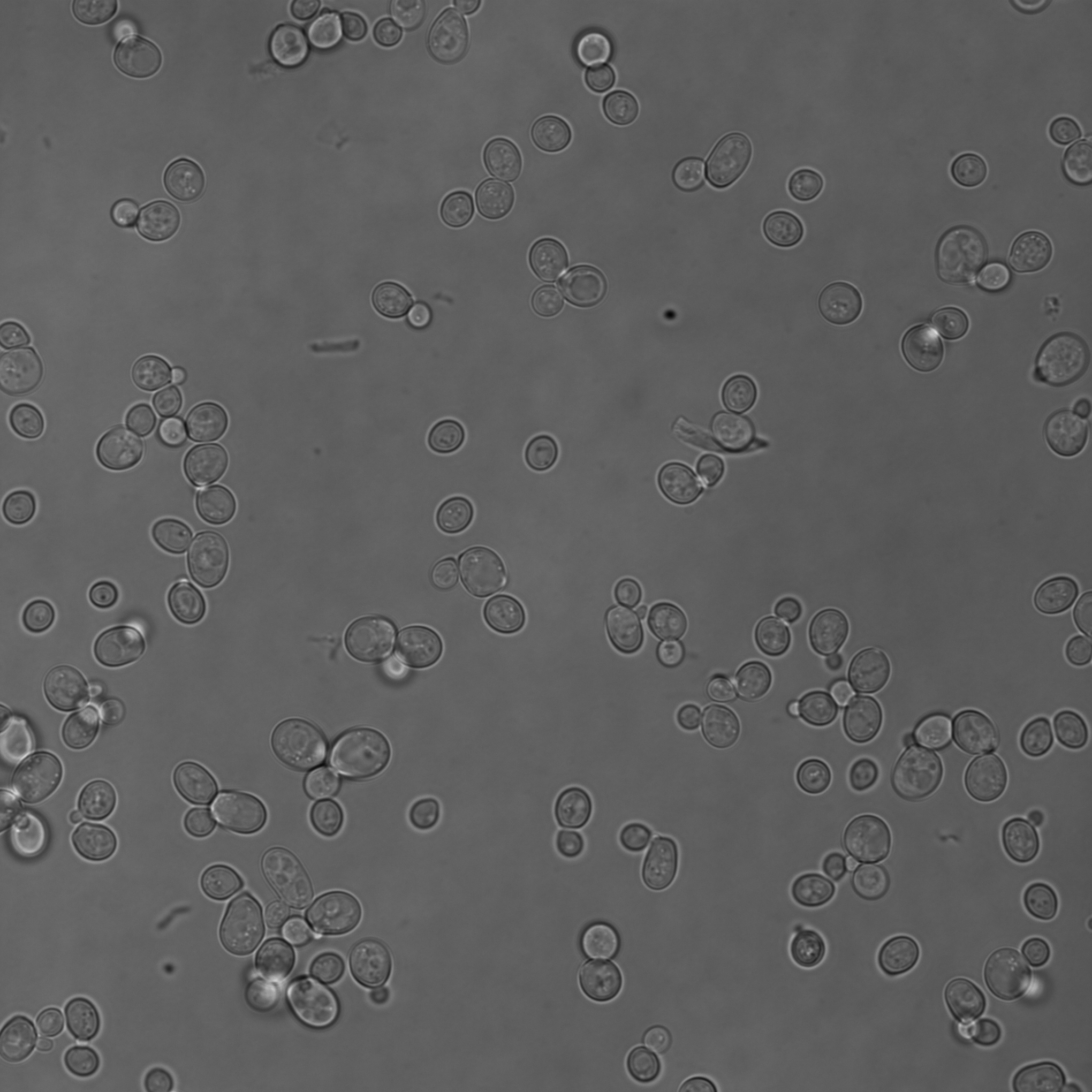

Supplement: Supplementary file 7 — Source Data Fig. 6 [file 44319_2023_55_MOESM7_ESM.zip › Figure 6/6E/Microscopy_Mock/13-GFP_13D19D/BF.tif]

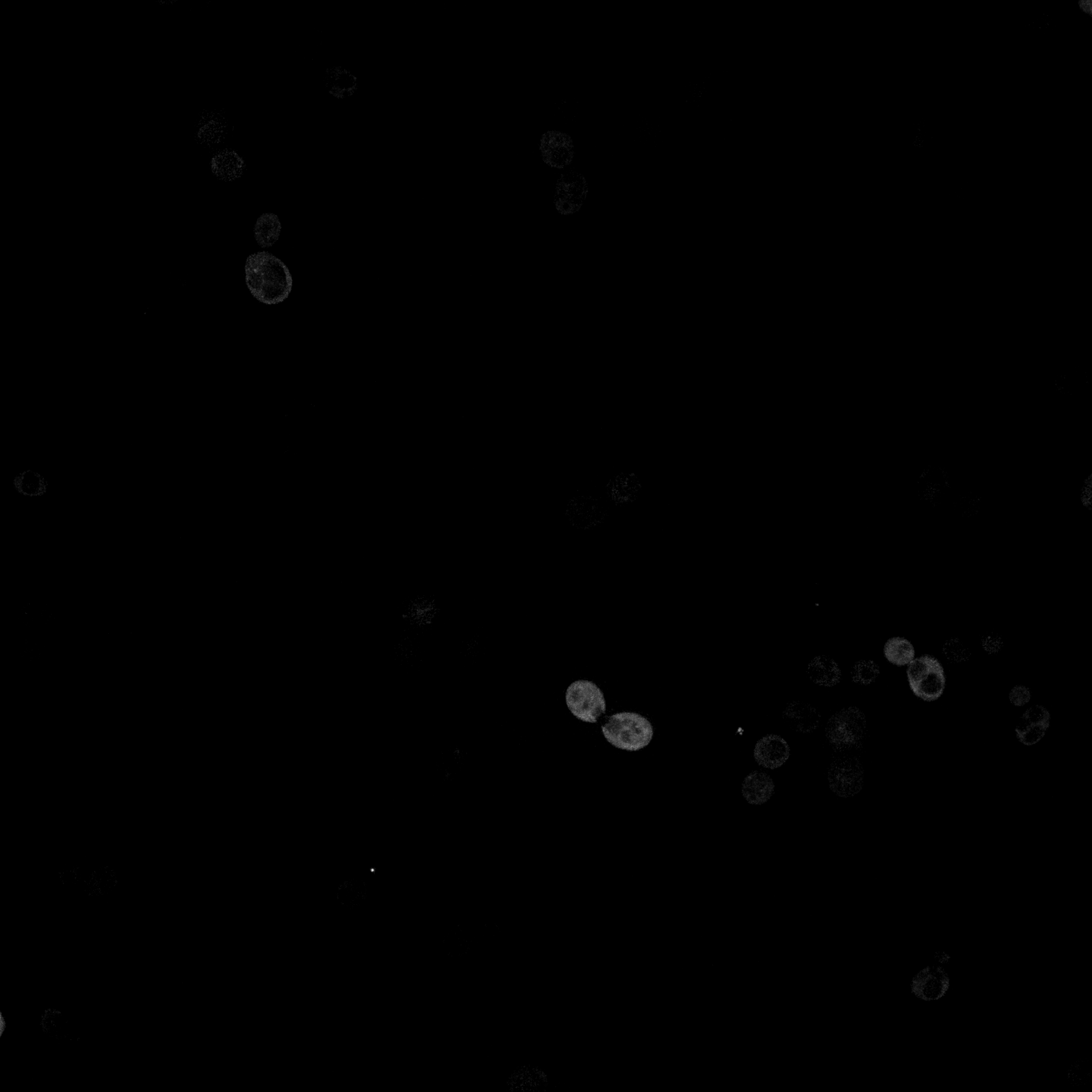

Supplement: Supplementary file 7 — Source Data Fig. 6 [file 44319_2023_55_MOESM7_ESM.zip › Figure 6/6E/Microscopy_Mock/1-1344D-GFP_1D13D19D/GFP.tif]

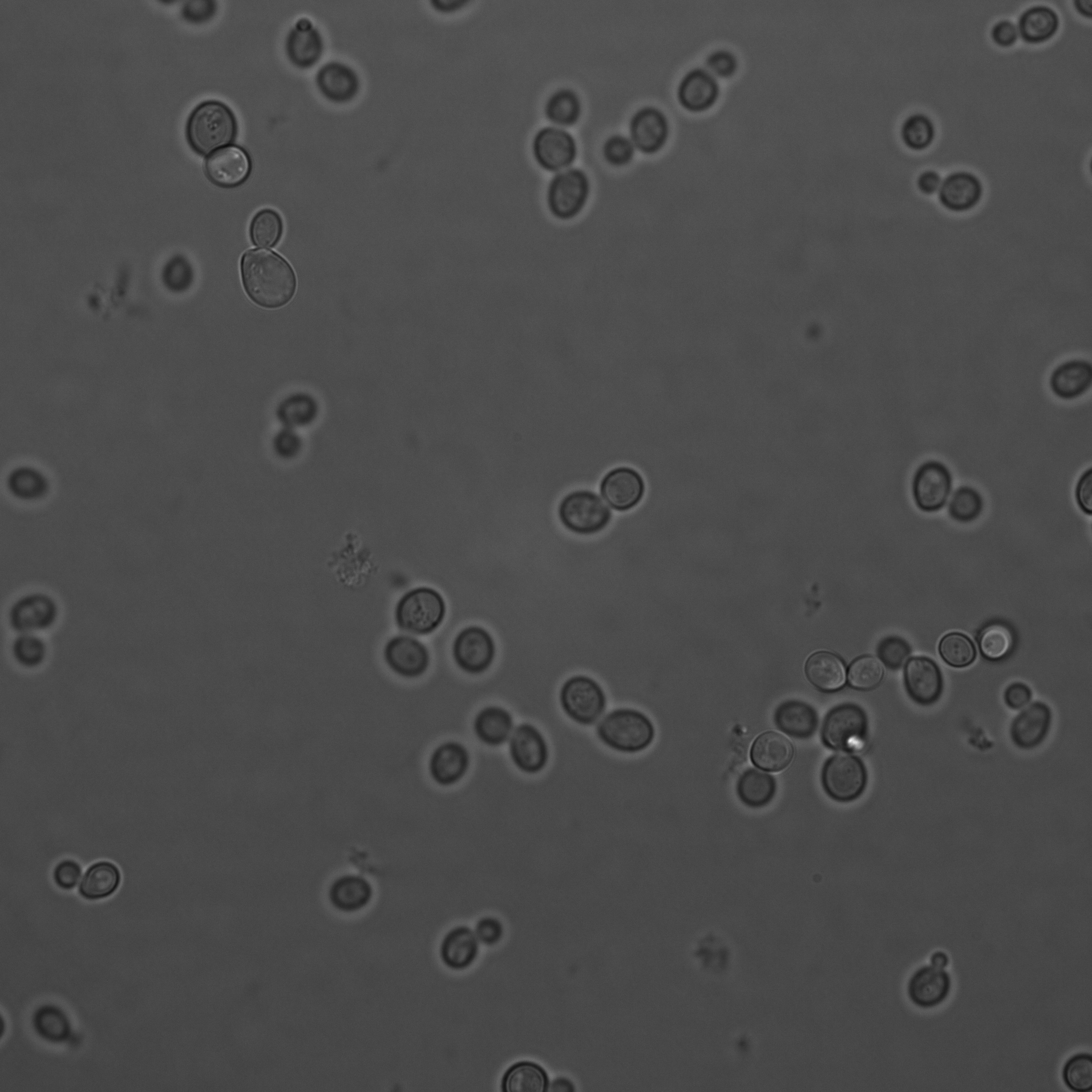

Supplement: Supplementary file 7 — Source Data Fig. 6 [file 44319_2023_55_MOESM7_ESM.zip › Figure 6/6E/Microscopy_Mock/1-1344D-GFP_1D13D19D/BF.tif]

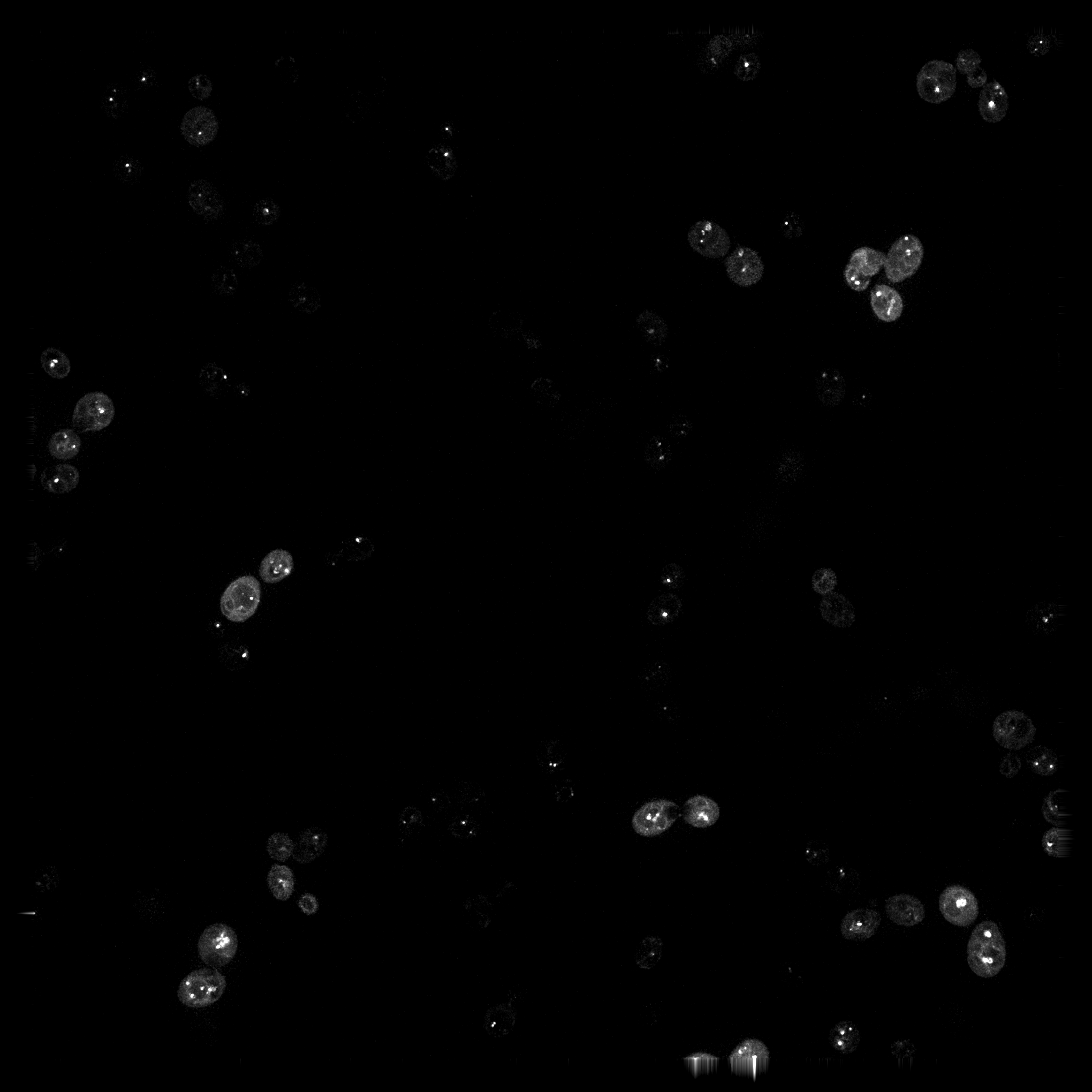

Supplement: Supplementary file 7 — Source Data Fig. 6 [file 44319_2023_55_MOESM7_ESM.zip › Figure 6/6E/Microscopy_Mock/1-13wt-GFP_1D13D19D/GFP.tif]

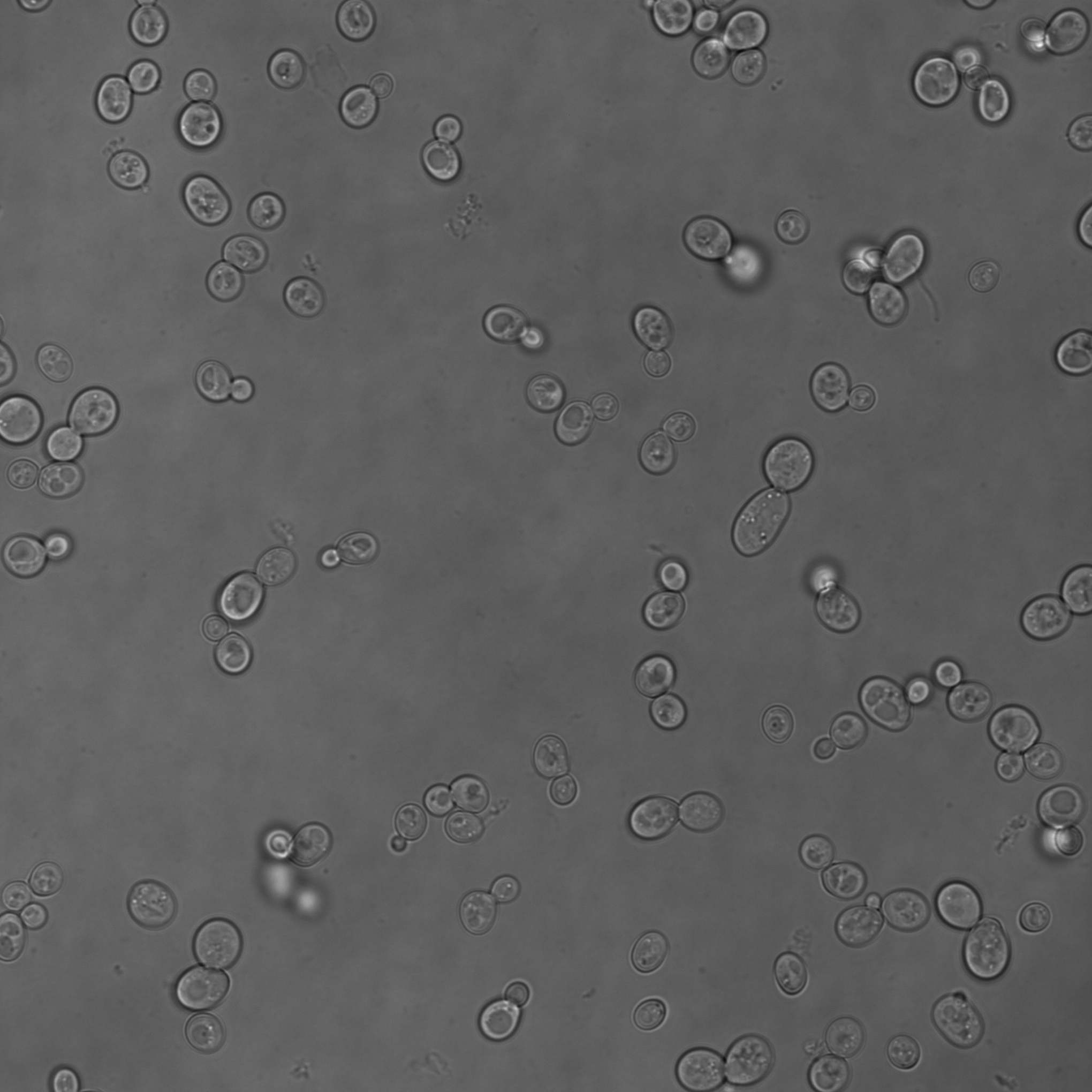

Supplement: Supplementary file 7 — Source Data Fig. 6 [file 44319_2023_55_MOESM7_ESM.zip › Figure 6/6E/Microscopy_Mock/1-13wt-GFP_1D13D19D/BF.tif]

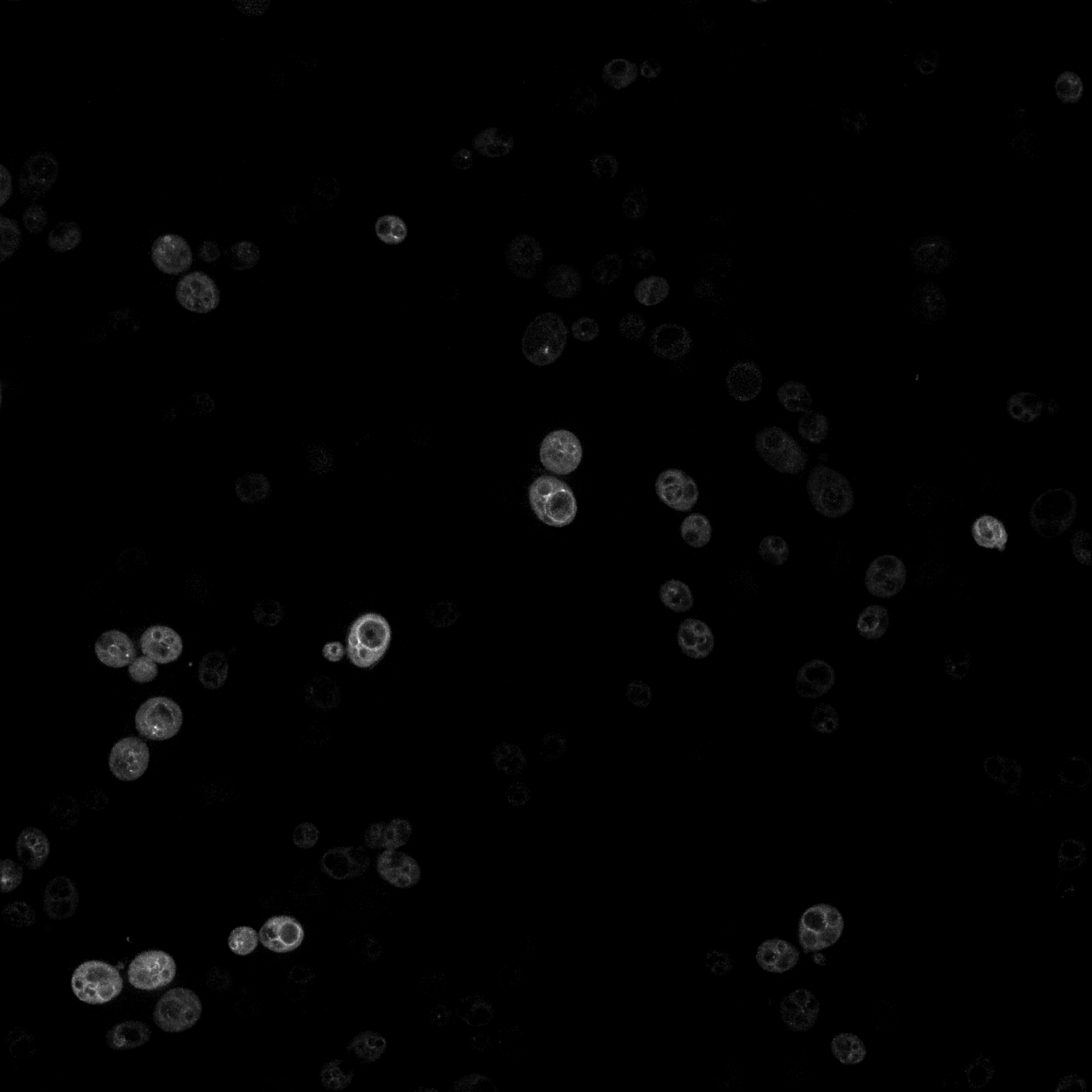

Supplement: Supplementary file 7 — Source Data Fig. 6 [file 44319_2023_55_MOESM7_ESM.zip › Figure 6/6E/Microscopy_Mock/1-13MD-GFP_1D13D19D/GFP.tif]

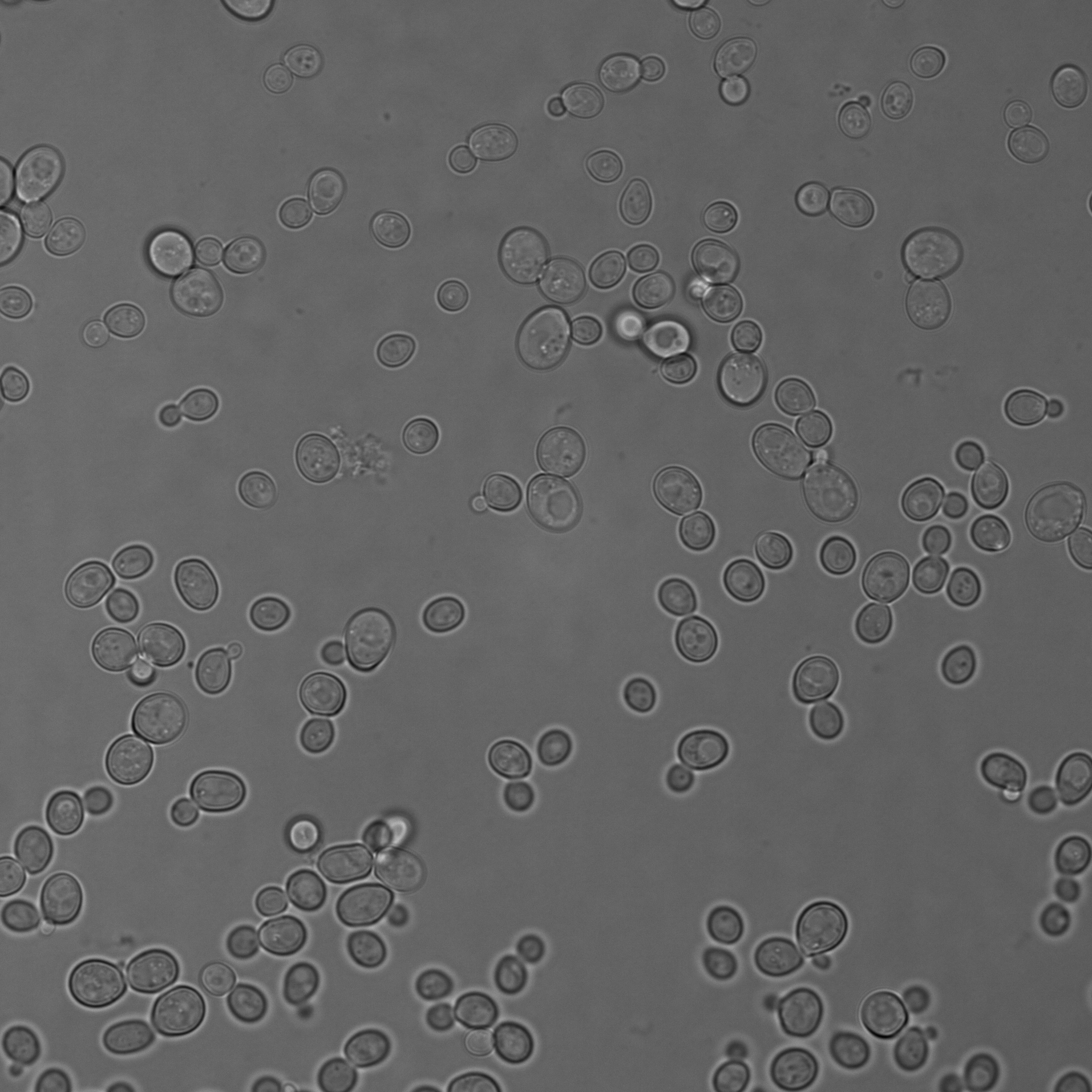

Supplement: Supplementary file 7 — Source Data Fig. 6 [file 44319_2023_55_MOESM7_ESM.zip › Figure 6/6E/Microscopy_Mock/1-13MD-GFP_1D13D19D/BF.tif]

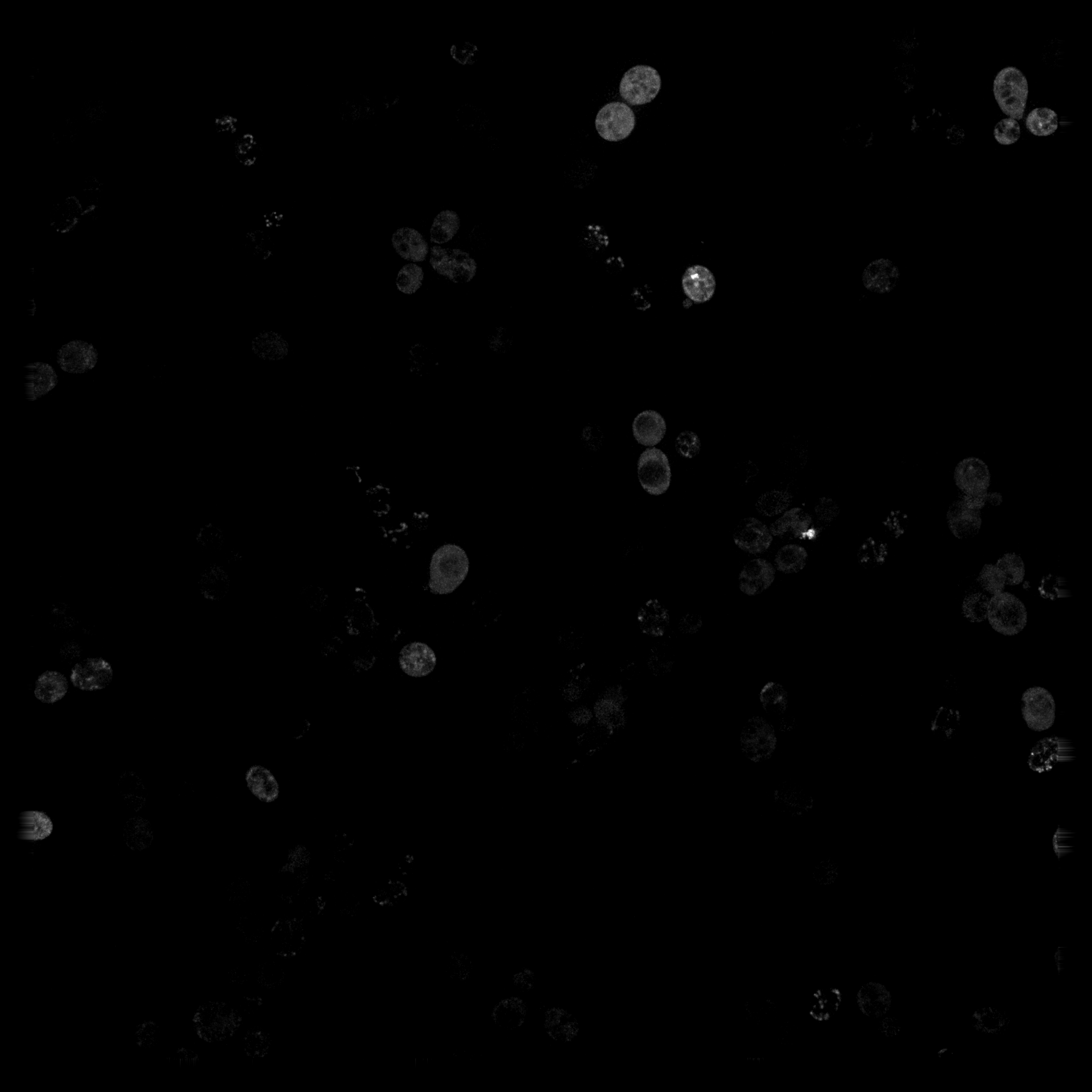

Supplement: Supplementary file 7 — Source Data Fig. 6 [file 44319_2023_55_MOESM7_ESM.zip › Figure 6/6E/Microscopy_hexanediol/13wt-GFP_13D19D/GFP.tif]

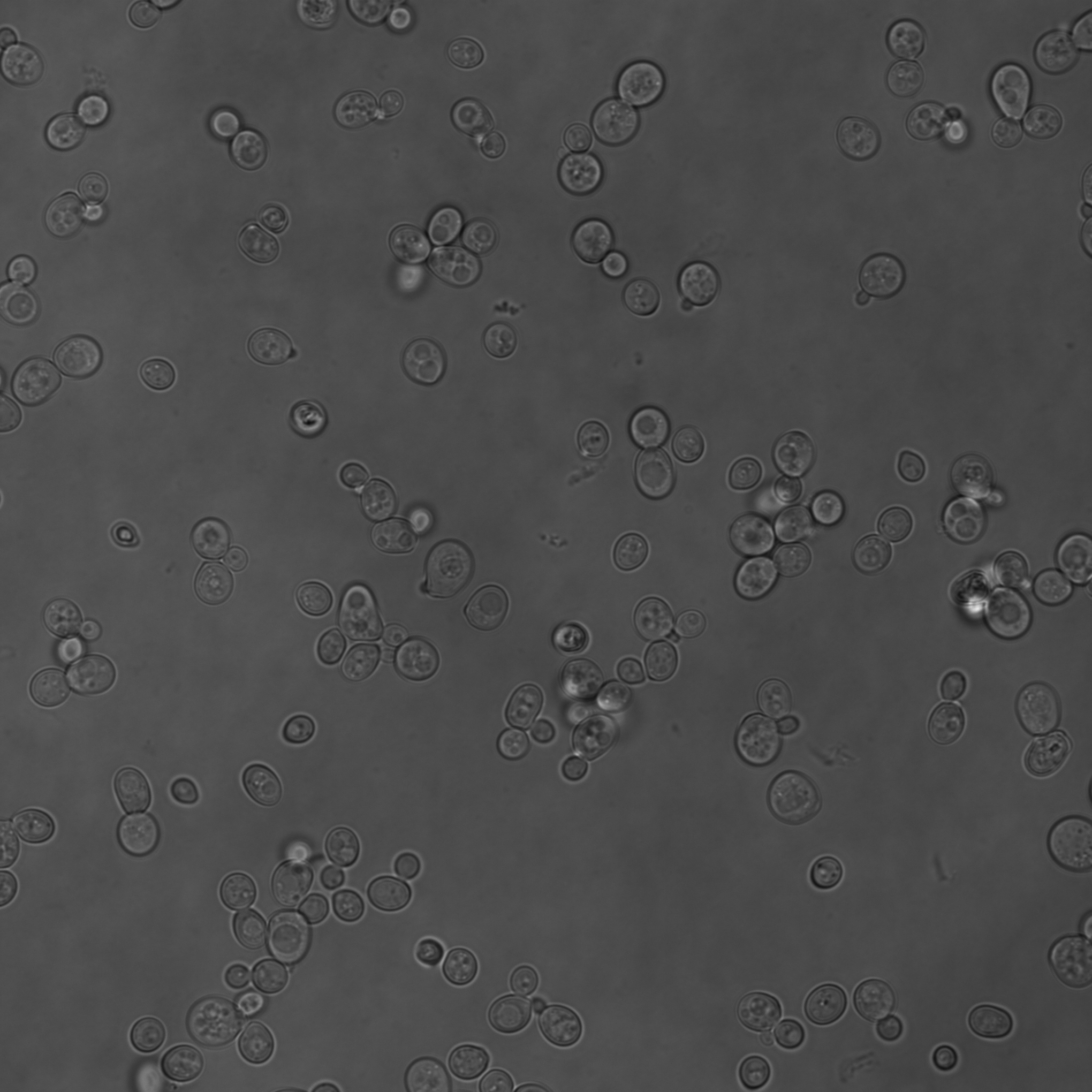

Supplement: Supplementary file 7 — Source Data Fig. 6 [file 44319_2023_55_MOESM7_ESM.zip › Figure 6/6E/Microscopy_hexanediol/13wt-GFP_13D19D/BF.tif]

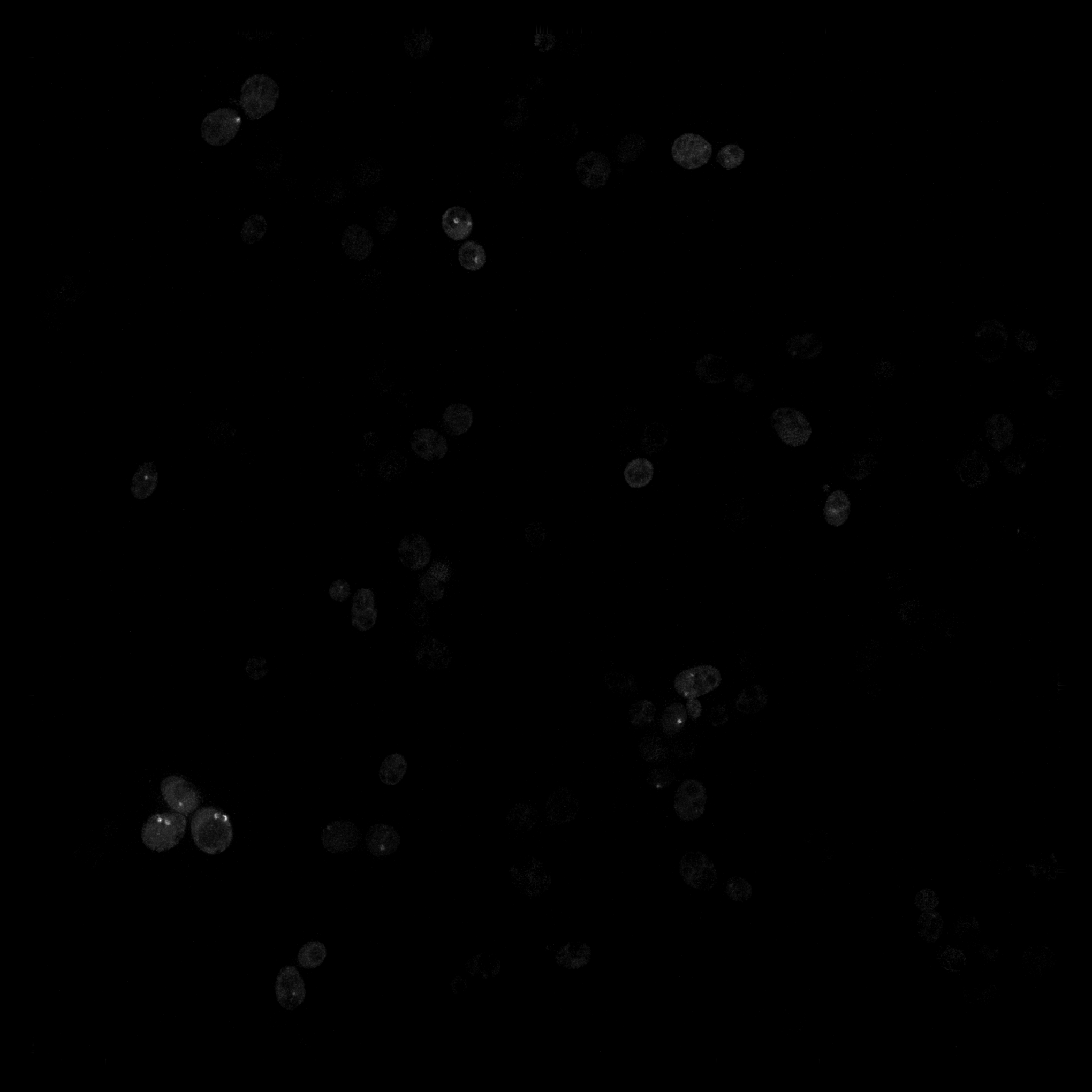

Supplement: Supplementary file 7 — Source Data Fig. 6 [file 44319_2023_55_MOESM7_ESM.zip › Figure 6/6E/Microscopy_hexanediol/1-13wt_GFP_1D13D19D/GFP.tif]

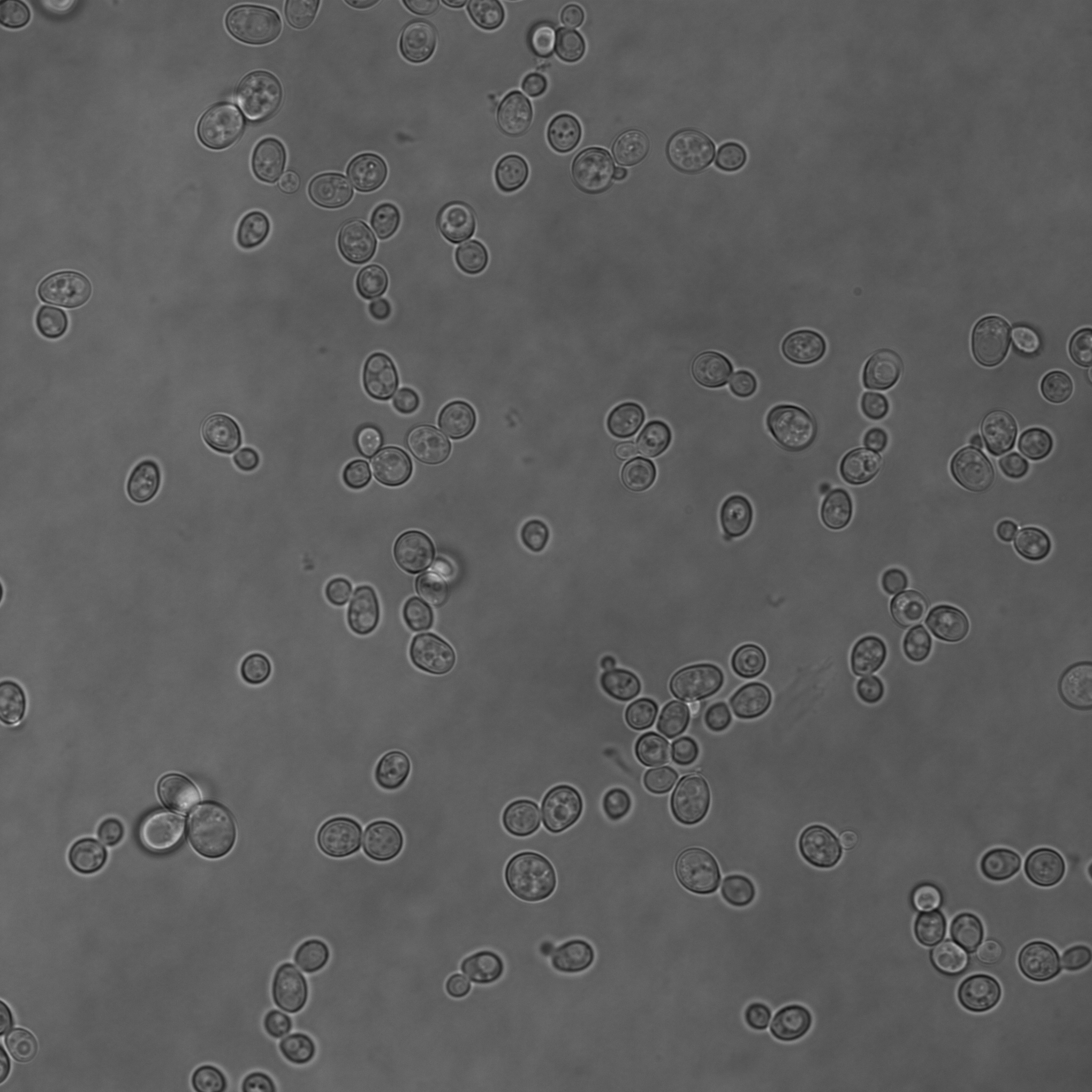

Supplement: Supplementary file 7 — Source Data Fig. 6 [file 44319_2023_55_MOESM7_ESM.zip › Figure 6/6E/Microscopy_hexanediol/1-13wt_GFP_1D13D19D/BF.tif]

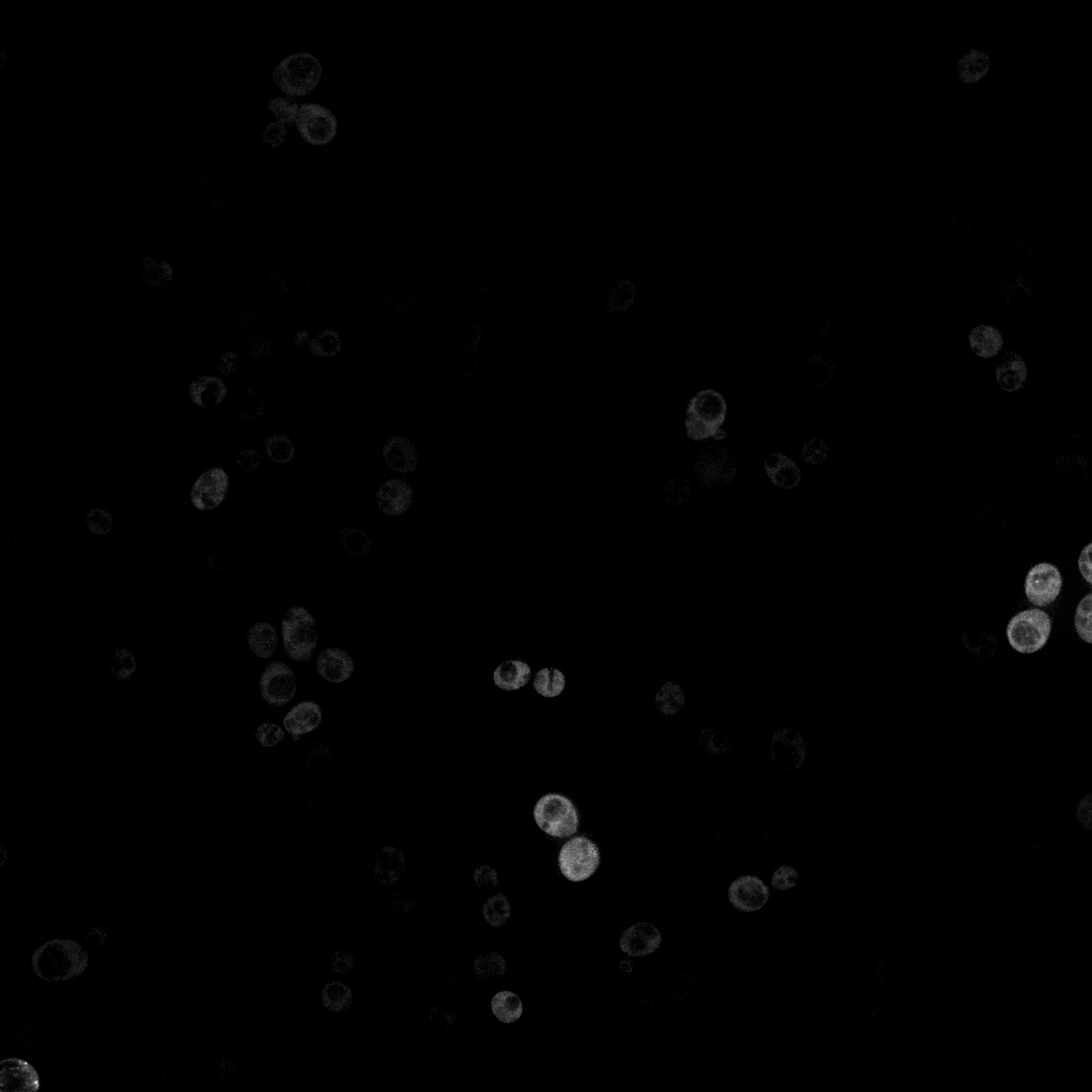

Supplement: Supplementary file 7 — Source Data Fig. 6 [file 44319_2023_55_MOESM7_ESM.zip › Figure 6/6E/Microscopy_hexanediol/1-13MD-GFP_1D13D19D/GFP.tif]

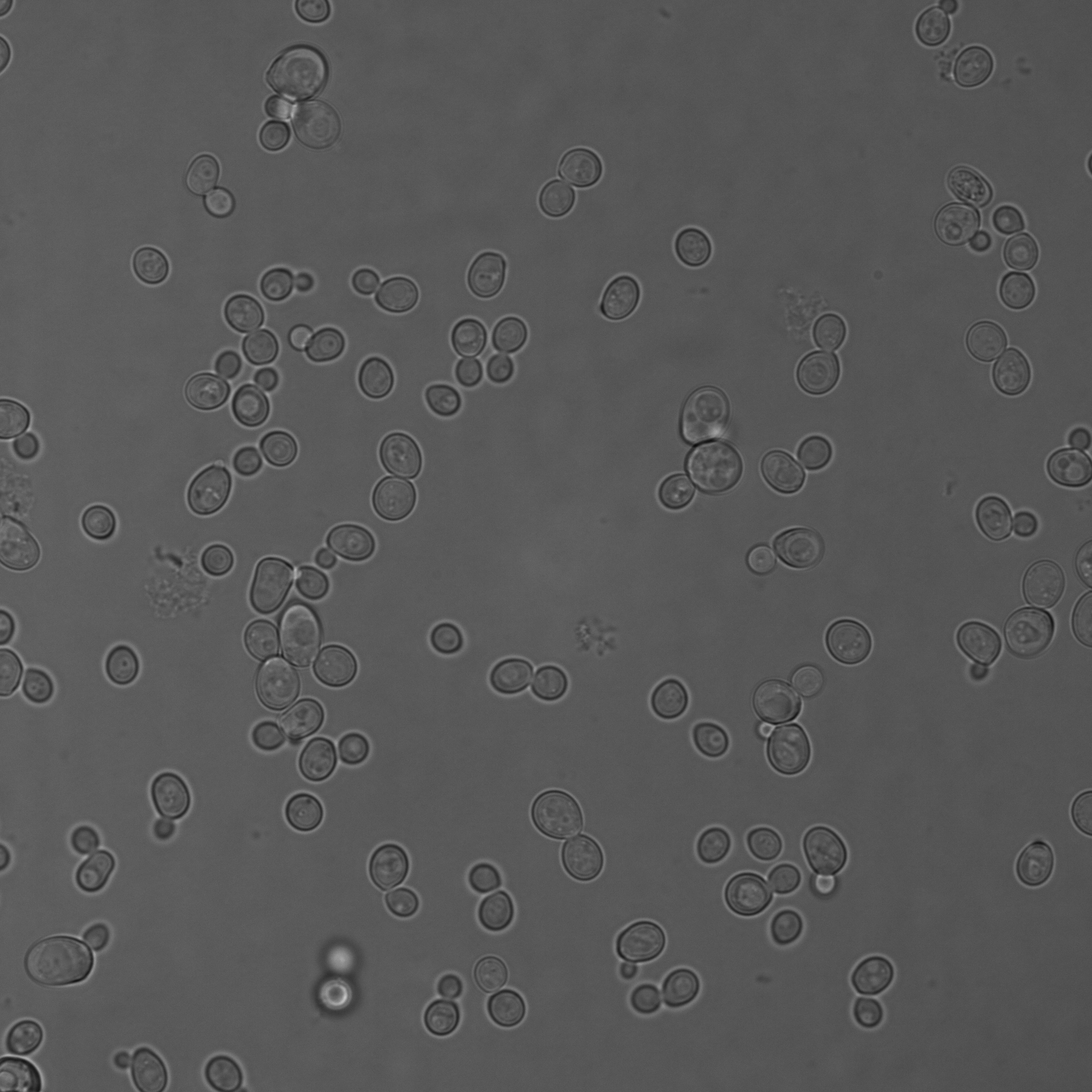

Supplement: Supplementary file 7 — Source Data Fig. 6 [file 44319_2023_55_MOESM7_ESM.zip › Figure 6/6E/Microscopy_hexanediol/1-13MD-GFP_1D13D19D/BF.tif]

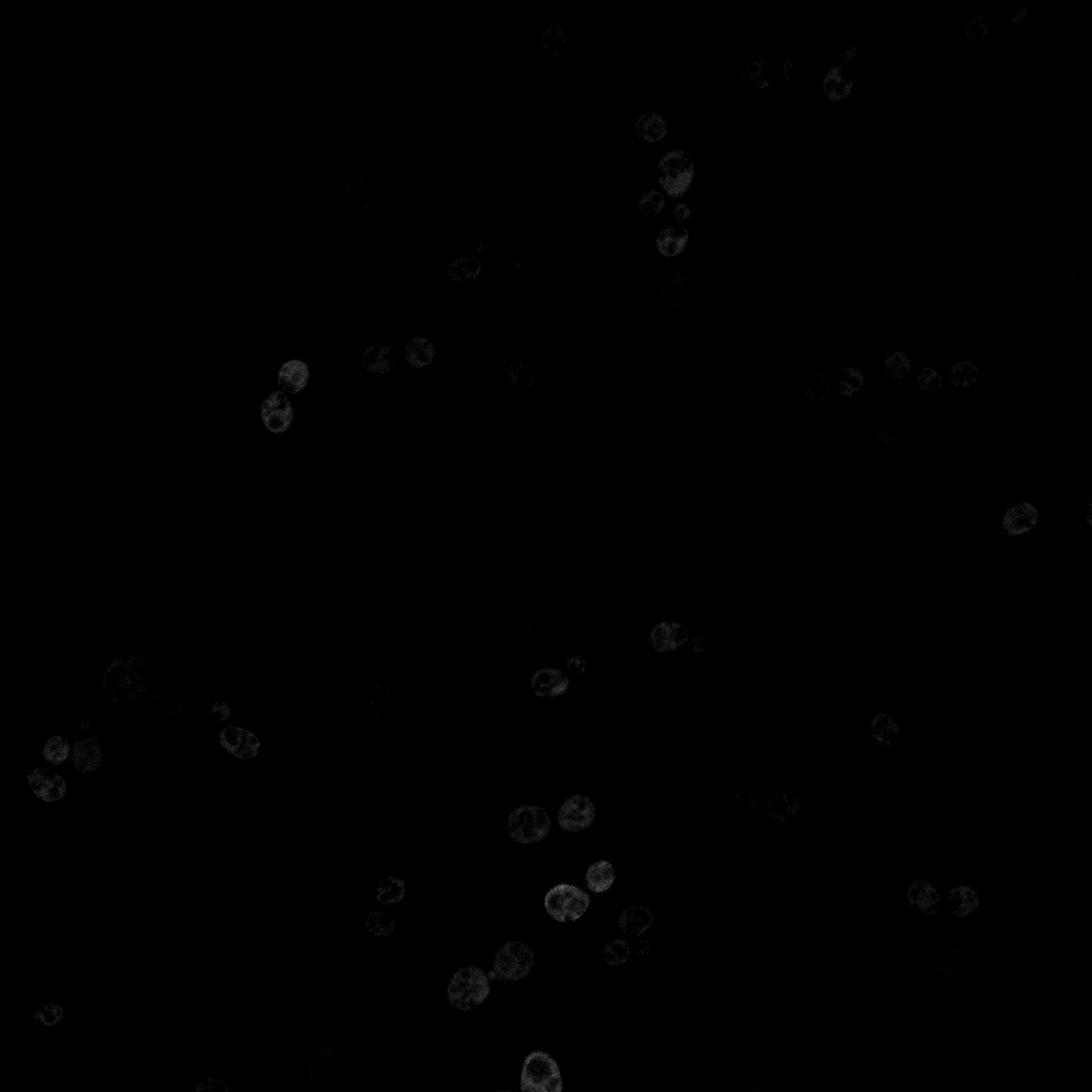

Supplement: Supplementary file 7 — Source Data Fig. 6 [file 44319_2023_55_MOESM7_ESM.zip › Figure 6/6E/Microscopy_hexanediol/1-13_44D-GFP_1D13D19D/GFP.tif]

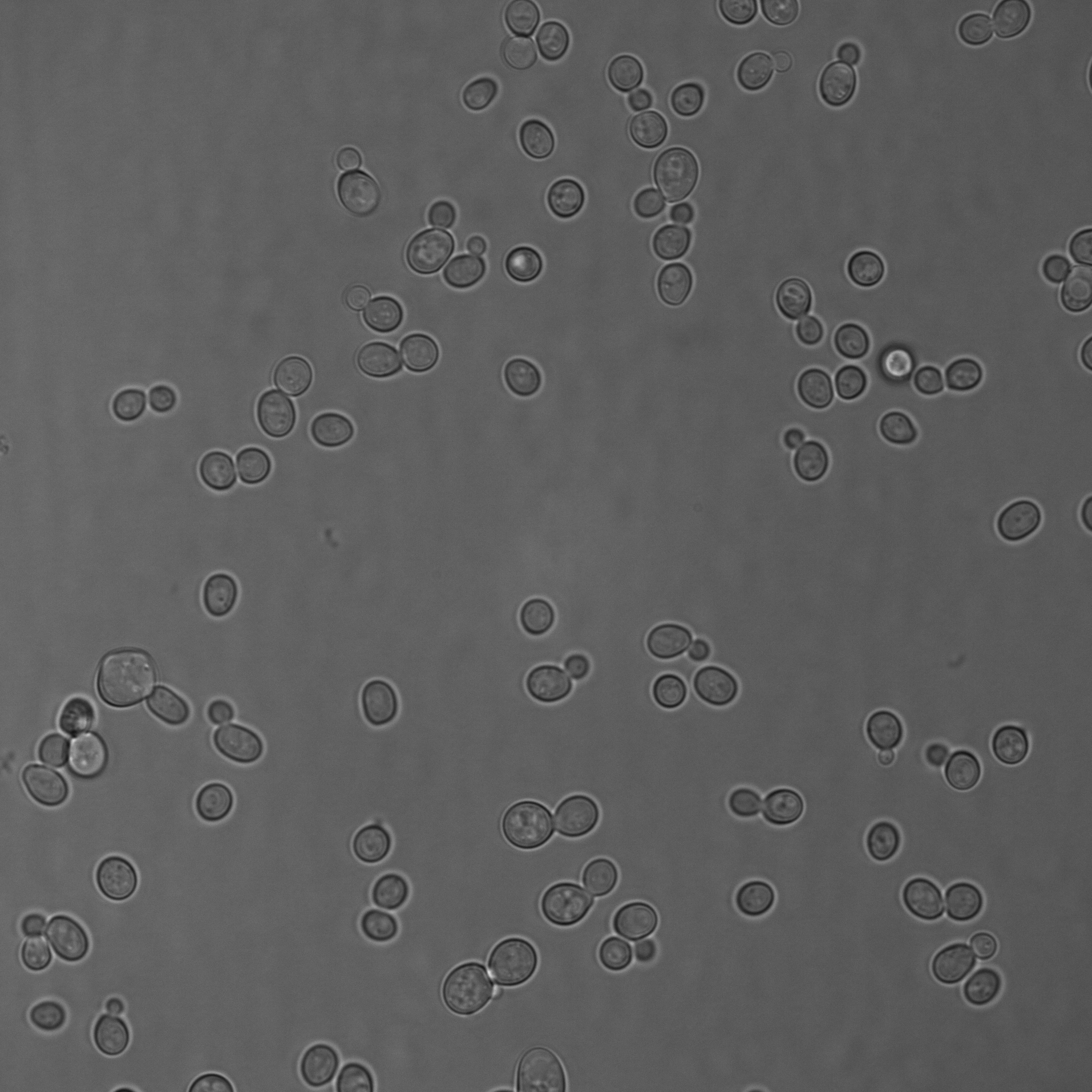

Supplement: Supplementary file 7 — Source Data Fig. 6 [file 44319_2023_55_MOESM7_ESM.zip › Figure 6/6E/Microscopy_hexanediol/1-13_44D-GFP_1D13D19D/BF.tif]

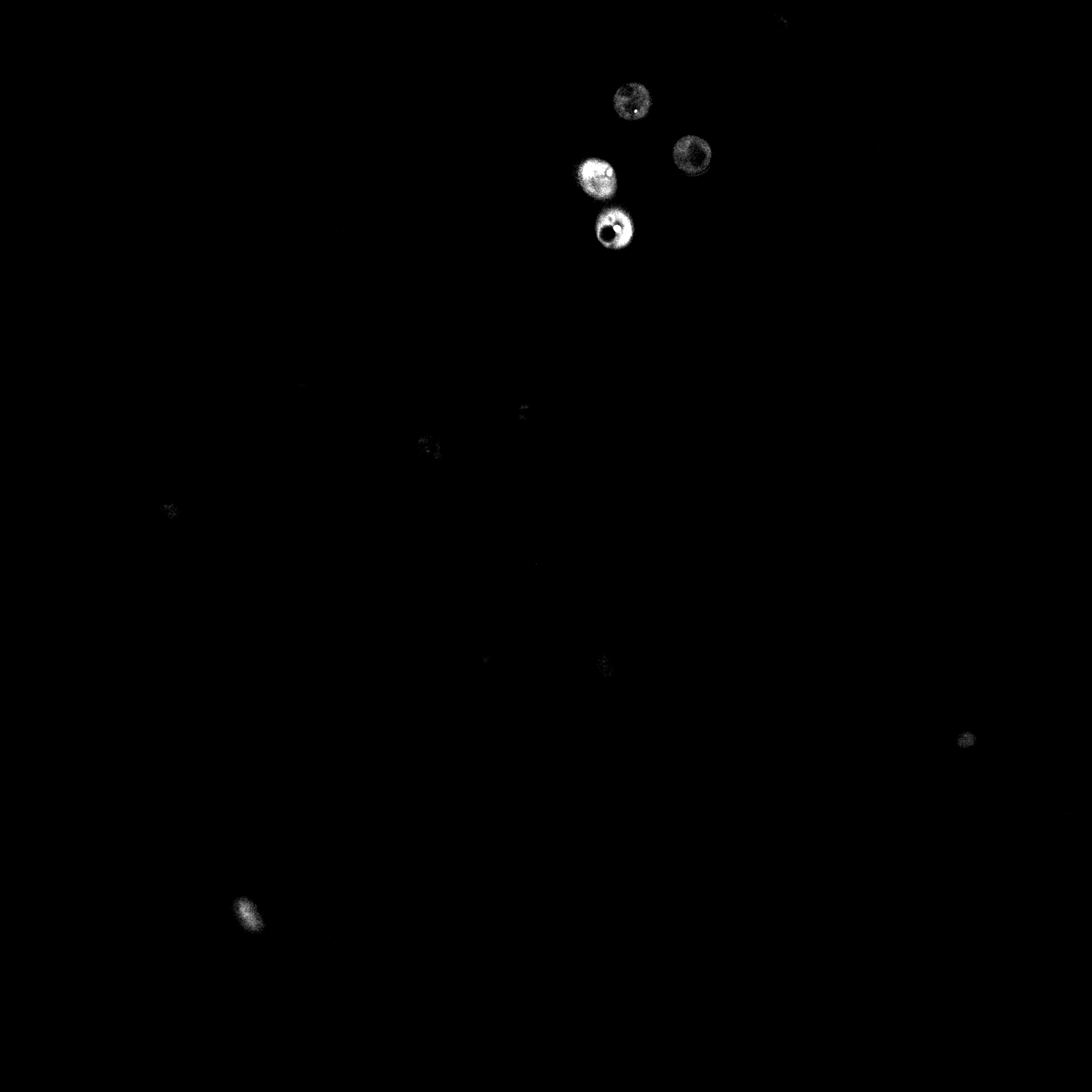

Supplement: Supplementary file 7 — Source Data Fig. 6 [file 44319_2023_55_MOESM7_ESM.zip › Figure 6/6D/Microsocpy_1D11D13D19D/Atg1-Atg13_GFP/GFP.tif]

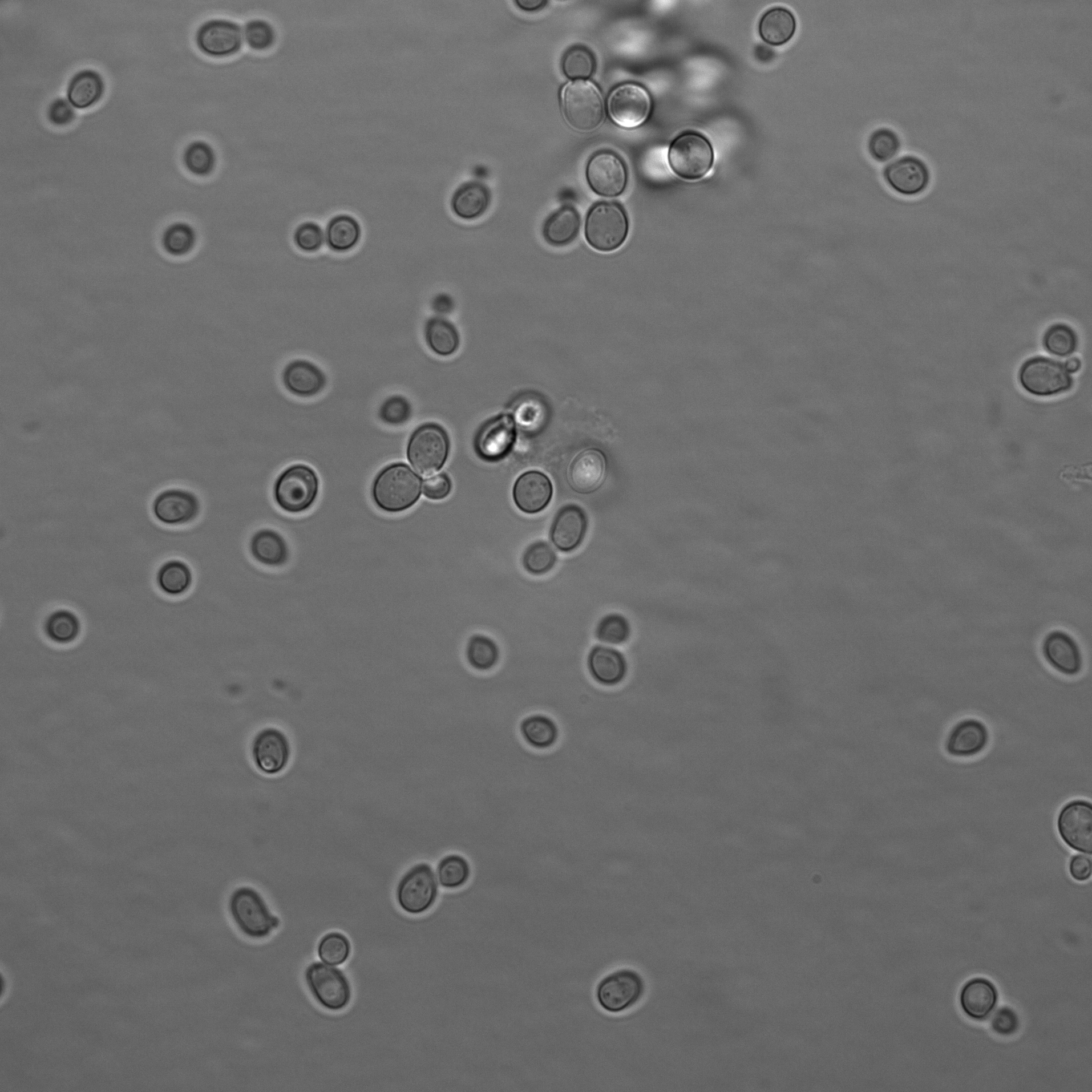

Supplement: Supplementary file 7 — Source Data Fig. 6 [file 44319_2023_55_MOESM7_ESM.zip › Figure 6/6D/Microsocpy_1D11D13D19D/Atg1-Atg13_GFP/BF.tif]

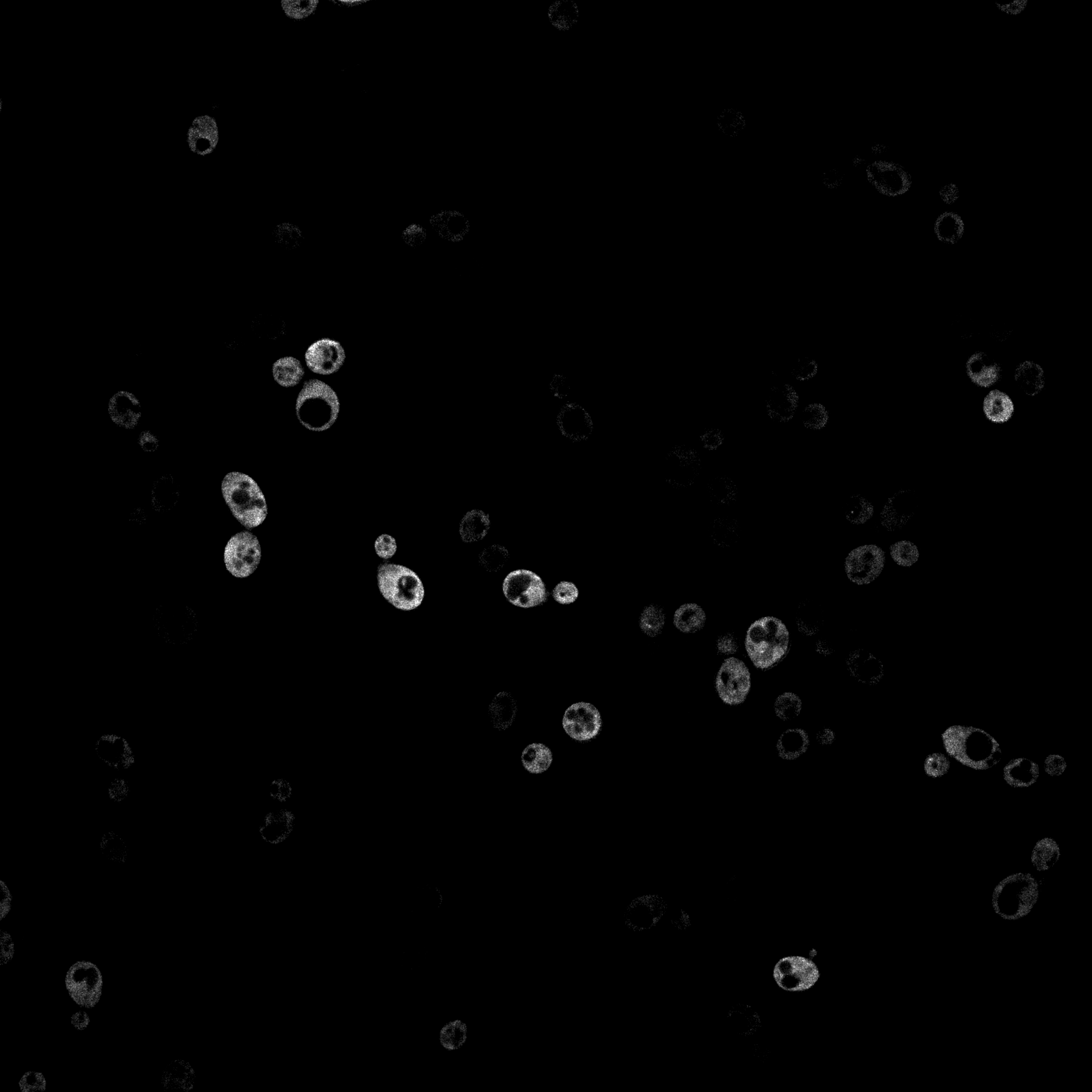

Supplement: Supplementary file 7 — Source Data Fig. 6 [file 44319_2023_55_MOESM7_ESM.zip › Figure 6/6D/Microsocpy_1D11D13D19D/Atg1-GFP/Atg1_GFP_Green_1D11D13D19D.tif]

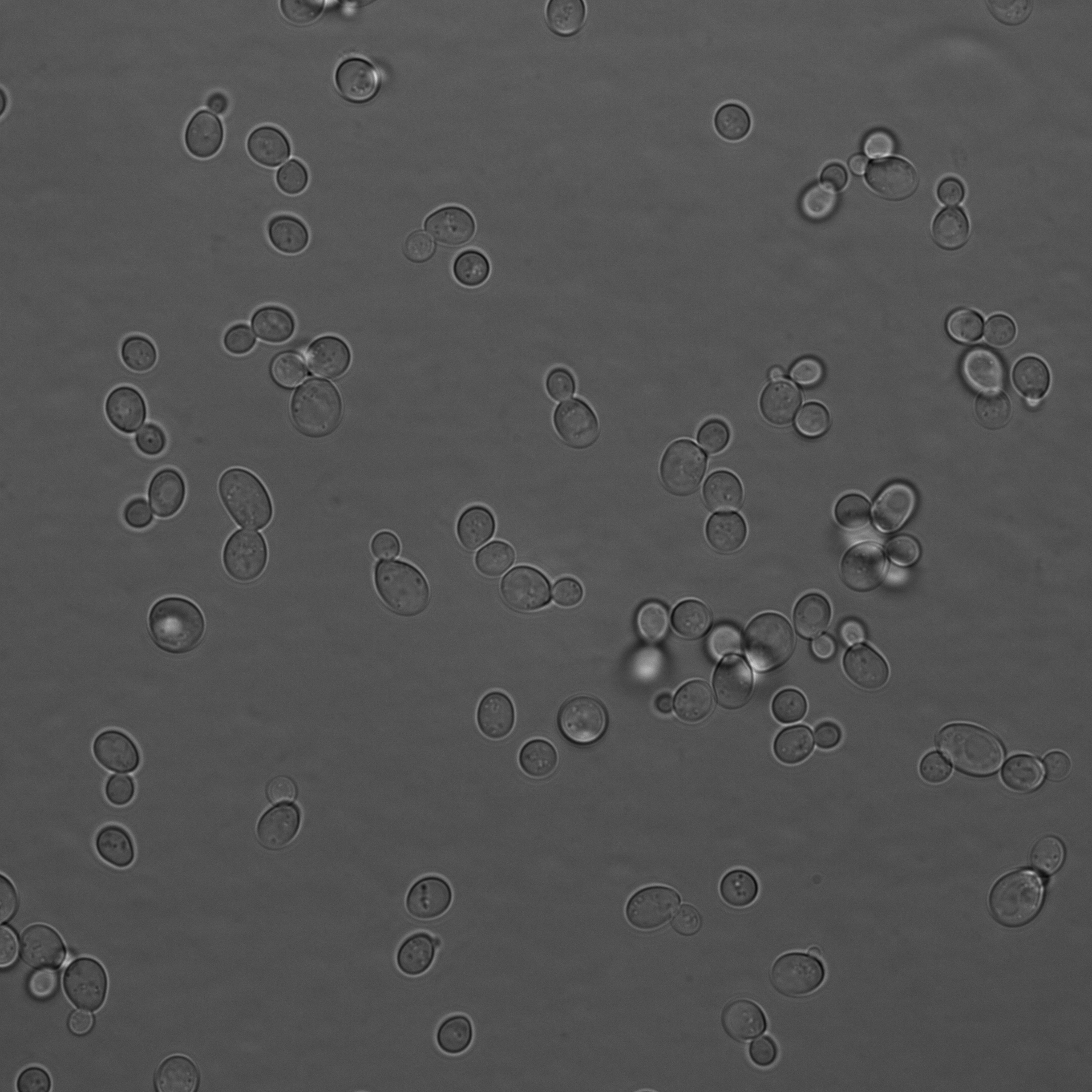

Supplement: Supplementary file 7 — Source Data Fig. 6 [file 44319_2023_55_MOESM7_ESM.zip › Figure 6/6D/Microsocpy_1D11D13D19D/Atg1-GFP/Atg1-GFP_brightfield_1D11D13D19D.tif]

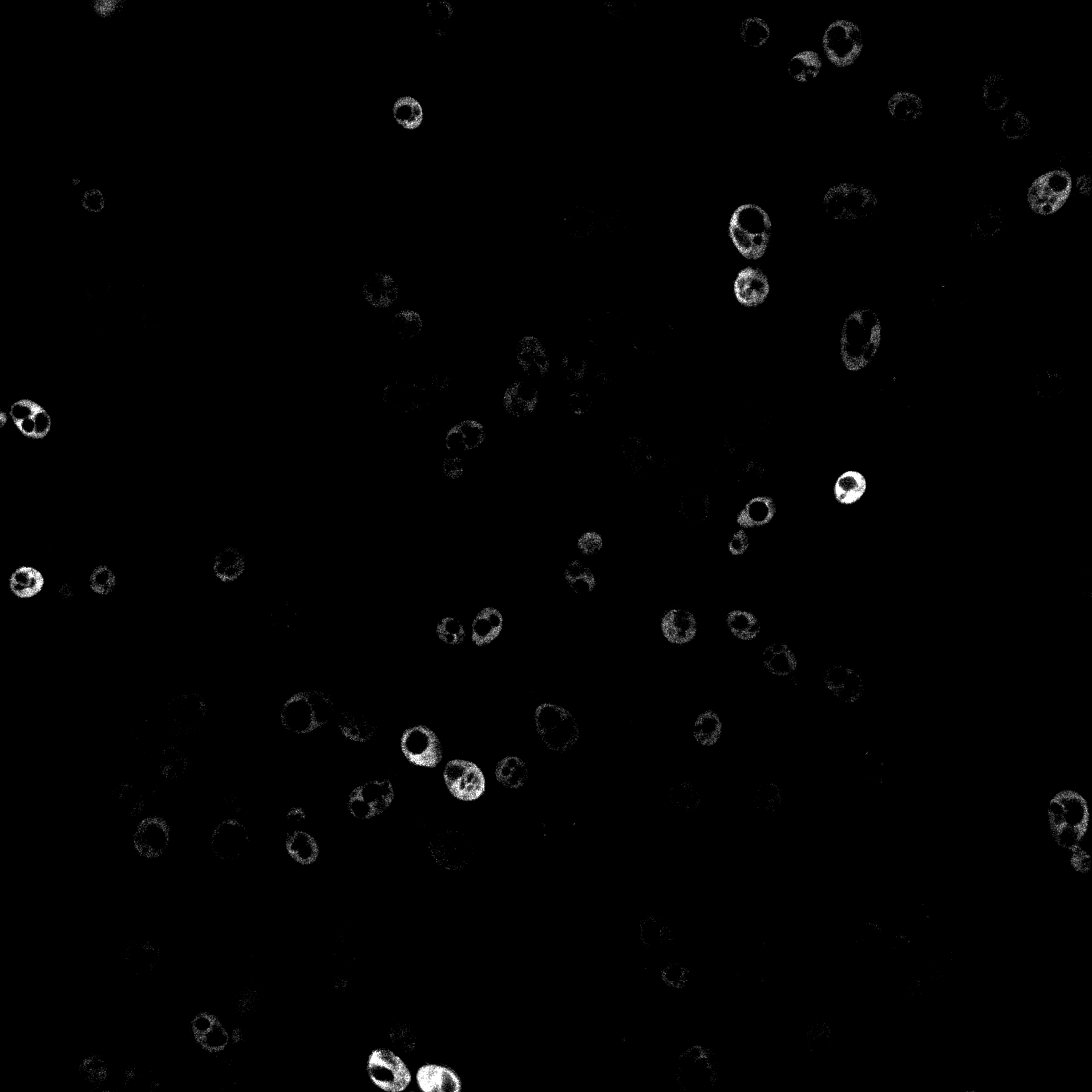

Supplement: Supplementary file 7 — Source Data Fig. 6 [file 44319_2023_55_MOESM7_ESM.zip › Figure 6/6D/Microsocpy_1D11D13D19D/Atg1-Atg13_44D_GFP/Atg1-Atg1344D-GFP_1D11D13D19D.tif]

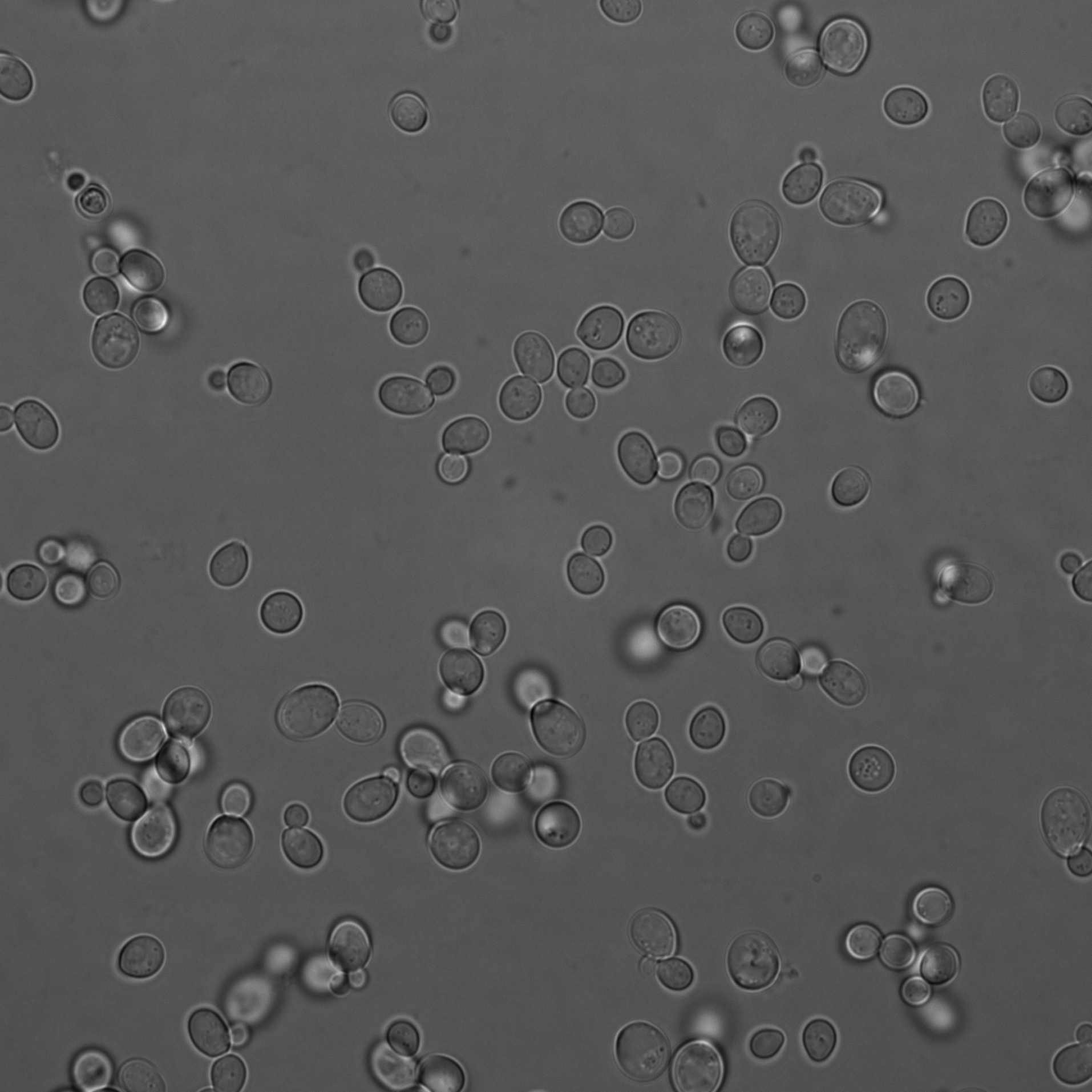

Supplement: Supplementary file 7 — Source Data Fig. 6 [file 44319_2023_55_MOESM7_ESM.zip › Figure 6/6D/Microsocpy_1D11D13D19D/Atg1-Atg13_44D_GFP/Atg1-Atg1344D-GFP_BF_1D11D13D19D.tif]

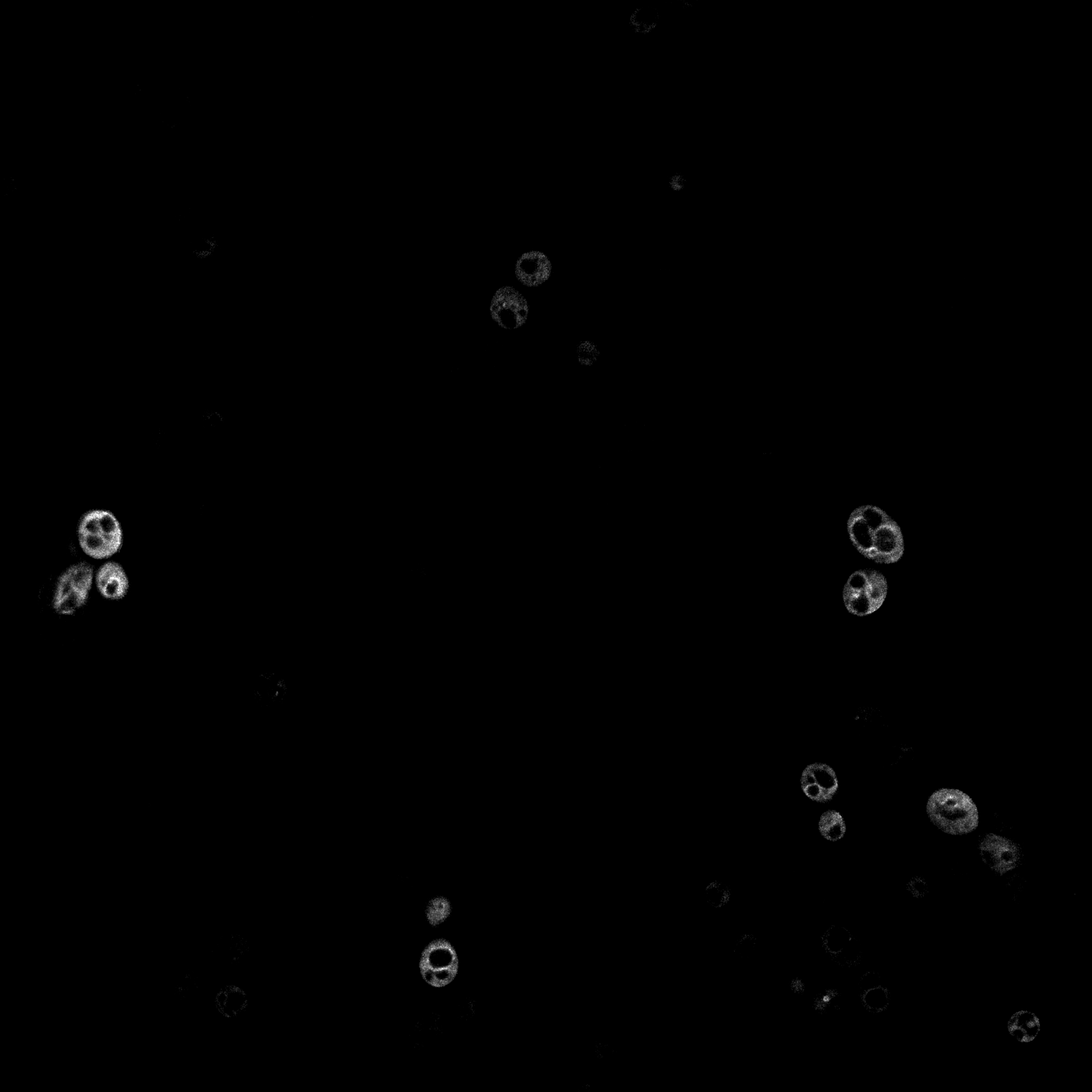

Supplement: Supplementary file 7 — Source Data Fig. 6 [file 44319_2023_55_MOESM7_ESM.zip › Figure 6/6D/Microsocpy_1D11D13D19D/Atg1-Atg13_MD_GFP/Atg1_Atg13MD_GFP_Green_1D11D13D19D.tif]

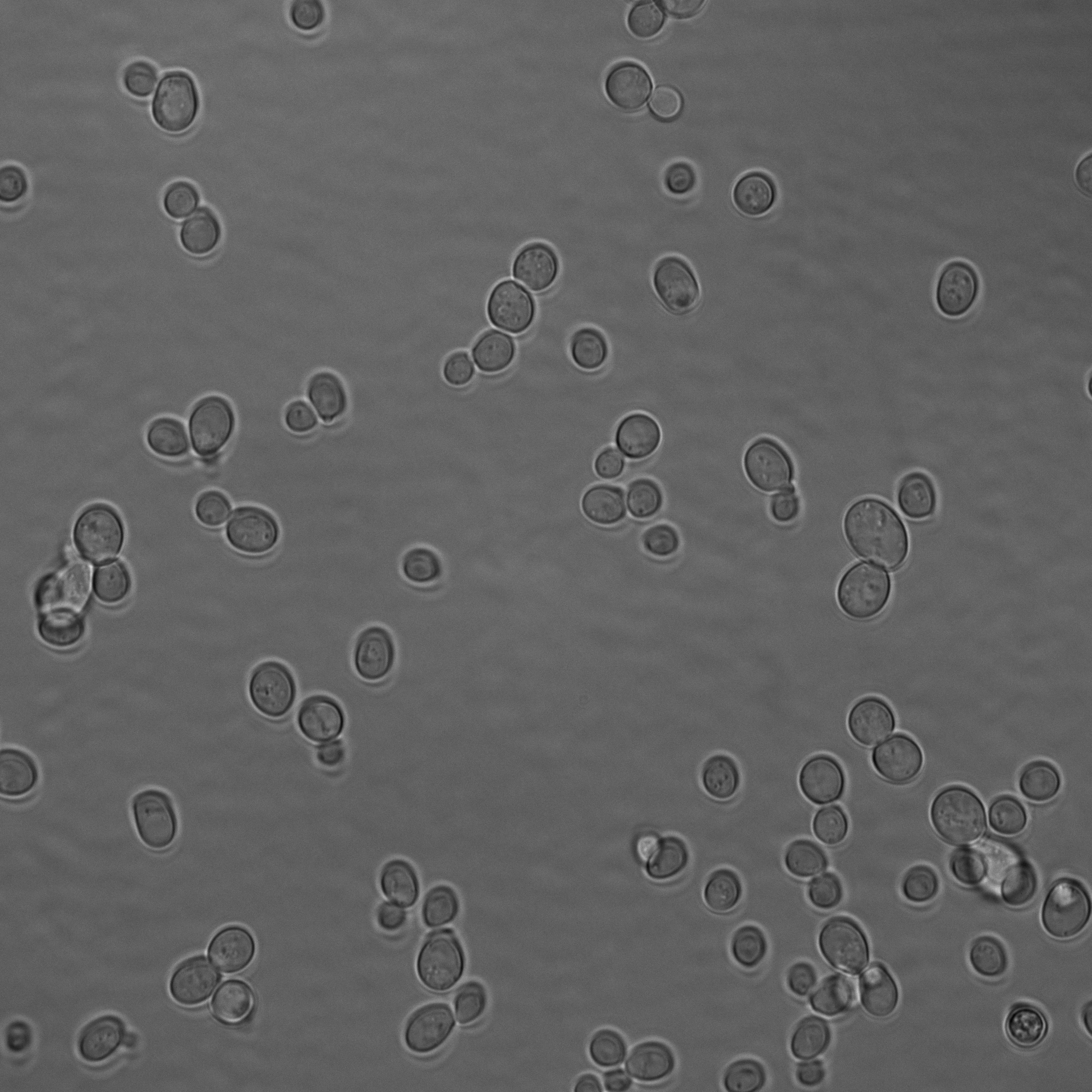

Supplement: Supplementary file 7 — Source Data Fig. 6 [file 44319_2023_55_MOESM7_ESM.zip › Figure 6/6D/Microsocpy_1D11D13D19D/Atg1-Atg13_MD_GFP/Atg1_Atg13MD_GFP_BF_1D11D13D19D.tif]

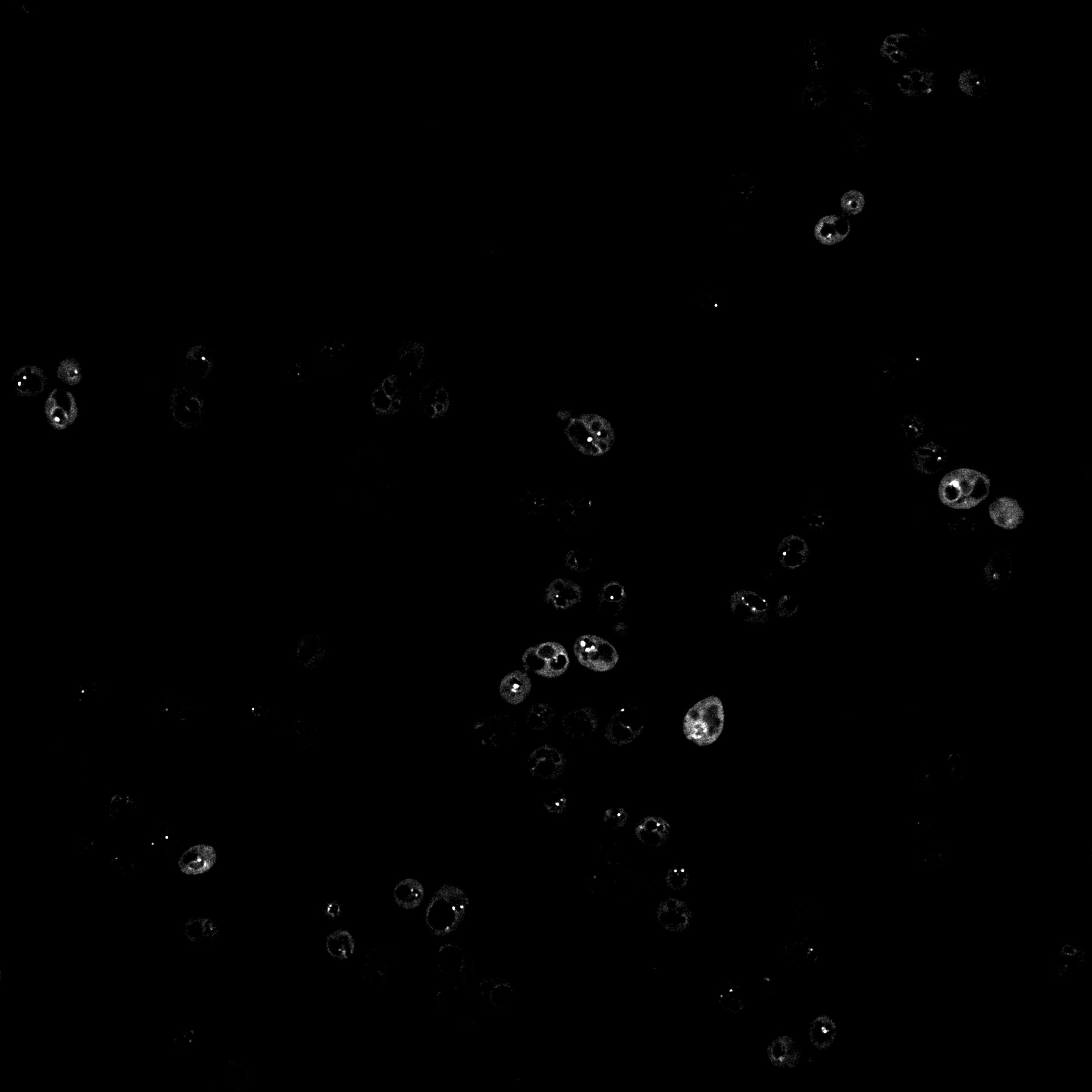

Supplement: Supplementary file 7 — Source Data Fig. 6 [file 44319_2023_55_MOESM7_ESM.zip › Figure 6/6D/Microsocpy_1D13D19D/Atg1_Atg13_GFP/GFP.tif]

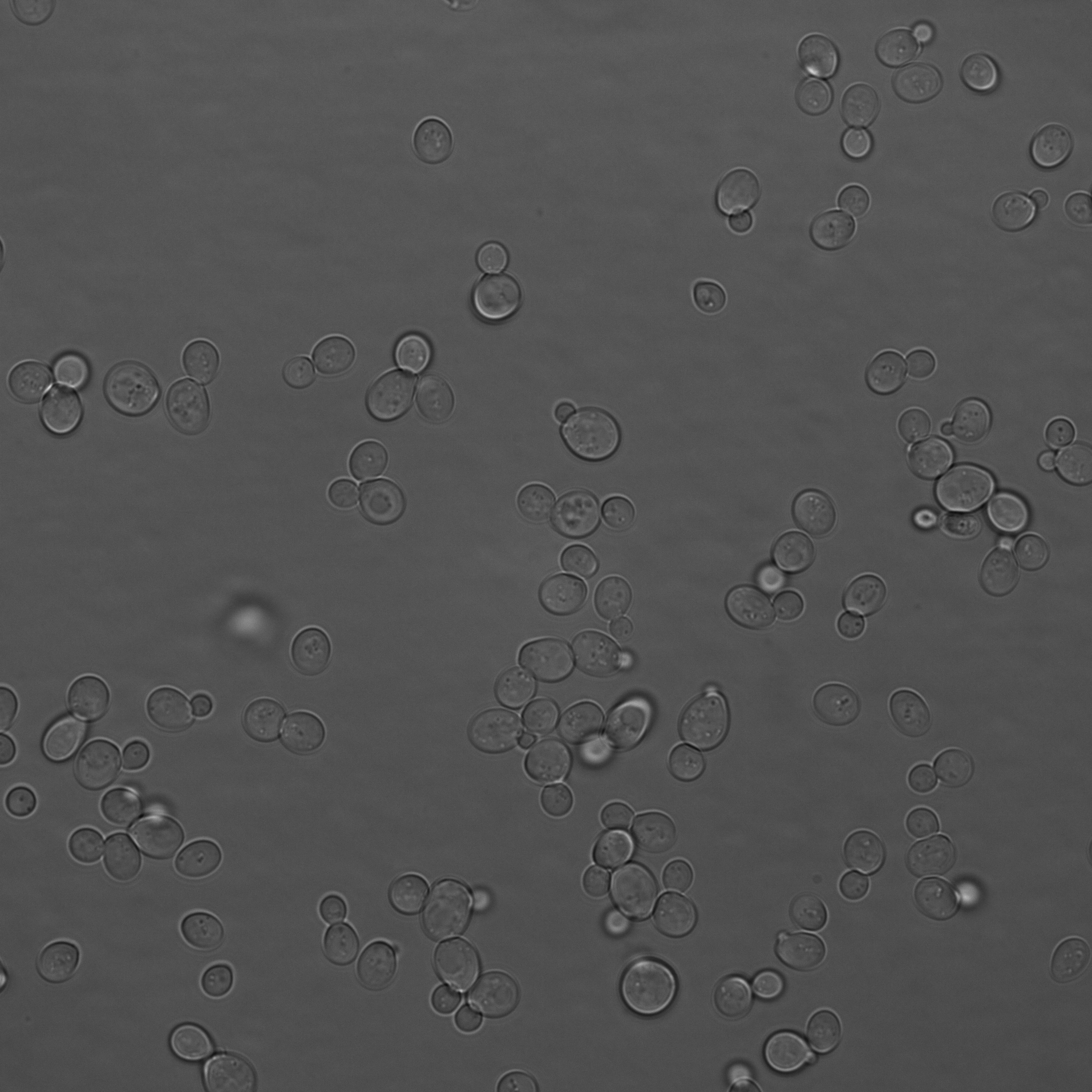

Supplement: Supplementary file 7 — Source Data Fig. 6 [file 44319_2023_55_MOESM7_ESM.zip › Figure 6/6D/Microsocpy_1D13D19D/Atg1_Atg13_GFP/BF.tif]

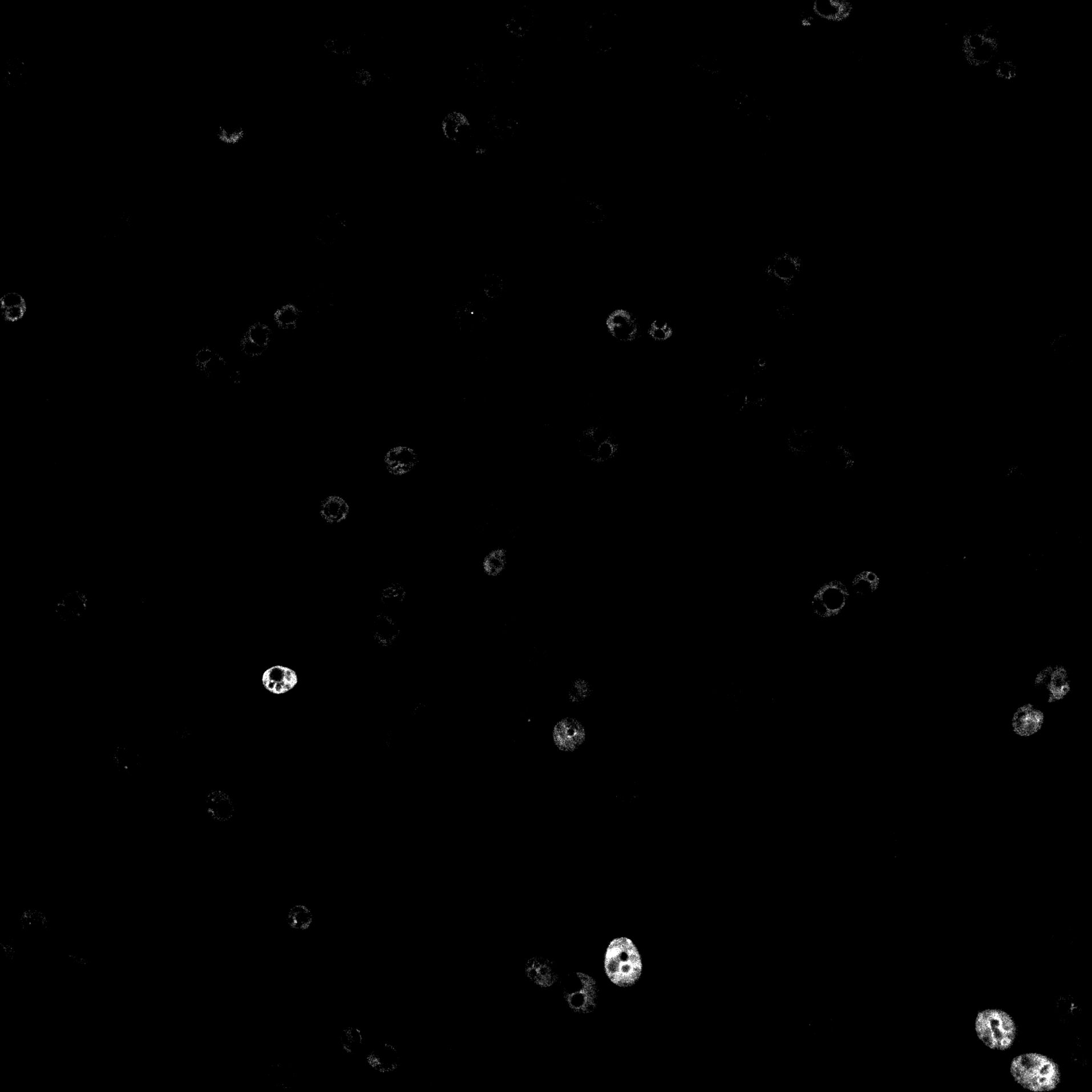

Supplement: Supplementary file 7 — Source Data Fig. 6 [file 44319_2023_55_MOESM7_ESM.zip › Figure 6/6D/Microsocpy_1D13D19D/Atg1-Atg13MD/GFP.tif]

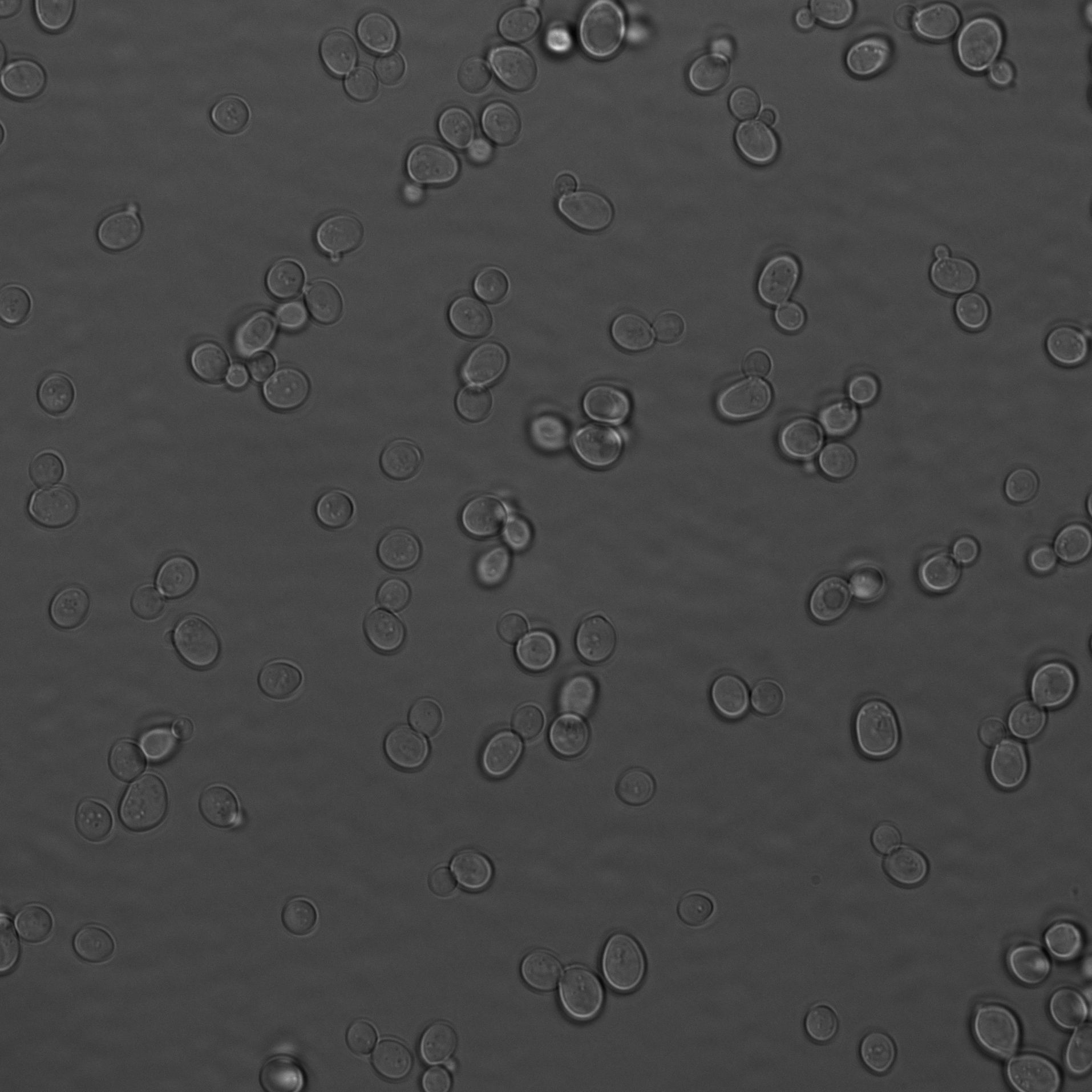

Supplement: Supplementary file 7 — Source Data Fig. 6 [file 44319_2023_55_MOESM7_ESM.zip › Figure 6/6D/Microsocpy_1D13D19D/Atg1-Atg13MD/BFP.tif]

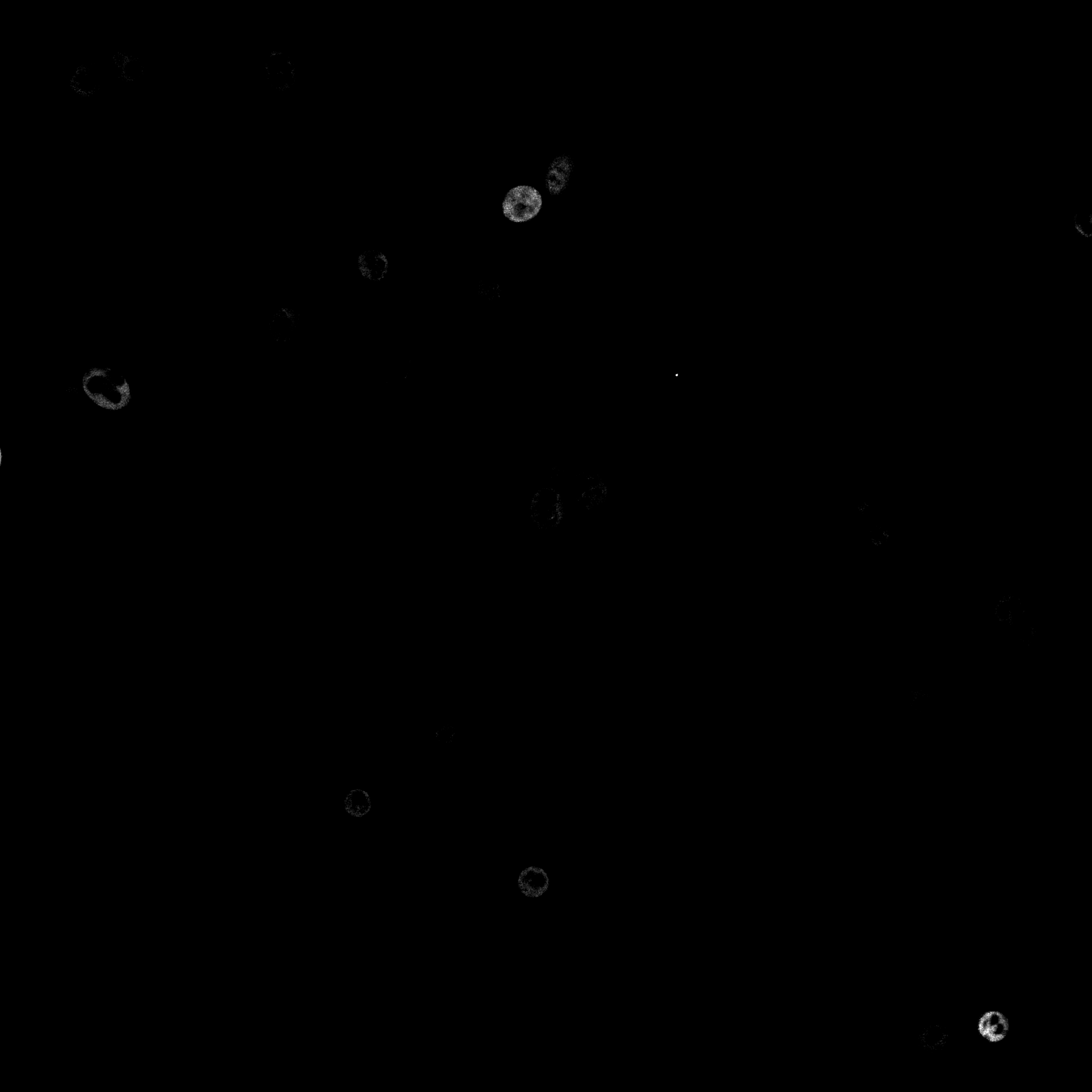

Supplement: Supplementary file 7 — Source Data Fig. 6 [file 44319_2023_55_MOESM7_ESM.zip › Figure 6/6D/Microsocpy_1D13D19D/Atg1-Atg13_44D/GFP.tif]

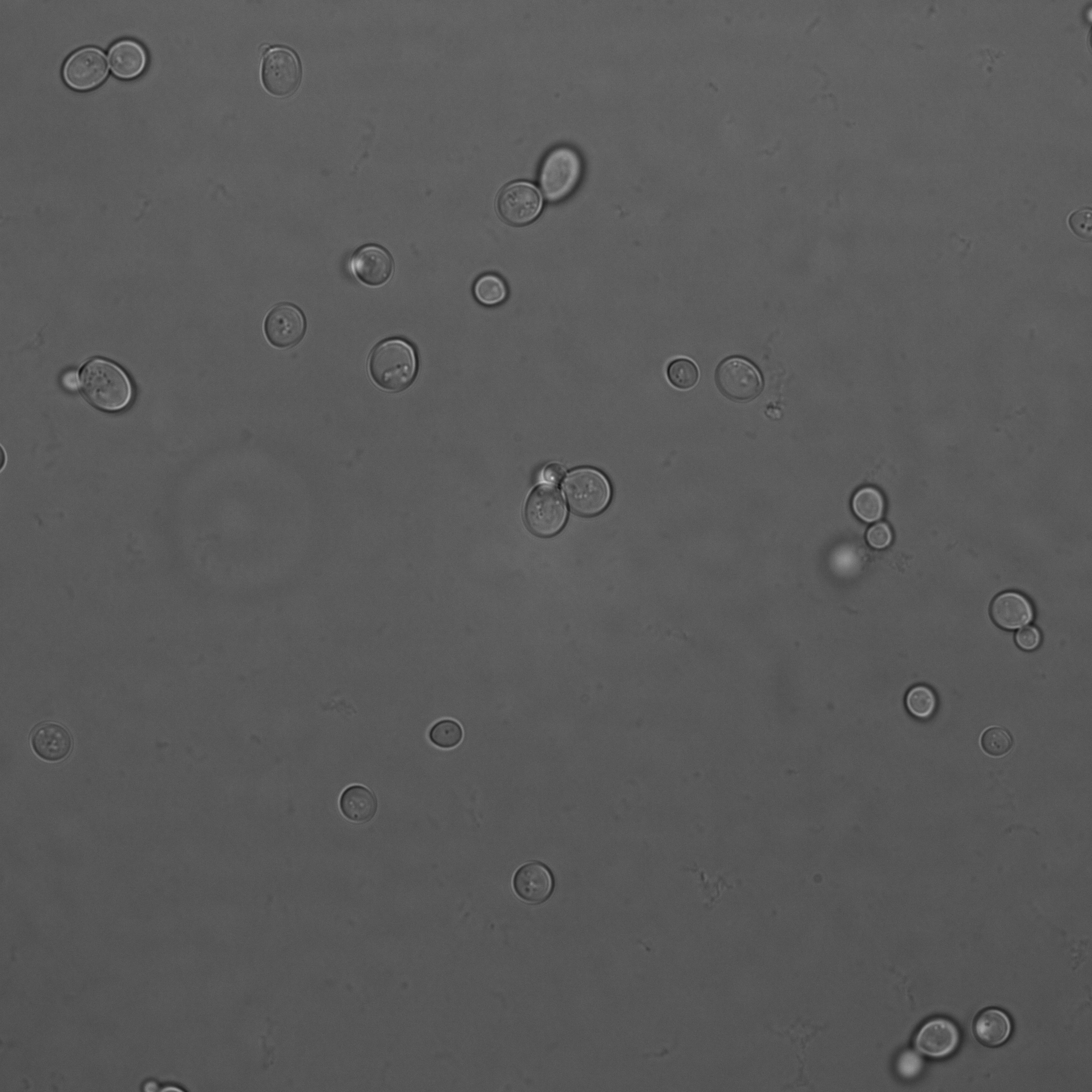

Supplement: Supplementary file 7 — Source Data Fig. 6 [file 44319_2023_55_MOESM7_ESM.zip › Figure 6/6D/Microsocpy_1D13D19D/Atg1-Atg13_44D/BF.tif]

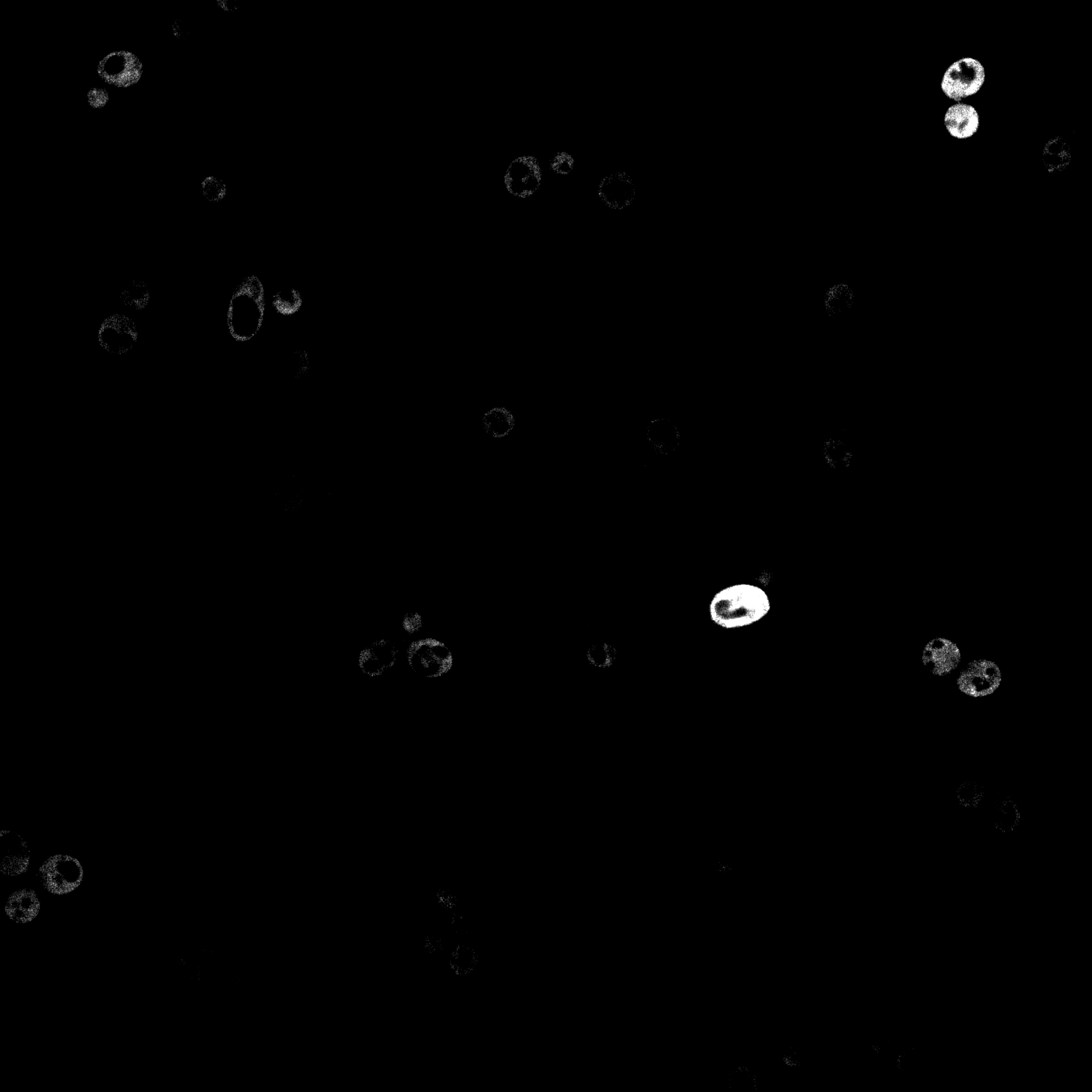

Supplement: Supplementary file 7 — Source Data Fig. 6 [file 44319_2023_55_MOESM7_ESM.zip › Figure 6/6D/Microsocpy_1D13D19D/Atg1-GFP/GFP.tif]

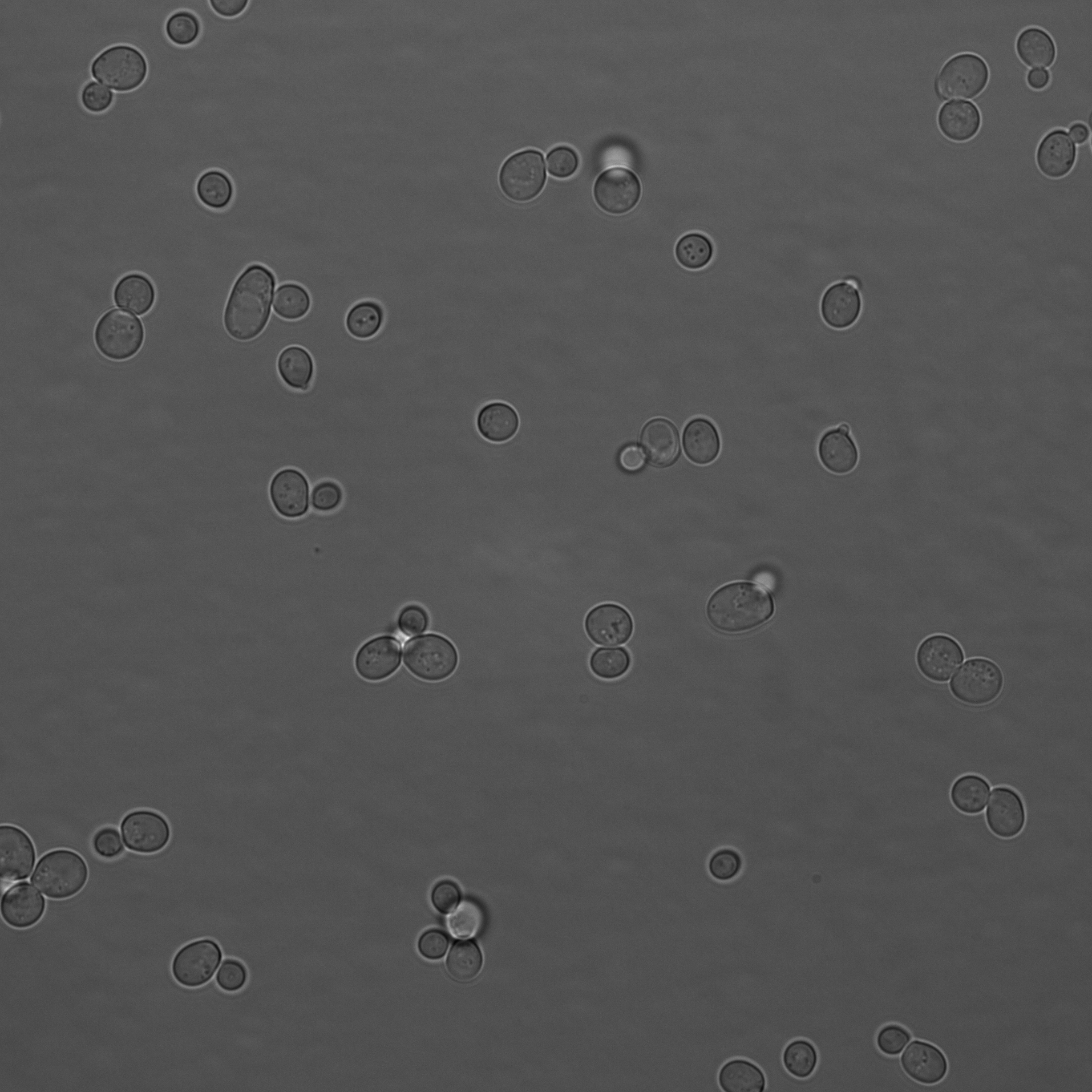

Supplement: Supplementary file 7 — Source Data Fig. 6 [file 44319_2023_55_MOESM7_ESM.zip › Figure 6/6D/Microsocpy_1D13D19D/Atg1-GFP/BF.tif]
